# SYNPRED: prediction of drug combination effects in cancer using different synergy metrics and ensemble learning

**DOI:** 10.1093/gigascience/giac087

**Published:** 2022-09-26

**Authors:** António J Preto, Pedro Matos-Filipe, Joana Mourão, Irina S Moreira

**Affiliations:** Center for Neuroscience and Cell Biology, University of Coimbra, 3004-504 Coimbra, Portugal; PhD Programme in Experimental Biology and Biomedicine, Institute for Interdisciplinary Research (IIIUC), University of Coimbra, Casa Costa Alemão, 3030-789 Coimbra, Portugal; Center for Neuroscience and Cell Biology, University of Coimbra, 3004-504 Coimbra, Portugal; CNC—Center for Neuroscience and Cell Biology, CIBB—Center for Innovative Biomedicine and Biotechnology, 3004-504 Coimbra, Portugal; Department of Life Sciences, University of Coimbra, Calçada Martim de Freitas, 3000-456 Coimbra, Portugal; CNC—Center for Neuroscience and Cell Biology, CIBB—Center for Innovative Biomedicine and Biotechnology, 3004-504 Coimbra, Portugal

**Keywords:** ensemble learning, interpretability, omics, biophysics, drug synergy, cancer

## Abstract

**Background:**

In cancer research, high-throughput screening technologies produce large amounts of multiomics data from different populations and cell types. However, analysis of such data encounters difficulties due to disease heterogeneity, further exacerbated by human biological complexity and genomic variability. The specific profile of cancer as a disease (or, more realistically, a set of diseases) urges the development of approaches that maximize the effect while minimizing the dosage of drugs. Now is the time to redefine the approach to drug discovery, bringing an artificial intelligence (AI)–powered informational view that integrates the relevant scientific fields and explores new territories.

**Results:**

Here, we show SYNPRED, an interdisciplinary approach that leverages specifically designed ensembles of AI algorithms, as well as links omics and biophysical traits to predict anticancer drug synergy. It uses 5 reference models (Bliss, Highest Single Agent, Loewe, Zero Interaction Potency, and Combination Sensitivity Score), which, coupled with AI algorithms, allowed us to attain the ones with the best predictive performance and pinpoint the most appropriate reference model for synergy prediction, often overlooked in similar studies. By using an independent test set, SYNPRED exhibits state-of-the-art performance metrics either in the classification (accuracy, 0.85; precision, 0.91; recall, 0.90; area under the receiver operating characteristic, 0.80; and F1-score, 0.91) or in the regression models, mainly when using the Combination Sensitivity Score synergy reference model (root mean square error, 11.07; mean squared error, 122.61; Pearson, 0.86; mean absolute error, 7.43; Spearman, 0.87). Moreover, data interpretability was achieved by deploying the most current and robust feature importance approaches. A simple web-based application was constructed, allowing easy access by nonexpert researchers.

**Conclusions:**

The performance of SYNPRED rivals that of the existing methods that tackle the same problem, yielding unbiased results trained with one of the most comprehensive datasets available (NCI ALMANAC). The leveraging of different reference models allowed deeper insights into which of them can be more appropriately used for synergy prediction. The Combination Sensitivity Score clearly stood out with improved performance among the full scope of surveyed approaches and synergy reference models. Furthermore, SYNPRED takes a particular focus on data interpretability, which has been in the spotlight lately when using the most advanced AI techniques.

## Background

Cancer, a heterogeneous group of diseases, is one of the leading causes of mortality and the most significant barrier to increasing life expectancy worldwide. The International Agency for Research on Cancer estimates that, by 2040, approximately 30.2 million new cancer cases and 16.3 million deaths will be reported, mainly due to the population's growth and aging [1]. One of the significant contributors to this disease's global burden is the development of therapy resistance and, consequently, tumor relapse. Drug resistance in cancer is a multifactorial problem driven by the tumor microenvironment and genetic and nongenetic/epigenetic mechanisms that, along with cell plasticity, contribute to tumor heterogeneity [2]. In clinical settings, this problem is minimized with a combination of drugs administered together or in sequence (i.e., polytherapy). Targeting multiple components of different or interconnected cancer pathways is an efficient strategy to block vital biological processes [3, 4].

Drug combinations with a synergistic effect (i.e., when the total therapeutic effect of both drugs is greater than the expected additive monotherapy effect) [5] were successfully developed and applied in the treatment of different types of tumors, such as human epidermal growth factor receptor 2–positive breast cancer [6], chronic myeloid leukemia [7], prostate cancer [8], or BRAF-mutated tumors [9]. Nevertheless, this simultaneous administration can also result in a reduced therapeutic effect and possible toxicity (designated antagonism) or in the same beneficial effect when compared with the expected additive monotherapy effect (additivity) [5]. The experimental identification of successful synergistically effective combinations is a well-known time-consuming and expensive task. Therefore, there is still a significant need for efficient and user-friendly computational methods, available in easy to use interfaces, to complement and speed up the traditional approaches by predicting the best synergistic drug combinations [10, 11].

In the past years, the development and improvement of high-throughput technologies and computational tools boosted the use of large volumes of multiomics data (e.g., genomic, transcriptomic, proteomic) essential to dissect and uncover the complex molecular signatures of cancer. Machine learning (ML) algorithms have attracted particular attention for their ability to learn new associations and extract valuable insights from this type of data. A few ML models based on extreme gradient boosting, random forest, elastic nets, support vector machine, and naive Bayes were already developed to predict the best combination of anticancer drugs by the integration of omics data with chemoinformatic properties of drugs or network information of their targets [12–15]. Likewise, deep learning (DL) implemented via deep neural networks (DNNs) was particularly useful in dealing with the high multidimensionality of omics data in supervised and unsupervised contexts. DL classification and regression models such as AuDNNsynergy [16], DeepDDS [17], DeepSynergy [18], DeepSignalingSynergy [19], Matchmakers [20], TranSynergy [21], or the work by Xia and colleagues [22] were recently developed for drug combination prediction. Nearly all the surveyed works developed drug synergy prediction models based upon a single reference model, which is in most cases the Loewe reference model [14, 16–18, 20, 21]. Currently, there is a wide scope of well-studied available reference models, including the Bliss independence [23], highest single agent (HSA) [24], Loewe additivity [25, 26], and zero interaction potency (ZIP) [27]. Furthermore, recently Malyutina et al. [15] developed the Combination Sensitivity Score (CSS), which measures drug combination synergy using their IC_50_. As such, this led us to the question of whether the development of a novel prediction approach should be based solely upon a single reference model. Besides, most of the available web interfaces such as DECREASE [28] or DrugComb [29] require for synergy prediction the upload of a full or partial mandatory dose–response matrix (experimentally determined), which hinders its systematic use by the scientific community and handicaps its usefulness.

**Figure 1:**
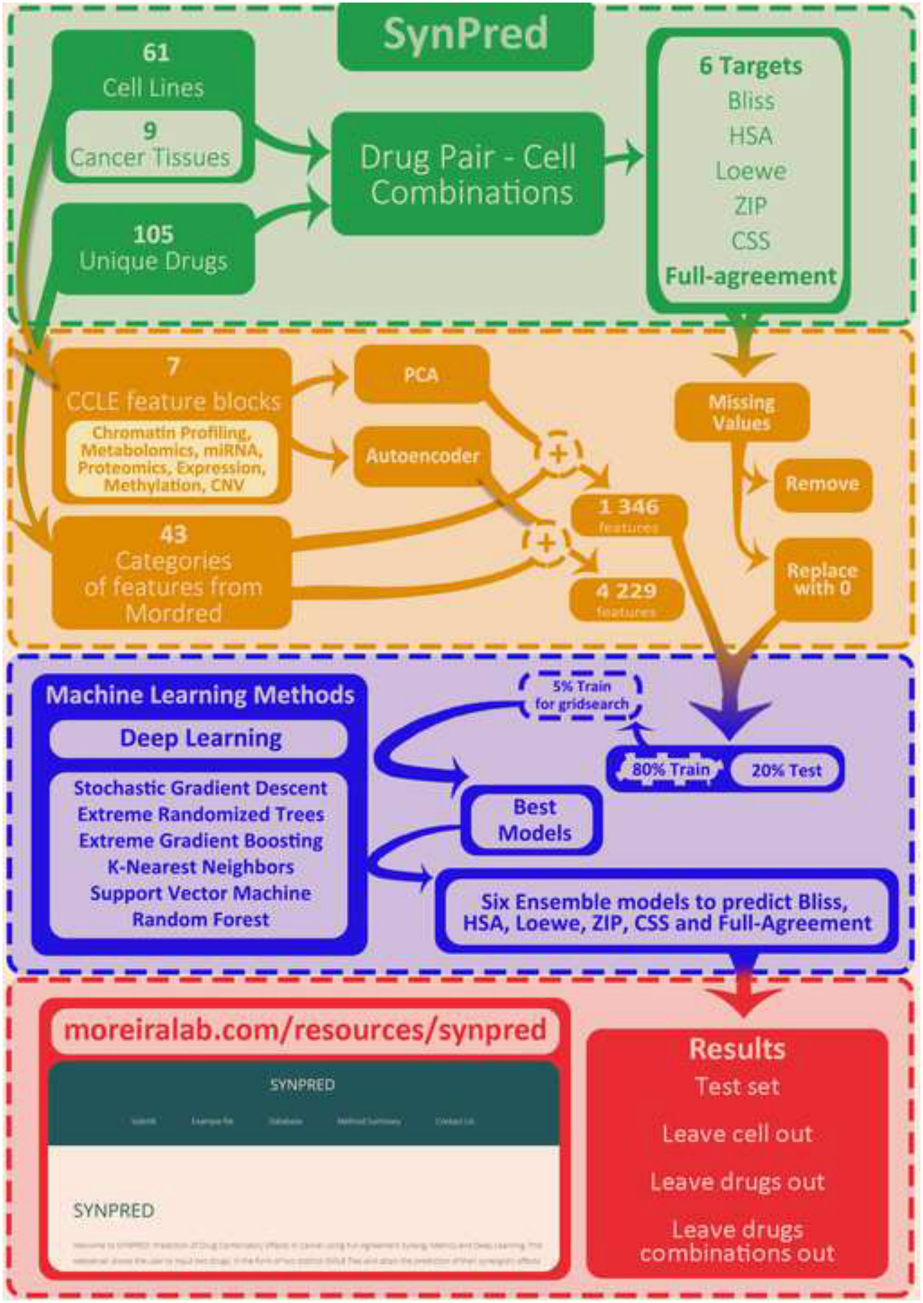
SYNPRED workflow summary. Green: Dataset construction. The National Cancer Institute—A Large Matrix of Anti-Neoplastic Agent Combinations database (phenotypic data) and the Cancer Cell Line Encyclopedia (CCLE) (multiomics data) were used for this purpose. Four reference models (Bliss, HSA, Loewe, ZIP) in addition to the CSS were used to quantify the combination degree and retrieve a full agreement between all metrics. Orange: Feature extraction and data preprocessing. Included normalization and dimensionality reduction using autoencoder or principal component analysis (PCA). Blue: Grid search and prediction model development using a training set. Red: Model evaluation using different classification and regression metrics in an independent test set and 3 different scenarios: (i) leave cell out dataset, (ii) leave drugs out dataset, and (iii) leave drug combinations out dataset.

To overcome the current problems found in the field, we developed SYNPRED (SYNergy PREDiction), a collection of *in silico* ensemble classification and regression models that considers several synergy references models: Bliss, Loewe, HSA, ZIP, and CSS. It was developed by integrating multiomics features of cell lines and phenotypic and biophysical data, particularly physicochemical and structural features of drugs. SYNPRED displays a good predictive performance and inherently addresses the issue at a broader and more profound angle than the existing approaches, which generally focus on either classification or a single regression task and typically use a single synergy reference model. We made available the stand-alone deployment at https://github.com/MoreiraLAB/synpred, which allows the user the opportunity to undergo bulk prediction with SYNPRED. Additionally, for the first time, a user-friendly web-based application was assembled and made freely available online at http://www.moreiralab.com/resources/synpred/ to predict drug combinations, requiring only the upload of the 2 drugs' simplified molecular-input line-entry system (SMILEs) to be tested. This interactive platform will allow users with different backgrounds, from scientists to clinicians, to test, reproduce, and validate our models and data. The workflow used for the development of SYNPRED is depicted in Fig. 1.

## Data and Methods

### Experimental drug combination phenotypic data

Drug combination phenotypic data were acquired via bulk-download from the largest-to-date dataset from National Cancer Institute—A Large Matrix of Anti-Neoplastic Agent Combinations (NCI ALMANAC) through https://wiki.nci.nih.gov/display/NCIDTPdata/NCI-ALMANAC [30]. To this date, the dataset includes phenotypic data of tested cancer cell lines (growth percentage) of 105 unique drugs approved by the US Food and Drug Administration (FDA). These drugs were tested in combination against 61 cell lines from 9 cancer types currently included in the NCI [31, 32], comprising a total of 311,466 drug pair/cell line combinations. Drug sensitivity assays included in NCI ALMANAC were performed at the NCI's Frederick National Laboratory for Cancer Research, the Stanford Research Institute, and the University of Pittsburgh. Briefly, for each assay, cells were cultivated for 48 hours in a 3 × 3 or a 5 × 3 concentration matrix (different concentration values for each drug in combination) and the endpoint determined by Sulforhodamine B or CellTiter-Glo [30]. From these records, the authors retrieved the cell growth percentage at each drug concentration point, which corresponds to the percentage of growth of the cell lines in the presence of each combination, yielding a final viability assessment.

### Combination scores and class definition

The phenotypic data from high-throughput drug combination screens were retrieved from DrugComb [29]. DrugComb extends its synergy metrics calculations from “SynergyFinder” [33], which leverages the percentage of cell growth included in the dataset to assess the degree of combination for each pair of drug concentrations by using several synergy reference models. As such, only the most well-studied synergy reference models described in the literature were included as they were the only ones that met the criteria of characterizing the effects of a drug pair on a cell line with a final single synergy score. This approach narrowed down our options to the 4 most well-known synergy reference models: Bliss independence (Equation 1) [23], Loewe additivity (Equation 2) [25,26], HSA (Equation 3) [24], and ZIP (Equation 4) [27]. In addition to the mentioned synergy reference models, we also used the CSS metric [15], a higher sensitivity score [29].

(1) \begin{equation*}
yBliss\; = \;y1 + y2 - y1y2
\end{equation*}

Bliss independence model: *y*Bliss is the Bliss response, *y*1 is the drug 1 response, and *y*2 is the drug 2 response.

(2) \begin{equation*}
yLoewe\; = \frac{{Emin + Emax{{\left( {\frac{{x1 + x2}}{m}} \right)}^\lambda }}}{{1 + {{\left( {\frac{{x1 + x2}}{m}} \right)}^\lambda }}}\; \end{equation*}

Loewe additivity model: *y*Loewe is the Loewe response, *E*min is the minimum drug response, *E*max is the maximum drug response, *m* is the dose that produces a midpoint effect between *E*min and *E*max, *λ* is the shape parameter indicating the slope of the curve, *x*1 is the drug 1 dose, and*x*2 is the drug 2 dose.

(3) \begin{equation*}
yHSA\; = \;{\mathrm{max}}\left( {y1,y2} \right) \end{equation*}

HSA model: *y*HSA is the HSA response, *y*1 is the drug 1 response, and*y*2 is the drug 2 response.

(4) \begin{equation*}
yZIP\; = \frac{{{{\left( {\frac{{x1}}{{m1}}} \right)}^{\lambda 1}}}}{{1 + {{\left( {\frac{{x1}}{{m1}}} \right)}^{\lambda 1}}}}\; + \frac{{{{\left( {\frac{{x2}}{{m2}}} \right)}^{\lambda 2}}}}{{1 + {{\left( {\frac{{x2}}{{m2}}} \right)}^{\lambda 2}}}} - \left( {\frac{{{{\left( {\frac{{x1}}{{m1}}} \right)}^{\lambda 1}}}}{{1 + {{\left( {\frac{{x1}}{{m1}}} \right)}^{\lambda 1}}}}*\frac{{{{\left( {\frac{{x2}}{{m2}}} \right)}^{\lambda 2}}}}{{1 + {{\left( {\frac{{x2}}{{m2}}} \right)}^{\lambda 2}}}}} \right) \end{equation*}

ZIP model: *y*ZIP is the ZIP response, *x*1 is the drug 1 dose, *x*2 is the drug 2 dose, *m*1 is the dose that produces a midpoint effect for drug 1, *m*2 is the dose that produces a midpoint effect for drug 2, *λ*1 is the shape parameter indicating the slope of the curve for drug 1, and *λ*2 is the shape parameter indicating the slope of the curve for drug 2.

**Figure 2:**
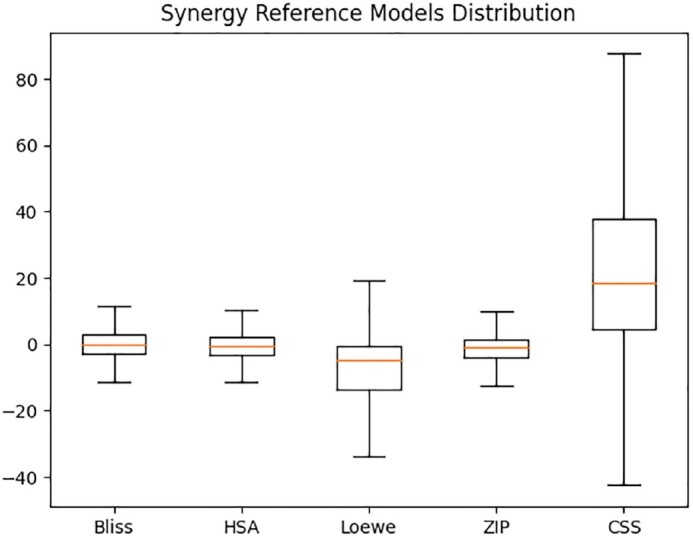
Box plot representing the distribution of synergy scores (y-axis) with respect to the 5 reference models: Bliss, HSA, Loewe, ZIP, and CSS (x-axis). The black boxes represent the difference between the upper 75% and the lower 25% quartiles (interquartile range); the horizontal orange line is the median; the whiskers are the lower and upper values that are not outliers or extremes (not represented as some of these values are off range).

Having computed Bliss, HSA, Loewe, ZIP, and CSS, a binary classifier was first developed to identify the type of combinatory effect present in each drug pair–cell line sample, where the values above the threshold (0, as defined for each metric by SynergyFinder [33]) (https://synergyfinder.fimm.fi/synergy/synfin_docs/) were defined as synergistic, and the remaining ones were classified as nonsynergistic. The dataset used for classification training considered full-agreement combination assessment (i.e., we only kept the instances on which combination classification was the same across the 4 previous reference predictors). For the dataset used, this process yielded 29,779 synergistic samples and 9,029 nonsynergistic samples. For the regression model deployment, we used the values attained directly from DrugComb to each synergy reference model (Bliss, HSA, Loewe, ZIP) as well as CSS. Most synergy reference model values were in similar scales (Loewe = [−116.63, 86.69], ZIP = [−36.08, 66.66], HSA = [−81.75, 64.29], Bliss = [−77.07, 78.65]) (Fig. 2, Figs. W1–W6 of the SYNPRED webserver). CSS stood in the interval [−54.05, 99.84], albeit with larger interquartile distances than the synergy reference models.

### Drug molecular descriptors

Each drug included in NCI ALMANAC was analyzed to extract its physicochemical and structural features. A SMILE representation of the drugs was acquired from PubChem [34]. SMILEs were then used to mine molecular descriptors using the Python package “Mordred” (Version 1.1.2) [35]. In total, an array of 1,613 numeric features of 43 different categories was retrieved, making a 2-dimensional molecular description of the drugs. Feature arrays comprising nonnumerical attributes or displaying zero variance were deleted. This preprocessing left 586 features describing each drug included in the NCI ALMANAC, distributed across 28 categories (Table 1). The resulting features were subjected to normalization by removing the mean and scaling to unit variance with scikit-learn's StandardScaler [36].

**Table 1:** Number of features according to the molecular descriptor category of Mordred. Features are categorized as Energetic (E), Pharmacological (P), Structural (S), or Miscellaneous (M—in case of evaluating characteristics of multiple fields).

**Number of features per descriptor category**					
E	Acidity/Basicity	2	S	Information Content	36
P	ADME	3	S	Molecular Complexity	1
S	Aromatics	2	P	Molecular Operating Environment	51
S	Atom Count	16	S	Molecule Graph	5
S	Atom-Bond Connectivity	2	S	Path Count	21
M	Autocorrelation	180	E	Polarizability	2
S	Bond Count	9	S	Ring Count	66
E	Atomic Orbitals	10	S	Rotatable Bonds	1
S	Chirality	38	S	Topological Charges	21
S	Constitutional	14	S	Topological Index	7
E	Energy State	68	S	Topological Polar Surface Area	2
S	Fragment Complexity	1	S	Walk Counts	21
S	Framework	1	S	Weight	2
S	Hydrogen Bonds	2	M	Wildman–Crippen	2

### Omics data of cancer cell lines

Omics data (expression, copy number variation, methylation, global chromatin profiling, metabolomics, microRNA, proteomic profiling) describing the cancer cell lines were acquired via bulk download from the Cancer Cell Line Encyclopedia (CCLE) (https://sites.broadinstitute.org/ccle/) [37]. The number of cell lines included in the CCLE varies depending on the type of omics data available at the time. Correspondence of cell line IDs between the NCI ALMANAC and CCLE was performed according to data available at the Swiss Institute of Bioinformatics Cellosaurus website [38]. According to the affected tissue, annotations acquired through Cellossaurus split the CCLE cell lines into 21 different cancer types. In agreement with the original publications [37, 39], expression data were obtained through RNA sequencing and processed to obtain level expression in transcripts per million by the expectation-maximization algorithm (file: CCLE_RNAseq_rsem_genes_tpm_20180929.txt.gz). Copy number variation (CNV) data were acquired from the Affymetrix SNP6.0 Arrays (file: CCLE_copynumber_byGene_2013–12-03.txt.gz). Copy numbers were normalized by the most similar HapMap normal samples [40]. Segmentation of normalized log_2_ (CN/2) ratios was achieved using the circular binary segmentation algorithm [37, 41]. Methylation data were derived by quantifying CpG islands using Reduced Representation Bisulfite Sequencing (file: CCLE_RRBS_tss_CpG_clusters_20181022.txt.gz). Global chromatin profiling was attained using multiple reaction monitoring for 42 combinations of histone marks (file: CCLE_GlobalChromatinProfiling_20181130.csv). Metabolomics data were acquired in parallel with global chromatin profiling by reporting the abundance measures of 225 metabolites (file: CCLE_metabolomics_20190502.csv). MicroRNA associated with cancer dependencies was correlated, regarding 734 microRNAs, with the Achilles gene dependency dataset. Protein profiling was measured with Reverse Phase Protein Arrays for 213 antibodies (file: CCLE_RPPA_20181003.csv) [39].

### Dimensionality reduction of omics data

Data were normalized by removing the mean and scaling to unit variance with scikit-learn's StandardScaler [36]. Due to the omics data's high complexity, we performed dimensionality reduction to minimize the noise introduced in the dataset by highlighting the essential features. The datasets already described were used to build and train a multilayer perceptron (MLP) autoencoder, an unsupervised artificial neural network (ANN) with a typical “hourglass” architecture, which is often used to perform dimensionality reduction in vast and high-dimensional datasets such as the ones observed with omics data [42–44]. This type of MLPs usually consists of 3 parts: an encoder that abstracts the input into hidden variables (i.e., a latent-space representation), a bottleneck layer that holds the smallest hidden layer (HL) (for purposes of dimensionality reduction, this is the layer that defines the size of the reduced dataset), and a decoder that reconstructs the original input data from the hidden data [45, 46]. Seven autoencoders, one for each of the CCLE feature blocks, were developed by using Keras with a TensorFlow for graphics processing unit (GPU) (Version 2.3.1) backend [47]. Each of the autoencoders comprised 7 layers, of which 5 were HLs. The input and output layers follow the number of available features in all cell lines, as displayed in Table 2. The number of nodes within the bottleneck layer of each of the 7 autoencoders (used for extraction of the encoded features) corresponds to the autoencoder's final number of features. The 2 HLs in each of the encoder and decoder sections vary in size according to the number of samples and features available (Supplementary Table S1). In this stage, all models used Adam [48] as an optimizer function with a learning rate of 0.001. Rectified linear unit (ReLU) activation function was used in all layers. Mean square error (MSE) was used as a loss function. The models were trained for 1,000, 250, or 100 epochs, depending on the dataset size (Supplementary Table S2). After training, each autoencoder's bottleneck layer was used to perform dimensionality reduction of the omics data according to Table 2.

**Table 2:** Number of features pertaining to the omics data and the corresponding amount for both the autoencoder and the principal component analysis (PCA) processing

Omics data	Number of available cell lines	Number of available features	Number of features after autoencoder	Number of features after PCA	Explained variance (PCA)
Expression	1,019	57,820	1,156	25	0.89
Copy number variation	1,043	23,316	466		0.91
Methylation	843	56,146	1,122		0.92
Global chromatin profiling	897	42	21		0.99
Metabolomics	928	225	112		0.99
MicroRNA	954	734	73		0.95
Proteomics	899	214	107		0.93

Principal component analysis (PCA), a commonly used method for dimensionality reduction [49], was also applied in the same datasets as the autoencoder, for which 25 principal components (PCs) were defined. It means that by using PCA, each dataset was transformed to yield only 25 features, totaling 175 features to describe each unique cell line. As shown in Table 2, each feature block from CCLE had its variance explained in a range from 0.89 to 0.99. Since the 7 blocks were used simultaneously for each sample, each cell line is thoroughly described by the components extracted with the PCA. Missing values (in both autoencoder and PCA) were processed by either dropping the sample entirely or replacing the missing values with zero.

### Model evaluation and performance metrics

After data acquisition and preprocessing, we gathered all datasets, and to evaluate the results in the most unbiased manner possible, we randomly isolated 3 datasets considering different scenarios:

- Leave cell out dataset: 3 randomly chosen cell lines belonging to different tissue types (regression dataset: 13,810 combinations; classification dataset: 1,396 synergistic and 429 nonsynergistic samples after processing the 13,810 combinations for full agreement) (for the tissue type classification, see Fig. W1 of the SYNPRED webserver).
- Leave drugs out dataset: 5 drugs with the majority belonging to different hierarchical clusters (regression dataset: 25,993 combinations; classification dataset: 2,934 synergistic and 622 nonsynergistic samples after processing the 25,993 combinations for full agreement) (for drug hierarchical clustering, see Fig. W8 of the SYNPRED webserver).
- Leave drug combinations out dataset: 5 drug combinations (regression dataset: 360 combinations; classification dataset: 74 synergistic and 6 nonsynergistic samples after processing the 360 combinations for full agreement).

After extracting the datasets for validation, we split the remaining data into training and test sets on an 80/20 ratio (Supplementary Table S3). As such, the training dataset was composed of 195,996 combinations to be used for regression tasks that, upon full agreement processing, yielded 20,291 synergistic and 6,419 nonsynergistic samples for classification tasks. The test set was composed of 48,999 combinations to be used for regression tasks, which, upon full agreement processing, yielded 5,084 synergistic and 1,553 nonsynergistic samples for classification tasks. The described data splitting was performed before any model training, thus ensuring all the prediction models’ performance evaluation is deployed on the same data. The binary classification models were evaluated through accuracy (acc), precision (prec), recall (rec), area under the receiver operating characteristic (AUROC), and F1-score as previously described [50]. The regression models were evaluated through the root mean square error (RMSE), mean squared error (MSE), mean absolute error (MAE) [51], Pearson and Spearman correlation coefficients [52].

### Development of ML models

#### Neural networks with Keras

The classification and regression neural networks were fully developed using Keras with a TensorFlow (Version 2.3.1) backend [47]. Weights were updated using the Adam optimizer [48] and a learning rate of 0.0001 along 125 epochs with binary cross-entropy (classification) and MSE (regression) as the loss functions. All the HLs were connected through ReLU activation, while the output layer was subject to sigmoid (classification) or linear activation (regression). As an initial approach, we performed a grid search for parameter optimization using 5% of the training set, fully detailed in the “Parameter optimization” section. The best-performing parameters were further selected, and used to train the models with the complete train dataset.

#### ML algorithms with scikit-learn

The datasets presented in this work were also trained with the most commonly used algorithms for synergy prediction tasks, namely, random forest (RF) [53], extreme randomized trees (ETC) [50, 54], support vector machines (SVMs) [55], stochastic gradient descent (SGD) [56], k-nearest neighbors (kNNs) [57], and extreme gradient boosting (XGBoost) [58]. The RF, ETC, SVM, SGD, and kNN models were built using the Python package “SciKit Learn” (Version 0.22.1) [36]. The XGBoost model was built using its dedicated package for Python (available at the Python Package Index as “xgboost”) [58]. These 6 algorithms were also subject to grid search for parameter optimization using 5% of the training set as described in the “Parameter optimization” section, with the best ones used to train the models with the full dataset.

#### Parameter optimization

To properly perform parameter optimization in all the algorithms described, a grid search was performed using in-house scripts for Keras DL models and scikit-learn's GridSearchCV with 3-fold cross-validation (for ML algorithms with scikit-learn). We used 5% of the training set [59], a value in agreement with subset usage for parameter optimization [60], since using the full training dataset would exponentially increase an already long task. For each of the Keras classification and regression DL models, we performed grid search with 192 runs with parameters covering the 4 available dimensionality reduction datasets (PCA, PCA_drop, autoencoder, autoencoder_drop), 12 different network architectures, and 4 different dropout rates (0.00, 0.25, 0.50, 0.75) (Supplementary Table S4). In the case of each of the 6 classification and regression ML models trained with scikit-learn, we used 820 runs, including different parameters and dataset combinations (Supplementary Table S5). Finally, for the 6 possible targets (full agreement, Bliss, HSA, Loewe, ZIP, and CSS), we trained each of the 6 ML models with the best corresponding performing parameters. We then assessed the best-performing architectures and dropout rates for the DL-based models. For each of the possible evaluation metrics, we then trained the best-performing parameters, which can lead to a different number of DL-based models depending on the synergy reference model used due to parameter overlap.

#### Ensemble algorithms

After selecting the previous best-performing models, we replaced the outliers with the average of the remaining prediction values. For some tasks, a few of the individual predictors had notably bad performance (mostly SGD and kNN). As such, we considered outliers the synergy prediction values above or below 10 times the average of the remaining prediction values; this was necessary to allow the ensemble neural networks to converge. These prediction values were used to constitute a new feature representation of the samples that could undergo ensemble model training. The ensemble models were first subjected to a new grid search for parameter optimization (Supplementary Table S6), taking the target probability of the selected algorithms as features, ultimately developing a neural network that worked as an ensemble method. This neural network had a learning rate of 0.0001, trained for 3 epochs, and used the Adam optimizer [48] and binary cross-entropy and MSE for classification and regression, respectively, as the loss functions. All the HLs were connected through ReLU activation, while the output layer was subject to sigmoid or linear activation for classification and regression, respectively. The best-performing ensemble models were trained with the prediction-based feature space.

### Feature contribution

To understand what were the top contributors for accurate predictions, we assessed their predictive power. For that, we needed first to break down the process of assessing feature contribution into 2 stages due to the dimensionality reduction of cell lines. First, since the best-performing dimensionality reduction approach was the PCA, we considered the explained variance by each of the features concerning the respective PC. This information was then extracted as an attribute from the PCA object using scikit-learn [36]. Second, we used the eli5 package [61], with Python deployment, to assess the final feature weight by deploying permutation importance [53], a method that allows iterative exclusion of each of the features, to assess its contribution to the predictive model. The permutation importance was deployed on the test set because it would not be possible to assess the feature contribution under unbiased conditions if the training set had been used. However, it is worth noting that this evaluation occurs after all model training; hence, it does not influence the test results.

### Benchmark

Benchmarking synergy prediction protocols is a very complicated process. As reviewed by Zagidullin et al. [29], the datasets available completely differ in the amount of information used, with DrugComb [29] assembling the most important ones (ALMANAC [30], ONEIL [62], FORCINA [63], CLOUD [64]). As shown by Kumar and Dogra [65], most authors used NCI ALMANAC data to train and the Loewe additivity synergy reference model [14, 16–18, 20, 21]. Furthermore, comparison to the available methodologies implies that authors adapt the published proposed DL architectures as these are not easily applied or not available in GitHub or similar platforms (e.g., pruning the data due to unavailability of a certain data modality, or changing the loss function to turn a model into a regressor).

As such, we followed a multistep approach to benchmark our pipeline:

- Comparison of DL architectures and simpler ML algorithms (RF, ETC, SVM, SGD, kNN, and XGBoost models) with ensemble approaches in 4 different test scenarios.
- DeepSynergy [18] architecture implementation and comparison using our independent test set and validation sets as this is one of the most common approaches. As described in the original study, we retrained a model using 2 hidden layers, the first with 8,192 and the second with 4,096 neurons. Furthermore, 2 dropout layers were added, the first with a 0.2 rate and the second with a 0.5 rate. The activation function used between the hidden layers was a hyperbolic tangent, and on the output layer, linear activation was used. This DeepSynergy implementation was trained over 250 epochs with a learning rate of 0.00001 and an Adam optimizer.
- Comparison with published methods for synergy calculations using both regression (12 models) and classification (13 models) approaches as reviewed by Kumar and Dogra [65].
- Comparison of our regression approaches to algorithms for which the training dataset was clearly available to make sure the comparison would be as fair as possible. As such, we compared to the Matchmakers’ algorithm [20] using the adapted DrugCombo (retrieved from Matchmakers’ [20] GitHub) and NCI ALMANAC complete datasets, which, in turn, enables us also to compare with DeepSynergy [18] and TreeCombo [12] as these were also evaluated by the authors [20]. Upon the data considered, we performed our own feature extraction, as described in the SynPred pipeline. Thus, the comparison is now possible between the full methods, of which the feature extraction is a part, enabling us to compare with the values reported by the authors.

### Web-based application interface implementation

The SYNPRED prediction models were implemented in a web-based application at http://www.moreiralab.com/resources/synpred/. The website's plots and front-end were constructed with plotly [66] and Flask [67], both freely available Python packages, on a framework that uses an in-house adaptation of Javascript, CSS, and HTML scripts. All the back-end hosting was mediated with Flask [67].

## Results and Discussion

### Measuring feature importance for model development

To understand the importance of each group of included features for the final model performance and to attain a more interpretable model, we analyzed each of the individual models with permutation importance. We perceived that more complex models, particularly DL-based models with different architectures, tend to make more extensive use of the omics-based features to over 70% of the total feature contribution (Figs. W9–W12 of the SYNPRED webserver). Contrarily, simpler models, such as kNN and SGD, made almost exclusive use of the drug features (above 90%) (Figs. W16 and W18 of the SYNPRED webserver). Other non-DL-based models made variable (between 20% and 80%) usage of the omics features (Figs. W13–W15 and W17 of the SYNPRED webserver). This observation highlights the importance of DL models to take full advantage of omics data for capturing the complexity of each cancer profile, thus improving drug pair–cell line combinations predictions. The advantages of using these algorithms when dealing with multidimensional omics data, particularly the great flexibility of DL architectures, were also previously emphasized [68].

We then looked for a possible biological relevance of the top 5 genes in each group of the most critical multiomics features to understand if genes contributing more to the prediction models were also implicated in tumorigenesis. Of the 15 ranked genes from expression, methylation, and CNV variations, all of them are used as prognostic cancer markers or have a role in tumor progression and treatment (Table 3). These data suggest that our models, especially DNNs, are likely to capture the most relevant information for each group of multiomics features for synergistic drug combinations. The remaining ranked genes organized by each ML model's best-contributing features are presented in interactive Sankey diagrams on the website landing page (Figs. W9–W18).

**Table 3:** Permutation importance of the top 5 proteins associated with expression, methylation, and CNV features as well as their associated biological relevance

Type of feature	Protein name	Protein description	Biological relevance^a^
**Expression**	TMSB4X	Thymosin beta-4 X-linked	Prognostic marker in renal cancer (unfavorable)
	MTCO2	Mitochondrially encoded cytochrome c oxidase II	Prognostic marker in liver cancer (favorable) and pancreatic cancer (favorable)
	MT-RNR2	Mitochondrially encoded 16S rRNA	Associated with survival outcomes in patients with cancer [69]
	MT-CO3	Mitochondrially encoded cytochrome c oxidase III	Prognostic marker in pancreatic cancer (favorable) and liver cancer (favorable)
	COX6C	Cytochrome c oxidase subunit 6C	Associated with breast cancer, thyroid tumors, uterine cancer, prostate cancer, and esophageal cancer [70], although not reported as prognostic
**Methylation**	C11ORF52	Chromosome 11 open reading frame 52	Associated with lung cancer [71], although not reported as prognostic
	NPY1R	Neuropeptide Y receptor Y1	Prognostic marker in breast cancer (favorable)
	TMBIM6	Transmembrane BAX inhibitor motif containing 6	Prognostic marker in renal cancer (favorable), head and neck cancer (unfavorable), and breast cancer (unfavorable)
	C2CD4D	C2 calcium-dependent domain containing 4D	C2CD4D-AS1 overexpression contributes to the malignant phenotype of lung adenocarcinoma cells [72], although not reported as prognostic
	EDNRB	Endothelin receptor type B	Prognostic marker in renal cancer (favorable)
**CNV**	UTY	Ubiquitously transcribed tetratricopeptide repeat containing, Y-linked	Associated with cutaneous melanoma, bladder urothelial carcinoma, B-cell lymphoma, small cell lung cancer, oligodendroglioma, chondroblastic osteosarcoma, and cutaneous melanoma [73, 74], although not reported as prognostic
	MACROD2	Mono-ADP ribosylhydrolase 2	Associated with growth of intestinal tumors [75], although not reported as prognostic
	WWOX	WW domain containing oxidoreductase	Prognostic marker in renal cancer (favorable) and breast cancer (unfavorable)
	DAZ2	Deleted in azoospermia 2	Associated with oligozoospermia [76], which is, in turn, highly associated with testicular cancer [77], although not reported as prognostic
	KANK1	KN motif and ankyrin repeat domains 1	Upregulating Kank1 gene inhibits human gastric and lung cancer progress [78, 79], although not reported as prognostic

a The protein description and biological importance were retrieved from the Human Proteins Atlas (https://www.proteinatlas.org/) and the Human Gene Database (https://www.genecards.org/). When this information was not listed in these databases, we presented the study that supports the biological relevance. Favorable and unfavorable are related to gene/protein contribution for cancer progression.

### Tuning and choosing the best ML parameters

An appropriate choice of the best model parameters should always be performed, as ML performance and training time are deeply affected by them. With that in mind, we used a grid search approach to test a comprehensive array of parameters and dataset combinations, including parameters for several ML methods, a comprehensive set of DL configurations, and preprocessing setups, as described above. Regarding the preprocessing datasets, autoencoder datasets performed worse in the training sets and slightly worse for the test set. These results led us to discard them as there was no benefit to the increased training time caused by the significantly higher dimensionality. We proceed with the dataset in which PCA was used for dimensionality reduction and replacing the missing values with 0, as these approaches performed better for most grid search runs [80, 81] (Supplementary Table S3).

### SYNPRED models for drug combination prediction

After selecting the best parameters for both DL with Keras and ML with scikit-learn, we trained models with the full training set according to the parameters in the best grid search performing metrics. The best individual models were used to attain each sample prediction to make the final ensemble for the 5-synergy reference model plus the full agreement. The final models were then evaluated in the test set and 3 different scenarios: leave cell out, leave drugs out, and leave drug combinations out, by attaining different classification (Supplementary Table S7) or regression (Supplementary Tables S8–S12) evaluation metrics.

*Classification model performance*. Prior to ensemble development, the best independent performing model was XGBoost with the following parameters: alpha = 0.25, max_depth = 6, n_estimators = 100. After ensemble, our final full-agreement SYNPRED comprised 4 DL-based and 6 ML-based models, attained with a DL architecture with 3 hidden layers of size 100 and a dropout rate of 0.60. When applied in an independent test set, our ensemble model displayed better performance (accuracy = 0.85, precision = 0.91, recall = 0.90, AUROC = 0.80, and F1-score = 0.90) than any other classic ML or DL models, including reference ones such as SVM, RF, or XGBoost frequently used for synergy prediction classification tasks (Table 4, Supplementary Table S7) [13,14, 82]. In the 3 independent scenarios, the full-agreement ensemble SYNPRED achieved higher precision values by returning the most relevant results than any other of the individual models. However, we saw a significant drop in the leave cells, drugs, and drug combinations out datasets.

**Table 4:** Best results obtained for the classification ensemble model

Subset used for evaluation^a^	Accuracy	Precision	Recall	AUROC	F1 score
Test	0.85	0.91	0.90	0.80	0.90
Leave cells out	0.37	0.89	0.13	0.55	0.22
Leave drugs out	0.33	0.86	0.13	0.53	0.22
Leave drug combinations out	0.24	1.00	0.21	0.61	0.35

a The final model had a dropout rate of [0.4] and an architecture of [10, 10, 10].

*Regression model performance*. Concerning the 5 regression tasks (Table 5), CSS (Supplementary Table S12) stands out—in either the metrics or the datasets considered—while the remaining 4 (ZIP, HSA, Bliss, and Loewe) (Supplementary Tables S8–S11) followed closely behind. Although in agreement with the presented data, this is unexpected considering the literature on the subject, which mainly uses Loewe. Indeed, historically, Loewe has been systematically chosen as the target regression reference model [14, 16–18, 20, 21]. For most cases in which this happens, there is no comparison with the remaining reference models. The few available comparative studies are mainly done outside the synergy prediction spectrum and somewhat under the scope of analyzing provided drug combination dose–response matrix data [33, 83]. By deploying an unbiased data-driven selection of the model, SYNPRED empirically assesses how realistically viable is the representation of 5 of the most common synergy reference models against a real biological dataset. Zagidullin et al. [29] have already pointed to the value of such agglomerative approaches.

**Table 5:** Best results obtained for the regression ensemble models, considering the test dataset

Synergy reference model	RMSE	MSE	Pearson	MAE	Spearman
CSS	11.07	122.61	0.86	7.43	0.87
Loewe	10.58	111.92	0.71	6.49	0.68
Bliss	4.35	18.92	0.71	3.07	0.59
HSA	4.09	16.70	0.73	2.86	0.64
ZIP	3.86	14.87	0.70	2.74	0.66

**Figure 3:**
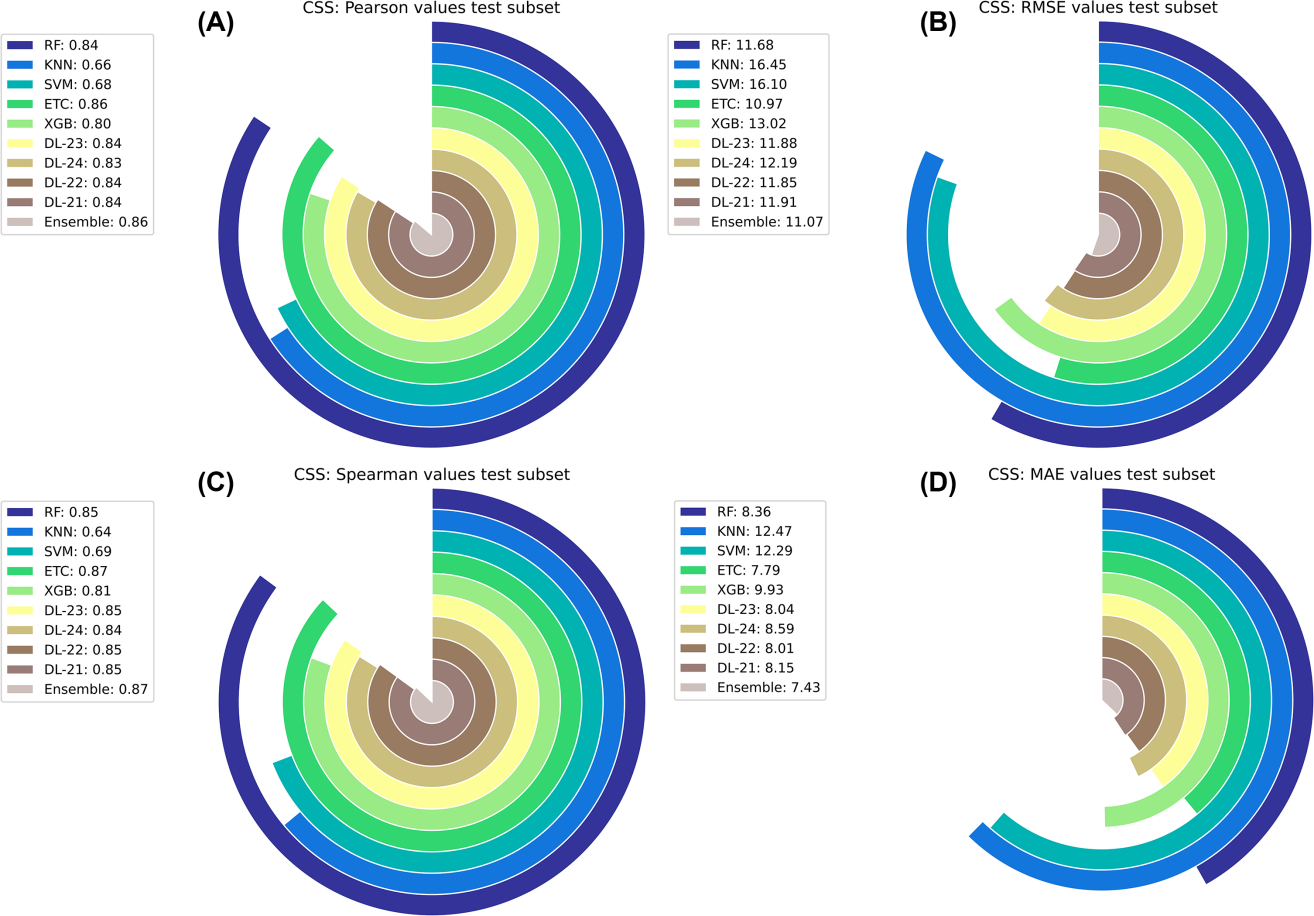
Circular bar plot representing the model's evaluation metrics for the CSS synergy reference model. (A) Model performance Pearson values evaluated in the test dataset. (B) Model performance RMSE values evaluated in the test dataset. (C) Model performance Spearman values evaluated in the test dataset. (D) Model performance MAE values evaluated in test dataset.

**Figure 4:**
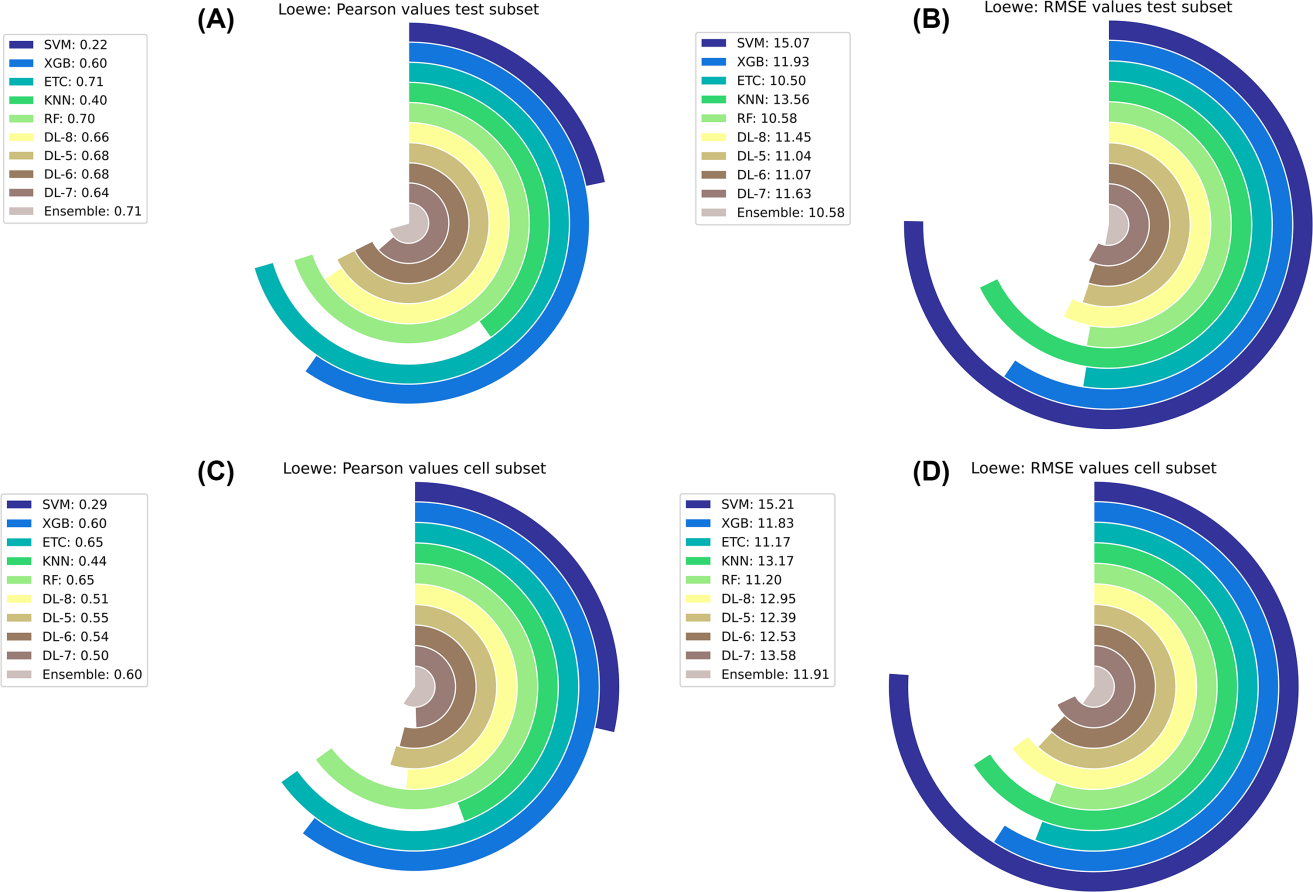
Circular bar plot representing the model's evaluation metrics for the Loewe synergy reference model. (A) Model performance Pearson values evaluated in the test dataset. (B) Model performance RMSE values evaluated in the test dataset. (C) Model performance Pearson values evaluated in the leave cells out dataset. (D) Model performance RMSE values evaluated in the leave cells out dataset.

The results of our best final ensemble regression model (CSS) outperformed all the individual predictors when evaluated in the test dataset and leave drug combinations out scenario, one of the most challenging ones (Table 5). Regarding correlation metrics and comparing to the literature standards [ 84], CSS achieved strong Pearson values (0.86 on the test and 0.74 on the leave cells out dataset). Concerning scale-depending performance metrics, the CSS had 11.07 and 13.63 RMSE on the test and leave cells out datasets, respectively. Considering that CSS values range within [−54.05, 99.84], our predictor was able to determine CSS synergy values with low error (Fig. 3). A similar pattern was exhibited by the Loewe ensemble predictor (Fig. 4).

### Benchmark

We benchmarked our pipeline following a multistep approach as described in the Methods section:

- Comparison of the best-performing individual DL and ML algorithms with the ensemble approaches for each prediction task—Supplementary Tables S7 to S12
- DeepSynergy [18] architecture implementation and comparison using our independent test set and validation sets—Supplementary Table S13
- Comparison with published methods for synergy calculations as reviewed by Kumar and Dogra [65]—Supplementary Table S14
- Comparison of our regression approaches to Matchmakers’ algorithm [20], DeepSynergy [18], and TreeCombo [12]—Supplementary Tables S15 and S16

Regarding (i), ensemble/aggregation of algorithms consistently outperforms or stands very close to the best individual predictors. XGBoost and extreme randomized trees were typically the second-best predictors. These results showcase how SynPred leverages previous information on algorithms such as TreeCombo [12] (which uses an individual XGBoost algorithm) or DeepSynergy [18], which is, in essence, the literature parent of several of the neural networks with conic architecture we used. In fact, in (ii) (Supplementary Table S13), it can be seen that the DeepSynergy [18] implementation on SynPred's pipeline behaves similarly to other DNN approaches in SynPred. These are good performers but unable to beat the ensemble algorithms.

When comparing the reported performance for algorithms in their own settings (iii), as reviewed by Kumar and Dogra [65], once again we need to take into account a very broad array of circumstances, such as algorithms, datasets (filtered or postprocessed), and synergy reference models (Supplementary Table S14). The high possible combination of factors that leads to the final methods’ performance is huge, and therefore this comparison has to be conducted with a limited few.

For instance, SynPred's highest performer predictor is the CSS predictor. However, it is impossible to justly compare our results to predictors that only focus on the Loewe synergy reference model. However, when considering the most recurring synergy reference model (Loewe), although SynPred shows lower Pearson and Spearman correlations, it also presents much lower errors (RMSE and MSE) compared to the best remaining algorithms. All these results highlight the need to consider different synergy reference models, which although not used before, were already suggested to be a valuable approach [29].

Finally (iv), we conducted closer comparisons (although still not optimal) with performances presented in Supplementary Table S15 and Supplementary Table S16. Regarding Supplementary Table S15, SynPred was run against Matchmakers’ [20] processing of DrugComb [29]. Upon doing this, both CSS and Loewe predictors from SynPred stood very close to the performance of Matchmakers [20], which is remarkable since this was the dataset used by the authors [20] to train the model. When inspecting Supplementary Table S16, in which the predictors were deployed upon NCI ALMANAC [85] (the dataset used in this study), SynPred stands out in all the synergy reference models with Pearson and Spearman correlation performance increments between 30.51% and 42.37%, as well as between 36.36% and 56.36%, respectively. Although MSE metrics are particularly hard to compare between datasets and methods, significant improvements were also observed.

### Web-based application description

**Figure 5:**
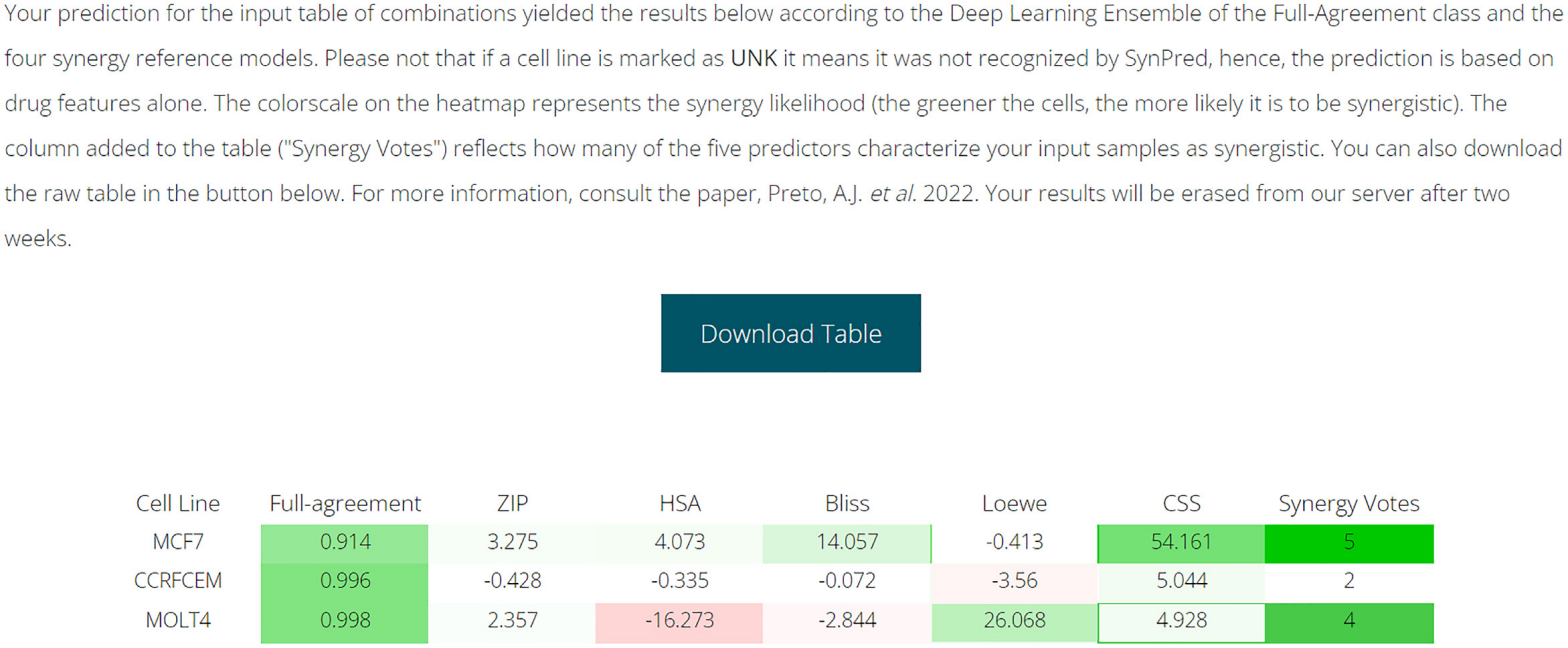
Example of the SYNPRED output prediction. Green colored cells represent a synergistic prediction, while red colored cells represent the nonsynergistic ones.

The classification and regression models for predicting the type of combinatory effect in drug pair–cell line samples are available as a web-based application at http://www.moreiralab.com/resources/synpred/. All the 11 described single models are deployed on user submission, as well as the ensemble approach. The user needs to submit 2 drugs as input in the *.smile format and selects from a drop-down menu, the primary body site corresponding to the tested cancer cell lines. The drugs are then subject to feature extraction by Mordred and a standard preprocessing (feature elimination and normalization) as thoroughly described in the Methods section. The output, displayed in a downloadable heatmap, is the drug combination prediction effect for each of the individual cell lines calculated with the ensemble classification and regression models and using 5 synergy reference models (ZIP, HSA, Bliss, Loewe, CSS) plus the full-agreement metric. Furthermore, the final tally of synergistic queries predicted by all models based on the prediction values is also displayed in the last column (“Synergy Votes”). This additional option facilitates the visualization of the type of combinatory effect between the 2 drugs and aims at strengthening the value of the prediction due to the lack of consensus between the different synergy reference models. The results are returned to the provided e-mail and displayed on the submission webpage (as shown in Fig. 5). Additionally, users can assess, explore, and visualize through different plots as well as export a summary of the synergy scores (calculated using ZIP, Bliss, HSA, Loewe, and CSS synergy reference models) by cell line used to develop the original dataset of SYNPRED. To our knowledge, this is the first webserver that can predict new drug synergy combinations without the need of uploading a partial or full dose–response matrix. This feature is an advantage compared with other models implemented in webservers that need these types of data for drug combination response prediction [ 28, 29].

## Conclusions

Synergistic anticancer drug combinations are a powerful tool to help tackle cancer drug resistance since they can simultaneously target multiple key molecules or pathways. The rational design of combination therapies is warranted to improve the efficacy, although this is a well-known time-consuming and expensive task. In recent years, ML algorithms' applicability for drug repurposing or novel drug design has been essential to demonstrate the importance of *in silico* methodologies to help overcome this problem. Some classification [13, 14, 17] and regression [16, 18–22] models using ML and omics data for predicting drug synergy combinations were already developed. However, the fittingness of the previously developed algorithms is sometimes hindered by using a single reference model (e.g., Bliss, Loewe, HSA, ZIP, or CSS) or by the difficulty in applying these models to new unseen data, since these are not straightforward to implement and require advanced bioinformatics skills. Our study leads to an innovative approach by highlighting the importance of choosing an appropriate synergy reference model, and explores how this choice influences the final predictor performance. Given the different sensitivity observed between these reference models in evaluating the degree of combination, a more comprehensive and rigorous approach that leverages all metrics to predict drug synergy is an asset.

This study introduced a new synergy prediction model, SYNPRED, that combines comprehensive multiomics data of cancer cell lines with physicochemical and structural features of drugs. This work is one of the first that takes 5 different synergy reference models (Bliss, HSA, Loewe, ZIP, and CSS) and uses one of the most comprehensive and balanced databases regarding the synergistic–nonsynergistic distribution, the NCI ALMANAC. Our top-ranked classification and regression models, an ensemble developed with the best machine learning models, achieved state-of-the-art performance to predict synergistic drug combinations in an independent dataset. The best-performing prediction model in SYNPRED is, undoubtedly, CSS (RMSE, 11.07; MSE, 122.61; Pearson, 0.86; MAE, 7.43; Spearman, 0.87). However, we advise the users to considers the aggregate of results, albeit with a higher focus on CSS. We included a “Voting classifier” output that tallies the results of the 6 predictors to aid the user's interpretation of the results. If more than 5 predictors yield a positive result, the submission sample is likely to be synergistic, while if it is only 1 or lower, it is likely to be nonsynergistic. Besides, we provide the complete workflow for a standalone deployment in our GitHub coupled with a freely available and easy-to-use webserver (http://www.moreiralab.com/resources/synpred/) that requires only 2 drugs’ SMILEs as inputs, thus alleviating the need for uploading a conventional and laborious dose–response matrix. SYNPRED can be a valuable tool to the scientific and medical community for drug repurposing or *in silico* discovery of new anticancer drug combinations.

Additionally, given the importance of multiomics data in cell line classification and therapy response, we combined all the available multiomics features in the CCLE database to explore their contribution to model development. The knowledge mined from this analysis demonstrates the capacity of different ML models to deal with multiomics data, with DL algorithms being much more able to learn and leverage this complex type of features. We found that the most ranked proteins in each of the most contributing multiomics features are important cancer biomarkers or have a role in tumorigenesis, demonstrating DNN models' capacity to capture their significance and use this information for the final model development. In the future, we expect to include protein–protein interactions data and network analysis to improve the model performance, aiming to identify drug combinations with potential new targets across different cell lines.

## Availability of Supporting Source Code and Requirements

Project name: SYNPRED

Project homepage: https://github.com/MoreiraLAB/synpred

Operating system(s): Linux, Mac OS X, Windows

Programming language: Python and R

Other requirements: Python 3.8.2 or higher, R 3.6.3 or higher

License: GPL-3.0

Biotools: Synpred

RRID: SCR_022693

## Data Availability

SYNPRED is a free, open-source, web-based application available at http://www.moreiralab.com/resources/synpred/ without any login or registration requirements. The source code of the web-based application implementation is deposited in the GitHub repository (https://github.com/MoreiraLAB/synpred) to allow the stand-alone use of the application and further integration and comparison with other models. The code is fully developed in Python and R languages; hence, it can be deployed fully without charge. The multiomics data included in this study are available at the corresponding references mentioned in the main text. Supporting data and an archival copy of the code are also available via the GigaScience database GigaDB [89].

## Additional Files

**Supplementary Table S1**. Conditions for dimensionality reduction with autoencoders. Hidden and bottleneck layers definition according to the number of features.

**Supplementary Table S2**. Conditions for dimensionality reduction with autoencoders. Number of epochs of the autoencoder training according to either the number of samples or number of features.

**Supplementary Table S3**. Final datasets to be subjected to training.

**Supplementary Table S4**. Grid search combination parameters using 5% on the training set with deep learning algorithms.

**Supplementary Table S5**. Grid search combination parameters using 5% on the training set with non–deep learning algorithms.

**Supplementary Table S6**. Grid search combination parameters of the ensemble neural network.

**Supplementary Table S7**. Final metrics of the classification models evaluated in an independent test set and 3 different scenarios (leave cell out, leave drugs out, and leave drug combinations out) using full-agreement synergy values.

**Supplementary Table S8**. Final metrics of the regression models evaluated in an independent test set and 3 different scenarios (leave cell out, leave drugs out, and leave drug combinations out) using the Bliss synergy reference model.

**Supplementary Table S9**. Final metrics of the regression models evaluated in an independent test set and 3 different scenarios (leave cell out, leave drugs out, and leave drug combinations out) using the HSA synergy reference model.

**Supplementary Table S10**. Final metrics of the regression models evaluated in an independent test set and 3 different scenarios (leave cell out, leave drugs out, and leave drug combinations out) using the Loewe synergy reference model.

**Supplementary Table S11**. Final metrics of the regression models evaluated in an independent test set and 3 different scenarios (leave cell out, leave drugs out, and leave drug combinations out) using the ZIP synergy reference model.

**Supplementary Table S12**. Final metrics of the regression models evaluated in an independent test set and 3 different scenarios (leave cell out, leave drugs out, and leave drug combinations out) using the CSS synergy reference model.

**Supplementary Table S13**. DeepSynergy [86] reimplementation on the dataset that yielded the best results for SynPred (with PCA preprocessing and missing values replacement with 0), against the synergy reference model the original work targeted—Loewe.

**Supplementary Table S14**. Comparison of final metrics of the classification and regression models of SynPred to the methods reviewed by Kumar and Dogra [65].

**Supplementary Table S15**. Comparison of the performance of SynPred and other recent algorithms, according to their respective reporting metrics upon deployment in DrugCombo [87].

**Supplementary Table S16**. Comparison of the performance of SynPred and other recent algorithms, according to their respective reporting metrics upon deployment in NCI ALMANAC [88].

giac087_GIGA-D-21-00416_Original_Submission

giac087_GIGA-D-21-00416_Revision_1

giac087_GIGA-D-21-00416_Revision_2

giac087_Response_to_Reviewer_Comments_Original_Submission

giac087_Response_to_Reviewer_Comments_Revision_1

giac087_Reviewer_1_Report_Original_Submission A. ErcÃ¼ment Ã‡iÃ§ek -- 1/16/2022 Reviewed

giac087_Reviewer_1_Report_Revision_1 A. ErcÃ¼ment Ã‡iÃ§ek -- 7/7/2022 Reviewed

giac087_Reviewer_2_Report_Original_Submission Remzi Celebi -- 1/27/2022 Reviewed

giac087_Supplemental_File

## Abbreviations

ACC: accuracy; AI: artificial intelligence; ANN: artificial neural network; AUROC: area under the receiver operating curve; CCLE: Cancer Cell Line Encyclopaedia; CNV: copy number variation; DL: deep learning; DNN: deep neural network; ENS: ensemble; ETC: extreme randomized trees; F1: F1-score; GPU: graphics processing unit; HL: hidden layer; HSA: highest single agent; kNN: k-nearest neighbor; MAE: mean absolute error; miRNA: microRNA; ML: machine learning; MLP: multilayer perceptron; MSE: mean square error; PC: principal component; PCA: principal component analysis; PREC: precision; REC: recall; ReLU: rectified linear unit; RF: random forest; RMSE: root mean square deviation; SGD: stochastic gradient descent; SMILE: simplified molecular-input line-entry System; SVM: support vector machine; SYNPRED: SYNergy PREDiction; XGBoost: extreme gradient boosting; ZIP: zero interaction potency.

## Funding

This work was supported by the European Regional Development Fund through the COMPETE 2020–Operational Programme for Competitiveness and Internationalisation and Portuguese national funds via Fundação para a Ciência e a Tecnologia (FCT) (LA/P/0058/2020, UIDB/04539/2020, UIDP/04539/2020, POCI-01–0145-FEDER-031356, and DSAIPA/DS/0118/2020). FCT also supported A.J.P. with a PhD scholarship (SFRH/BD/144966/2019). J.M. is supported by a Individual Junior Postdoctoral Research Contract from FCT Scientific Employment Stimulus (2021.03416.CEECIND). Funding for open-access charge: Fundação para a Ciência e a Tecnologia (DSAIPA/DS/0118/2020).

## Competing Interests

The authors declare that they have no competing interests.

## Authors' contributions

A.J.P., methodology; software; validation; formal analysis; investigation; resources; writing—review & editing; visualization. P.M.-F., methodology; software; investigation; resources; data curation; writing—original draft preparation. J.M., conceptualization; methodology; formal analysis; data curation; writing—original draft preparation; writing—review & editing; supervision; project administration. I.S.M., conceptualization; writing—review & editing; visualization; supervision; project administration; funding acquisition.
