# Supplementary material for: SYNPRED: prediction of drug combination effects in cancer using different synergy metrics and ensemble learning
Source: Gigascience. 2022 Sep 26;11:giac087. doi: 10.1093/gigascience/giac087 (PMC9511701; doi:10.1093/gigascience/giac087)
Supplement: giac087_GIGA-D-21-00416_Original_Submission [file giac087_giga-d-21-00416_original_submission.pdf]

GigaScience

SYNPRED: Prediction of Drug Combination Effects in Cancer using Different Synergy Metrics and Ensemble Learning

--Manuscript Draft--

**Manuscript Number:**	GIGA-D-21-00416
**Full Title:**	SYNPRED: Prediction of Drug Combination Effects in Cancer using Different Synergy Metrics and Ensemble Learning
**Article Type:**	Research
**Funding Information:**	Fundação para a Ciência e a Tecnologia (LA/P/0058/2020) — Prof. Irina Sousa Moreira<br>Fundação para a Ciência e a Tecnologia (POCI-01-0145-FEDER-031356) — Prof. Irina Sousa Moreira<br>Fundação para a Ciência e a Tecnologia (DSAIPA/DS/0118/2020) — Prof. Irina Sousa Moreira<br>Fundação para a Ciência e a Tecnologia (SFRH/BD/144966/2019) — Mr António J. Preto
**Abstract:**	Background<br><br>In cancer research, high-throughput screening technologies produce large amounts of multiomics data from different populations and cell types. However, analysis of such data encounters difficulties due to disease heterogeneity, further exacerbated by human biological complexity and genomic variability. The specific profile of cancer as a disease (or, more realistically, a set of diseases) urges the development of approaches that maximize the effect while minimizing the dosage of drugs. Now is the time to redefine the approach to drug discovery, bringing an Artificial Intelligence (AI)-powered informational view that integrates the relevant scientific fields and explores new territories.<br><br>Results<br><br>Here, we show SYNPRED, an interdisciplinary approach that leverages specifically designed ensembles of AI algorithms, links omics and biophysical traits to predict anticancer drug synergy. It uses four reference models (Bliss, Highest Single Agent, Loewe, and Zero Interaction Potency), which, coupled with AI algorithms, allowed us to attain the ones with the best predictive performance and pinpoint the most appropriate reference model for synergy prediction, often overlooked in similar studies. By using an independent test set, SYNPRED exhibits state-of-the-art performance metrics either in the classification (accuracy – 0.80, precision – 0.81, recall – 0.81, AUROC – 0.80, and F1-score - 0.81) or in the regression models, mainly when using the Zero Interaction Potency synergy reference model (RMSE – 7.10, MSE – 50.46, Pearson – 0.80, R 2 – 0.43, MAE – 4.61, Spearman – 0.73). Moreover, data interpretability was achieved by deploying the most current and robust feature importance approaches. A simple web-based application was constructed, allowing easy access by non-expert researchers.<br><br>Conclusions<br><br>The performance of SYNPRED rivals that of the existing methods that tackle the same problem, yielding unbiased results trained with one of the most comprehensive datasets available (NCI-ALMANAC). The leveraging of different reference models allowed deeper insights into which of them is the most appropriately one to use for synergy prediction. The Zero Interaction Potency clearly stood out with improved performance among the full scope of surveyed approaches and synergy reference models. Furthermore, SYNPRED takes a particular focus on data interpretability, which has been in the spotlight lately when using the most advanced AI techniques.
**Corresponding Author:**	Irina Sousa Moreira<br>University of Coimbra: Universidade de Coimbra<br>Coimbra, US and Canada only PORTUGAL
**Corresponding Author Secondary**	

Powered by Editorial Manager® and ProduXion Manager® from Aries Systems Corporation

Information:	
Corresponding Author's Institution:	University of Coimbra: Universidade de Coimbra
Corresponding Author's Secondary Institution:	
First Author:	António J. Preto
First Author Secondary Information:	
Order of Authors:	António J. Preto
	Pedro Matos-Filipe
	Joana Mourão
	Irina Sousa Moreira
Order of Authors Secondary Information:	
Additional Information:	
**Question**	**Response**
Are you submitting this manuscript to a special series or article collection?	No
**Experimental design and statistics**<br><br>Full details of the experimental design and statistical methods used should be given in the Methods section, as detailed in our Minimum Standards Reporting Checklist. Information essential to interpreting the data presented should be made available in the figure legends.<br><br>Have you included all the information requested in your manuscript?	Yes
**Resources**<br><br>A description of all resources used, including antibodies, cell lines, animals and software tools, with enough information to allow them to be uniquely identified, should be included in the Methods section. Authors are strongly encouraged to cite Research Resource Identifiers (RRIDs) for antibodies, model organisms and tools, where possible.<br><br>Have you included the information requested as detailed in our Minimum Standards Reporting Checklist?	Yes

**Availability of data and materials**<br><br>All datasets and code on which the conclusions of the paper rely must be either included in your submission or deposited in publicly available repositories (where available and ethically appropriate), referencing such data using a unique identifier in the references and in the "Availability of Data and Materials" section of your manuscript.<br><br>Have you have met the above requirement as detailed in our Minimum Standards Reporting Checklist?	Yes

Manuscript

Click here to access/download;Manuscript;Synpred_GIGA-D-21-00121.docx

SYNPRED: Prediction of Drug Combination Effects in Cancer using Different Synergy Metrics and Ensemble Learning

António J. Preto[1,2], Pedro Matos-Filipe[1], Joana Mourão[1] and Irina S. Moreira[3,1*]

[1]University of Coimbra, Center for Neuroscience and Cell Biology, 3004-504 Coimbra, Portugal

[2]PhD Programme in Experimental Biology and Biomedicine, Institute for Interdisciplinary Research (IIIUC), University of Coimbra, Casa Costa Alemão, 3030-789 Coimbra, Portugal

[3]University of Coimbra, Department of Life Sciences, Calçada Martim de Freitas, 3000-456 Coimbra, Portugal

* To whom correspondence should be addressed.

Tel: (+351) 239 240 227

Email: irina.moreira@cnc.uc.pt

ABSTRACT

Background: In cancer research, high-throughput screening technologies produce large amounts of multiomics data from different populations and cell types. However, analysis of such data encounters difficulties due to disease heterogeneity, further exacerbated by human biological complexity and genomic variability. The specific profile of cancer as a disease (or, more realistically, a set of diseases) urges the development of approaches that maximize the effect while minimizing the dosage of drugs. Now is the time to redefine the approach to drug discovery, bringing an Artificial Intelligence (AI)-powered informational view that integrates the relevant scientific fields and explores new territories.

Results: Here, we show SYNPRED, an interdisciplinary approach that leverages specifically designed ensembles of AI algorithms, links omics and biophysical traits to predict anticancer drug synergy. It uses four reference models (Bliss, Highest Single Agent, Loewe, and Zero Interaction Potency), which, coupled with AI algorithms, allowed us to attain the ones with the best predictive performance and pinpoint the most appropriate reference model for synergy prediction, often overlooked in similar studies. By using an independent test set, SYNPRED exhibits state-of-the-art performance metrics either in the classification (accuracy – 0.80, precision – 0.81, recall – 0.81, AUROC – 0.80, and F1-score - 0.81) or in the regression models, mainly when using the Zero Interaction Potency synergy reference model (RMSE – 7.10, MSE – 50.46, Pearson – 0.80, R^2 – 0.43, MAE – 4.61, Spearman – 0.73). Moreover, data interpretability was achieved by deploying the most current and robust feature importance approaches. A simple web-based application was constructed, allowing easy access by non-expert researchers.

Conclusions: The performance of SYNPRED rivals that of the existing methods that tackle the same problem, yielding unbiased results trained with one of the most comprehensive datasets available (NCI-ALMANAC). The leveraging of different reference models allowed deeper insights into which of them is the most appropriately one to use for synergy prediction. The Zero Interaction Potency clearly stood out with improved performance among the full scope of surveyed approaches and synergy reference models. Furthermore, SYNPRED takes a particular focus on data interpretability, which has been in the spotlight lately when using the most advanced AI techniques.

KEYWORDS

Ensemble-Learning; Interpretability; Omics; Biophysics; Drug Synergy; Cancer.

BACKGROUND

Cancer, a heterogeneous group of diseases, is one of the leading causes of mortality and the most significant barrier to increasing life expectancy worldwide. The International Agency for Research on Cancer estimates that, by 2040, approximately 29.5 million new cancer cases and 16.4 million deaths will be reported, mainly due to the population's growth and ageing [1]. One of the significant contributors to this disease's global burden is the development of therapy resistance and, consequently, tumour relapse. Drug resistance in cancer is a multifactorial problem driven by the tumour microenvironment and genetic and nongenetic/epigenetic mechanisms that, along with cell plasticity, contribute to tumour heterogeneity [2]. In clinical settings, this problem is minimized with a combination of drugs administered together or in sequence, i.e., polytherapy. Targeting multiple components of different or interconnected cancer pathways is an efficient strategy to block vital biological processes [3].

Drug combinations with a synergistic effect, i.e., when the total therapeutic effect of both drugs is greater than the expected additive monotherapy effect [4], were successfully developed and applied in the treatment of different types of tumours, such as human epidermal growth factor receptor 2-positive breast cancer [5], chronic myeloid leukaemia [6], prostate cancer [7] or BRAF-mutant melanoma [8]. Nevertheless, this simultaneous administration can also result in a reduced therapeutic effect and possible toxicity (designated antagonism) or in the same beneficial effect when compared with the expected additive monotherapy effect (additivity) [4]. The experimental identification of successful synergistically effective combinations is a well-known time-consuming, and expensive task. Therefore, there is still a significant need for efficient and user-friendly computational methods, available in easy to use interfaces, to complement and speed-up the traditional approaches by predicting the best synergistic drug combinations [9,10].

In the last years, the development and improvement of high-throughput technologies and computational tools boosted the use of large volumes of multi-omics data (e.g., genomic, transcriptomic, proteomic) essential to dissect and uncover the complex molecular signatures of cancer. Machine Learning (ML)

algorithms have attracted particular attention for their ability to learn new associations and extract valuable insights from this type of data. A few ML models based on eXtreme Gradient Boosting, Random Forest, Elastic Nets, Support Vector Machine, and Naïve Bayes were already developed to predict the best combination of anticancer drugs by the integration of omics data with chemoinformatic properties of drugs or network information of their targets [11–14]. Likewise, Deep-Learning (DL) implemented via Deep-Neural Networks (DNNs) was particularly useful in dealing with the high multi-dimensionality of omics data in supervised and unsupervised contexts. DL classification and regression models such as AuDNNsynergy [15], DeepDDS [16], DeepSynergy [17], DeepSignalingSynergy [18], MatchMaker [19], TranSynergy [20] or the work by Xia and colleagues [21] were recently developed for drug combination prediction. Nearly all the surveyed works developed drug synergy prediction models based upon a single reference model, which was in most cases the Loewe reference model [13,15–17,19,20]. Currently, there is a wide scope of well-studied available reference models, including the Bliss independence [22], Highest Single Agent – HSA [23], Loewe additivity [24,25] and Zero Interaction Potency – ZIP [26]. As such, this led us to the question of whether the development of a novel prediction approach should be based solely upon a single reference model. Besides, most of the available web interfaces such as DECREASE [27] or DrugComb [28] require for synergy prediction the upload of a full or partial mandatory dose-response matrix (experimentally determined), which hinders its systematic use by the scientific community and handicaps its usefulness.

To overcome the current problems found in the field, we developed SYNPRED (SYNergy PREDiction), a collection of *in-silico* ensemble classification and regression models that considers several synergy reference models: Bliss, Loewe, HSA, and ZIP. It was developed by integrating multi-omics features of cell lines, phenotypic, and biophysical data, particularly physicochemical and structural features of drugs. SYNPRED displays a good predictive performance and inherently addresses the issue at a broader and more profound angle than the existing approaches, which generally focus on either classification or a single regression task and typically use a single synergy reference model. We made available the stand-alone deployment at https://github.com/MoreiraLAB/synpred, which allows the user the opportunity to undergo bulk prediction with SYNPRED. Additionally, for the first time, a user-friendly web-based application was assembled and made freely available online at http://www.moreiralab.com/resources/synpred/ to predict drug combinations, requiring only the upload of the two drugs Simplified Molecular-Input Line-Entry System (SMILEs) to be tested. This interactive

platform will allow users with different backgrounds, from scientists to clinicians, to test, reproduce and validate our models and data. The workflow used for the development of SYNPRED is depicted in **Figure 1**.

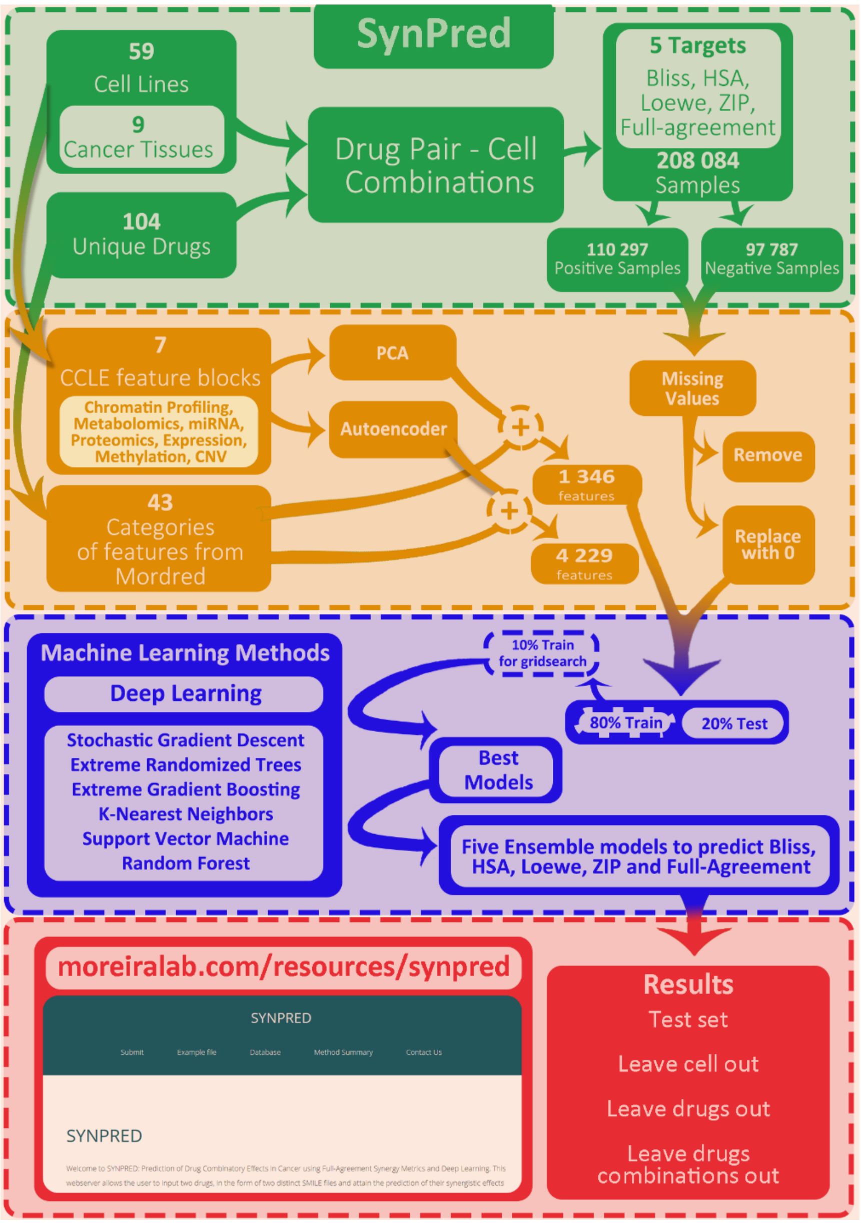

Figure 1. SYNPRED workflow summary. (Green) - Dataset construction. The National Cancer Institute - A Large Matrix of Anti-Neoplastic Agent Combinations database (phenotypic data) and the Cancer Cell Line Encyclopedia (CCLE) (multiomics data) were used for this purpose. Four reference models (Bliss, Highest Single Agent-HSA, Loewe, Zero Interaction Potency-ZIP) were used to quantify the combination degree and retrieve a full agreement between all metrics. (Orange) – Feature extraction and data pre-processing. Included normalization and dimensionality reduction using autoencoder or

Principal Component Analysis (PCA). (Blue) – Gridsearch and prediction models development using a training set. (Red) – Model evaluation using different classification and regression metrics in an independent test set and three different scenarios: i) leave cell out dataset, ii) leave drugs out dataset, iii) leave drug combinations out dataset.

DATA AND METHODS

Experimental drug combination phenotypic data. Drug combination phenotypic data was acquired via bulk-download from the largest-to-date dataset from National Cancer Institute - A Large Matrix of Anti-Neoplastic Agent Combinations (NCI-ALMANAC) through https://wiki.nci.nih.gov/display/NCIDTPdata/NCI-ALMANAC [29]. To this date, the dataset includes phenotypic data of tested cancer cell lines (growth percentage) of 104 unique FDA-approved drugs. These drugs were tested in combination against 59 cell lines from 9 cancer types currently included in the NCI [30,31], comprising a total of 311.466 drug pair/cell line combinations. Drug sensitivity assays included in NCI-ALMANAC were performed at the NCI's Frederick National Laboratory for Cancer Research, the Stanford Research Institute, and the University of Pittsburgh. Briefly, for each assay, cells were cultivated for 48 hours in a 3x3 or a 5x3 concentration matrix (different concentration values for each drug in combination) and the endpoint determined by Sulforhodamine B or CellTiter-Glo [29]. From these records, the authors retrieved the cell growth percentage at each drug concentration point, which corresponds to the percentage of growth of the cell lines in the presence of each combination, yielding a final viability assessment.

Combination scores and class definition. The phenotypic data from high-throughput drug combination screens were analysed using the "SynergyFinder" R package [32] (version 3.0.8). "SynergyFinder" leverages the percentage of cell growth included in the dataset to assess the degree of combination for each pair of drug concentrations by using several synergy reference models. We decided not to include the ComboScore reference model [29] since it was not clear from the literature how the synergy scores of different drug concentrations could be aggregated. As such, only the most well-studied synergy reference models described in the literature were included as they were the only that met the criteria of characterising the effects of a drug pair on a cell line with a final single synergy score. This approach narrowed down our options to the four most well-known synergy reference models: Bliss independence

(**Equation 1**) [22], Loewe additivity (**Equation 2**) [24,25], Highest Single Agent (HSA) (**Equation 3**) [23], and Zero Interaction Potency (ZIP) (**Equation 4**) [26].

$$yBliss = y1 + y2 - y1y2$$

Equation 1: Bliss independence model. yBliss – Bliss response; y1 – drug1 response; y2 – drug2 response.

$$yLoewe = \frac{Emin + Emax\left(\frac{x1 + x2}{m}\right)^{\lambda}}{1 + \left(\frac{x1 + x2}{m}\right)^{\lambda}}$$

Equation 2: Loewe additivity model. *yLoewe* – Loewe response; *Emin* – minimum drug response; *Emax* – maximum drug response; *m* – dose that produces midpoint effect between *Emin* and *Emax*; *λ* – shape parameter indicating the slope of the curve; *x1* – drug 1 dose; *x2* – drug 2 dose.

$$yHSA = \mathrm{ma\,x}(y1, y2)$$

Equation 3: Highest Single Agent (HSA) model; *yHSA* – HSA response; *y1* – drug 1 response; *y2* – drug 2 response.

$$yZIP = \frac{\left(\frac{x1}{m1}\right)^{\lambda 1}}{1 + \left(\frac{x1}{m1}\right)^{\lambda 1}} + \frac{\left(\frac{x2}{m2}\right)^{\lambda 2}}{1 + \left(\frac{x2}{m2}\right)^{\lambda 2}} - \left(\frac{\left(\frac{x1}{m1}\right)^{\lambda 1}}{1 + \left(\frac{x1}{m1}\right)^{\lambda 1}} * \frac{\left(\frac{x2}{m2}\right)^{\lambda 2}}{1 + \left(\frac{x2}{m2}\right)^{\lambda 2}}\right)$$

Equation 4: Zero Interaction Potency (ZIP) model; *yZIP* – ZIP response; *x1* – drug 1 dose; *x2* – drug 2 dose; *m1* – dose that produces midpoint effect for drug 1; *m2* – dose that produces midpoint effect for drug 2; *λ1* – shape parameter indicating the slope of the curve for drug 1; *λ2* – shape parameter indicating the slope of the curve for drug 2.

$$Y_i^{ApBq} = 100 * \frac{T_1^{ApBq} - T_0}{T_1^0 - T_0}$$

With this data, a binary classifier was first developed to identify the type of combinatory effect present in each drug pair-cell line sample, where the values above the threshold (0, as defined for each metric by SynergyFinder) (https://synergyfinder.fimm.fi/synergy/synfin_docs/) were defined as synergistic, and the remaining ones were classified as non-synergistic. The dataset used for training considered full-

agreement combination assessment, i.e., we only kept the instances on which combination classification was the same across the four previous reference classifiers. For the dataset used, this process yielded 110.287 synergistic samples and 97.787 non-synergistic samples. For the regression model deployment, we used the values attained directly from SynergyFinder calculation to each synergy reference model (Bliss, HSA, Loewe, ZIP). Except for Loewe ([-5037.44, 249.71]), all remaining synergy reference model values were in similar scales (ZIP = [-129.43,167.99], HSA = [-143.58,174.10], Bliss = [-130.31,170.81]). For Loewe, the maximum value was significantly higher than the remaining models, and the minimum was even more extremely distant from its counterparts in the other reference models (**Figure 2, Figures W1-W5** of the SYNPRED webserver).

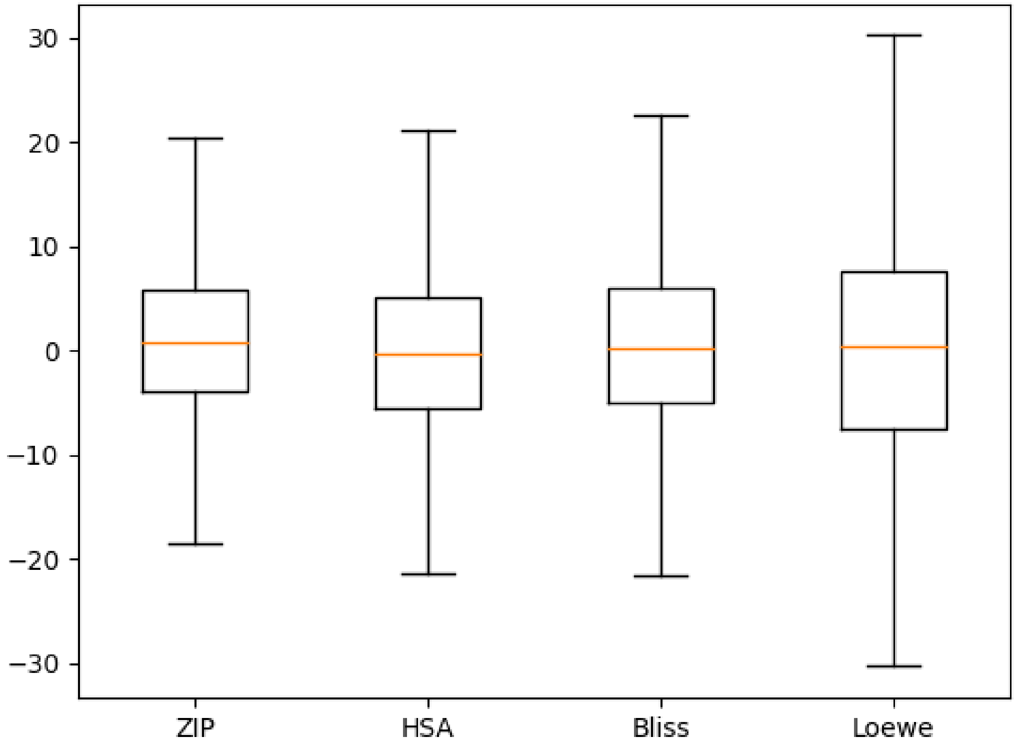

Figure 2. Box plot representing the distribution of synergy scores (y-axis) in respect to the four reference models: Bliss, HSA, Loewe, and ZIP (x-axis). The black boxes represent the difference between the upper 75% and the lower 25% quartiles (interquartile range); the horizontal orange line is the median; the whiskers are the lower and upper values that are not outliers or extremes (not represented as some of these values are off range).

Drug molecular descriptors. Each drug included in NCI-ALMANAC was analysed to extract its physicochemical and structural features. A SMILE representation of the drugs was acquired from PubChem [33]. SMILEs were then used to mine molecular descriptors using the Python package "Mordred" (version 1.1.2) [34]. In total, was retrieved an array of 1.613 numeric features of 43 different categories making a two-dimensional molecular description of the drugs. Feature-arrays comprising non-numerical attributes or displaying zero variance were deleted. This pre-processing left 586 features describing each drug included in NCI-ALMANAC, distributed across 28 categories (**Table 1**). The resulting features were subjected to normalisation by removing the mean and scaling to unit variance with scikit-learn's StandardScaler [35].

Table 1. Number of features according to the molecular descriptor category of Mordred. Features are categorized as Energetic (E), Pharmacological (P), Structural (S) or Miscellaneous (M - in case of evaluating characteristics of multiple fields).

**Number of Features per Descriptor Category**					
E	Acidity/Basicity	2	S	Information Content	36
P	ADME	3	S	Molecular Complexity	1
S	Aromatics	2	P	Molecular Operating Environment	51
S	Atom Count	16	S	Molecule Graph	5
S	Atom-bond Connectivity	2	S	Path Count	21
M	Autocorrelation	180	E	Polarizability	2
S	Bond Count	9	S	Ring Count	66
E	Atomic Orbitals	10	S	Rotatable Bonds	1
S	Chirality	38	S	Topological Charges	21
S	Constitutional	14	S	Topological Index	7
E	Energy State	68	S	Topological Polar Surface Area	2
S	Fragment Complexity	1	S	Walk Counts	21
S	Framework	1	S	Weight	2
S	Hydrogen Bonds	2	M	Wildman-Crippen	2

Omics data of cancer cell lines. Omics data (expression, copy number variation, and methylation, global chromatin profiling, metabolomics, microRNA, proteomic profiling) describing the cancer cell lines were acquired via bulk download from the Cancer Cell Line Encyclopedia - CCLE (https://sites.broadinstitute.org/ccle/) [36]. The number of cell lines included in CCLE varies depending on the type of omics data available at the time. Correspondence of cell line IDs between NCI-ALMANAC and CCLE was performed according to data available at the Swiss Institute of Bioinformatics Cellosaurus Website [37]. According to the affected tissue, annotations acquired through Cellossaurus split the CCLE cell lines into 21 different cancer types. In agreement with the original publications [36,38], expression data were obtained through RNA-sequencing and processed to obtain level expression in transcripts per million by the expectation-maximization algorithm (file: CCLE_RNAseq_rsem_genes_tpm_20180929.txt.gz). Copy Number Variation (CNV) data were acquired from the Affymetrix SNP6.0 Arrays (file: CCLE_copynumber_byGene_2013-12-03.txt.gz). Copy numbers were normalized by the most similar HapMap normal samples [39]. Segmentation of normalized log2 (CN/2) ratios was achieved using the circular binary segmentation algorithm [36,40]. Methylation data were derived by quantifying CpG islands using Reduced Representation Bisulfite Sequencing (file: CCLE_RRBS_tss_CpG_clusters_20181022.txt.gz). Global chromatin profiling was attained using multiple reaction monitoring for 42 combinations of histone marks (file: CCLE_GlobalChromatinProfiling_20181130.csv). Metabolomics data were acquired in parallel with global chromatin profiling by reporting the abundance measures of 225 metabolites (file: CCLE_metabolomics_20190502.csv). MicroRNA associated with cancer dependencies was correlated, regarding 734 microRNAs, with the Achilles gene dependency dataset. Protein profiling was measured with Reverse Phase Protein Arrays for 213 antibodies (file: CCLE_RPPA_20181003.csv) [38].

Dimensionality reduction of omics data. Data were normalized by removing the mean and scaling to unit variance with scikit-learn's StandardScaler [35]. Due to the omics data's high complexity, we performed dimensionality reduction to minimize the noise introduced in the dataset by highlighting the essential features. The datasets already described were used to build and train a Multi-Layer Perceptron (MLP) autoencoder, an unsupervised Artificial Neural Network (ANN) with a typical "hourglass" architecture, which is often used to perform dimensionality reduction in vast and high-dimensional datasets such as the ones observed with omics data [41–43]. This type of MLPs usually consists of three parts; an encoder that abstracts the input into hidden variables, i.e., a latent-space

representation, a bottleneck layer that holds the smallest Hidden Layer (HL) (for purposes of dimensionality reduction, this is the layer that defines the size of the reduced dataset), and a decoder that reconstructs the original input data from the hidden data [44,45]. Seven autoencoders, one for each of the CCLE feature blocks, were developed by using Keras with a TensorFlow for Graphics Processing Units (GPU) (Version 2.3.1) backend [46]. Each of the autoencoders comprised seven layers, of which five were HLs. The input and output layers follow the number of available features in all cell lines, as displayed in **Table 2**. The number of nodes within the bottleneck layer of each of the seven autoencoders (used for extraction of the encoded features) corresponds to the autoencoder's final number of features. The two HL in each of the encoder and decoder sections vary in size according to the number of samples and features available (**Table 1_Supplementary Material**). In this stage, all models used Adam [47] as an optimizer function with a learning rate of 0.001. Rectified Linear Unit (ReLU) activation function was used in all layers. Mean Square Error (MSE) was used as a loss function. The models were trained for 1000, 250, or 100 epochs, depending on the dataset size (**Table 2_Supplementary Material**). After training, each autoencoder's bottleneck layer was used to perform dimensionality reduction of the omics data according to **Table 2**.

PCA, a commonly used method for dimensionality reduction [48], was also applied in the same datasets as the autoencoder, for which 25 Principal Components (PCs) were defined. It means that, by using PCA, each of the datasets was transformed to yield only 25 features, totalling 175 features to describe each unique cell line. As shown in **Table 2**, each feature block from CCLE had its variance explained in a range from 0.89 to 0.99. Since the seven blocks were used simultaneously for each sample, each cell line is thoroughly described by the components extracted with the PCA. Missing values (in both autoencoder and PCA) were processed by either dropping the sample entirely or replacing the missing values by zero.

Table 2. Number of features pertaining the omics data and the corresponding amount for both the autoencoder and the PCA processing.

**Omics Data**	**Number of available cell lines**	**Number of available features**	**Number of features after autoencoder**	**Number of features after PCA**	**Explained variance (PCA)**
Expression	1019	57820	1156	25	0.89

Omics Data	Number of available cell lines	Number of available features	Number of features after autoencoder	Number of features after PCA	Explained variance (PCA)
Copy Number Variation	1043	23316	466		0.91
Methylation	843	56146	1122		0.92
Global Chromatin Profiling	897	42	21		0.99
Metabolomics	928	225	112		0.99
microRNA	954	734	73		0.95
Proteomics	899	214	107		0.93

Model evaluation and performance metrics

After data acquisition and pre-processing, we gather all datasets and to evaluate the results in the most unbiased manner possible, we randomly isolated three datasets considering different scenarios:

i) leave cell out dataset - 3 randomly chosen cell lines belonging to different tissue types (yielding 5500 synergistic and 4945 non-synergistic samples) (for the tissue type classification see **Figure W1** of the SYNPRED webserver);

ii) leave drugs out dataset - 5 drugs with the majority belonging to different hierarchical clusters (yielding 9469 synergistic and 8921 non-synergistic samples) (for drugs hierarchical clustering see **Figure W7** of the SYNPRED webserver);

iii) leave drug combinations out dataset - 5 drug combinations (yielding 125 synergistic and 85 non-synergistic samples).

Finally, we split the remaining data into training (76.328 synergistic and 67.117 non-synergistic samples) and test sets (18.875 synergistic and 16.719 non-synergistic samples) on an 80/20 ratio (**Table 3_Supplementary Material**). The binary classification models were evaluated through accuracy (acc), precision (prec), recall (rec), Area Under the Receiver Operating Characteristic (AUROC) as well as F1-score as previously described [49]. The regression models were evaluated through Root-Mean-

Square Deviation (RMSE), Mean Squared Error (MSE), coefficient of determination (R^2), Mean Absolute Error (MAE) [50], Pearson and Spearman correlation coefficients [51].

Development of Machine Learning models

Neural Networks with Keras. The classification and regression neural networks were fully developed using Keras with a TensorFlow (Version 2.3.1) backend [46]. Weights were updated using Adam optimizer [47] and a learning rate of 0.0001 along 125 epochs with binary cross-entropy (classification) and MSE (regression) as the loss functions. All the HL were connected through ReLU activation, while the output layer was subject to sigmoid (classification) or linear activation (regression). As an initial approach, we performed a gridsearch for parameter optimization using 10% of the training set, fully detailed in the "Parameter optimization" section. The best performing parameters were further selected and used to train the models with the complete train dataset.

Machine Learning algorithms with scikit-learn. The datasets presented in this work were also trained with the most commonly used algorithms for synergy prediction tasks, namely with Random Forest (RF) [52], Extreme Randomized Trees (ETC) [49,53], Support Vector Machines (SVM) [54], Stochastic Gradient Descent (SGD) [55], k-Nearest Neighbours (kNN) [56], and Extreme Gradient Boosting (XGBoost) [57]. The RF, ETC, SVM, SGD, and kNN models were built using the Python package "SciKit Learn" (Version 0.22.1) [35]. The XGBoost model was built using its dedicated package for Python (available at the Python Package Index as "xgboost") [57]. These six algorithms were also subject to gridsearch for parameter's optimization using 10% of the training set as described in the "Parameter optimization" section, with the best ones used to train the models with the full dataset.

Parameter optimization. To properly perform parameters' optimization in all the algorithms described, a grid search was performed using in-house scripts for Keras DL models and scikit-learn's GridSearchCV with 3-fold cross-validation (for ML algorithms with scikit-learn). We used 10% of the training set [58], a value in agreement with subset usage for parameter optimization [59], since using the full training dataset would exponentially increase an already long task. For each of the Keras classification and regression DL models, we performed gridsearch with 288 runs with parameters covering the four available dimensionality reduction datasets (PCA, PCA_drop, autoencoder, autoencoder_drop), 18 different network architectures, and four different dropout rates (0.00, 0.25, 0.50, 0.75) (**Table 4_Supplementary Material**). In the case of each of the six classification and regression

ML models trained with scikit-learn, we used a total of 820 runs, including different parameters and dataset combinations (**Table 5_Supplementary Material**). Finally, for the five possible targets (full agreement, Bliss, HSA, Loewe, and ZIP), we trained each of the six ML models with the best corresponding performing parameters. We then assessed the best performing architectures and dropout rates for the DL-based models. For each of the possible evaluation metrics we then trained the best performing parameters which can lead to different number of DL-based models depending on the synergy reference model used due to parameters overlap.

Ensemble algorithms. After selecting the previous best-performing models we replaced the outliers by the average of the remaining prediction values. We considered outliers, the synergy prediction values above or below 10 times the average of the remaining prediction values. These were used to constitute a new feature representation of the samples that could undergo ensemble model training. The ensemble models were first subjected to a new gridsearch for parameter optimization (**Table 6_Supplementary Material**), taking the target probability of the selected algorithms as features, ultimately developing a neural network that worked as an ensemble method. This neural network had a learning rate of 0.0001, trained for three epochs, used the Adam optimizer [47] and binary cross-entropy and MSE for classification and regression, respectively, as the loss functions. All the HL were connected through ReLU activation, while the output layer was subject to sigmoid or linear activation for classification and regression, respectively. The best-performing ensemble models were trained with the prediction-based feature space.

Feature Contribution

To understand what the top contributors were to the most for accurate predictions, we assessed their predictive power. For that, we needed first to break down the process of assessing feature contribution into two stages due to the dimensionality reduction of cell lines. First, since the best performing dimensionality reduction approach was the PCA, we considered the explained variance by each of the features concerning the respective Principal Component (PC). This information was then extracted as an attribute from the PCA object using scikit-learn [35]. Secondly, we used the eli5 package [60], with Python deployment, to assess final feature weight by deploying Permutation Importance [52], a method that allows iterative exclusion of each of the features, to assess its contribution to the predictive model. The Permutation Importance was deployed on the test set because it would not be possible to assess

the feature contribution under unbiased conditions if the training set had been used. However, it is worth noting that this evaluation occurs after all model training; hence, it does not influence the test results.

Benchmark

To ensure the final model's generalizability, we performed a literature review to search for databases that could be used to perform the benchmark and for which we could calculate/retrieve the necessary features. The O'Neil benchmark dataset could not be used as, after processing the dataset, there were not enough single dose responses associated with each concentration in the combination datasets [61]. Regarding the Forcina [62] and Mathews [63] datasets, the cell lines used were not available at CCLE, which renders cell line-associated feature extraction impossible. The chosen dataset that matched all the benchmark requirements was the DECREASE [27], comprising 210 unique drug-drug-cell line combinations corresponding to 34 drugs and 13 cell lines. All the developed methods were then tested against the DECREASE database, and the performance was assessed using classification or regression evaluation metrics, as described above.

Web-based application interface implementation

The SYNPRED prediction models were implemented in a web-based application at http://www.moreiralab.com/resources/synpred/. The website's plots and front-end were constructed with plotly [64] and Flask [65], both freely available Python packages, on a framework that uses an in-house adaptation of Javascript, CSS, and HTML scripts. All the back-end hosting was mediated with Flask [65].

RESULTS AND DISCUSSION

Measuring feature importance for model development

To understand the importance of each group of included features for the final model performance and to attain a more interpretable model, we analysed each of the individual models with Permutation Importance. We perceived that more complex models, particularly DL-based models with different architectures, tend to make more extensive use of the omics-based features to over 70% of the total feature contribution (**Figures W8-W12** of the SYNPRED webserver). Contrarily, simpler models, such as kNN and SGD, made almost exclusive use of the drug features (above 90%) (**Figures W16** and

W18 of the SYNPRED webserver). Other non-DL based models made variable (between 20%-80%) usage of the omics features (**Figures W13-W15** and **W17** of the SYNPRED webserver). This observation highlights the importance of DL models to take full advantage of omics data by capturing the complexity of each cancer profile, and thus improving drug pair-cell line combinations predictions. The advantages of using these algorithms when dealing with multi-dimensional omics data, particularly the great flexibility of DL architectures, was also previously emphasised [66].

We then looked for a possible biological relevance of the Top 5 genes in each group of the most critical multiomics features to understand if genes contributing more for the prediction models were also implicated in tumorigenesis. Of the 15 ranked genes from expression, methylation and CNV variations, all of them, are used as prognostic cancer markers, or have a role in tumour progression and treatment (**Table 3**). These data suggest that our models, especially DNNs, are likely to capture the most relevant information for each group of multiomics features for synergistic drug combinations. The remaining ranked genes organised by each ML model's best-contributing features are presented in interactive Sankey diagrams on the website landing page (**Figures W8-W18**).

Table 3. Permutation importance of the Top 5 proteins associated with expression, methylation and CNV features as well as their associated biological relevance.

Type of Feature	Gene Name	Protein Description	Biological relevance [a]
**Expression**	LINC01993	Long Intergenic Non-Protein Coding RNA 1993	IncRNA significantly upregulated in metastatic tumour samples [67]
	IFI16	Interferon gamma inducible protein 16	Prognostic marker in renal cancer (unfavourable)
	CPEB1	Cytoplasmic polyadenylation element binding protein 1	Lack of this gene is associated with tumour growth [68]
	LINC01771	Long Intergenic Non-Protein Coding RNA 1771	Differentially expressed genes in breast cancer [69]

Type of Feature	Gene Name	Protein Description	Biological relevance [a]
	HNRNPCL1	Heterogeneous nuclear ribonucleoprotein C-like 1	Potential biomarker of Lynch Syndrome Endometrial Cancer [70]
**Methylation**	C8orf44[1]-SGK3[2]	Chromosome 8 open reading frame 44-Serum/glucocorticoid regulated kinase family member 3	[1] Prognostic marker in renal cancer (unfavourable), urothelial cancer (favourable) and pancreatic cancer (favourable)<br><br>[2] Prognostic marker in breast cancer (favourable), cervical cancer (favourable) and pancreatic cancer (favourable)
	PERM1	PPARGC1 and ESRR induced regulator, muscle 1	Prognostic marker in renal cancer (unfavourable)
	PRKRA-AS1	Cholesterol Induced Regulator Of Metabolism RNA (CHROMR)	Role in cholesterol metabolism (reported to drive tumour growth and invasion). This gene is 3.4-fold up-regulated in rituximab-resistant diffuse large B-cell lymphoma cell lines [71]
	LIN54	Lin-54 DREAM MuvB core complex component	Prognostic marker in renal cancer (favourable)
	ZYG11A	Zyg-11 family member A, cell cycle regulator	Potential oncogene in non-small cell lung cancer [72]
**CNV**	FAM19A2	Family with sequence similarity 19 member A2, C-C motif chemokine like	Potential role on glioma prognosis [73]

Type of Feature	Gene Name	Protein Description	Biological relevance [a]
	MIR548AD	MicroRNA 548ad	Fusion gene (MIR548AD-ALK) with a possible role in the clinical outcomes of lung cancer [74]
	ROCK1P1	Rho Associated Coiled-Coil Containing Protein Kinase 1 Pseudogene 1	Common mutated pseudogene in osteosarcoma [75]
	PHACTR3	Phosphatase and actin regulator 3	Potential biomarker for early detection of colorectal cancer [76], and diagnosis of lung cancer [77]. Role in non-small-cell lung cancer progression [78]
	PPP1R17	Protein phosphatase 1 regulatory subunit 17	Differentially expressed genes in corticotrophinomas [79]

[a] The protein description and biological importance were retrieved from The Human Proteins Atlas (https://www.proteinatlas.org/) and The Human Gene Database (https://www.genecards.org/). When this information was not listed in these databases, we presented the study that supports the biological relevance. Favourable and unfavourable is related to gene contribution for cancer progression.

Tuning and choosing the best Machine Learning parameters

ML performance and training time are deeply affected by specific model parameters, so an appropriate choice of the best ones should always be performed. With that in mind, we used a gridsearch approach to test a comprehensive array of parameters and dataset combinations, including parameters for several ML methods, a comprehensive set of DL configurations and pre-processing setups, as described above. Regarding the pre-processing datasets, autoencoder datasets performed worse in the training sets and slightly worse for the test set, which led us to discard it as there was no benefit to the increased training time caused by the significantly higher dimensionality. We proceed with the dataset in which PCA was used for dimensionality reduction and replacing the missing values with 0, as these approaches performed better for most gridsearch runs [80,81].

SYNPRED models for drug combination prediction

After selecting the best parameters for both DL with Keras and ML with scikit-learn, we trained models with the full training set according to the parameters in the best gridsearch performing metrics. The best individual models were used to attain each sample prediction to make the final ensemble for the four-synergy reference model plus the full-agreement. The final models were then evaluated in the test set and three different scenarios: leave cell out, leave drugs out and leave drug combinations out**,** by attaining different classification (**Table 7_Supplementary Material**) or regression (**Table 8-11_Supplementary Material**) evaluation metrics.

Classification model performance. Prior to ensemble development, the best independent performing model was XGBoost with the following parameters: alpha = 0, max_depth = 6, n_estimators = 1000. After ensemble, our final full-agreement SYNPRED comprised five DL-based and six ML-based models, attained with a DL architecture with three hidden layers of size ten and a dropout rate of 0.40. When applied in an independent test set, our ensemble model displayed better performance (accuracy=0.80, precision=0.81, recall=0.81, AUROC=0.80, and F1-score=0.81) than any other classic ML or DL models, including reference ones such as SVM, RF or XGBoost frequently used for synergy prediction classification tasks (**Table 4, Table 7_Supplementary Material**) [12,13,82]. In the three independent scenarios, the full-agreement SYNPRED achieved higher precision values by returning the most relevant results than any other of the individual models, however with a lower performance in terms of the metrics that include the number of false negatives such as recall, AUC and F1-score.

Table 4. Best results obtained for the classification ensemble model.

**Subset used for evaluation [a]**	**Accuracy**	**Precision**	**Recall**	**AUROC**	**F1-Score**
**Test**	0.80	0.81	0.81	0.80	0.81
**Leave cells out**	0.67	0.73	0.52	0.67	0.61
**Leave drugs out**	0.60	0.78	0.45	0.63	0.57
**Leave drug combinations out**	0.68	0.72	0.62	0.68	0.67

[a] The final model had a dropout rate of [0.4] and an architecture of [10, 10, 10].

Regression model performance. Concerning the four regression tasks, overall, ZIP (**Table 11_Supplementary Material**) and Bliss (**Table 8_Supplementary Material**) stand out - in either the

metrics or the datasets considered - while HSA (**Table 9_Supplementary Material**) followed closely behind. On the other hand, Loewe (**Table 10_Supplementary Material**) showed a significantly lower performance, foreseeable by the distribution values of this synergy reference model. Compared to the other models, Loewe showed much steeper outliers, regardless of the cell line or tissue in study (**Figure 2, Figure W1-W5 on the SYNPRED Webserver**). Loewe's interval of possible values surveyed was 5287.15, significantly higher than the remaining ranges (Bliss: 301.12; HSA: 317.68; ZIP: 297.42). This discrepancy correlates with the poorer performance of its dedicated predictor and hints at a problem with the synergy reference model itself. Although in agreement with the presented data, this is unexpected considering the literature on the subject, which systematically uses it as the target regression reference model [13,15–17,19,20]. For most cases in which this happens, there is no comparison with the remaining reference models, which could benefit the attained models. The few available comparative studies are mainly done outside of the spectrum of synergy prediction and somewhat under the scope of analysing provided drug combination dose-response matrix data [32,83]. By deploying unbiased data-driven selection of the model, SYNPRED empirically assesses how realistically viable is the representation of four of the most common synergy reference models against a real biological dataset.

The results of our best final ensemble regression models (ZIP and Bliss) outperformed all the individual predictors when evaluated in the test dataset and leave drug combinations out scenario, one of the most challenging ones. Regarding correlation metrics, ZIP achieves strong Pearson values (0.80 on test and 0.77 on the leave drug combinations out dataset) from the literature standards [84]. The ZIP had 7.10 and 7.93 RMSE on the test and combination datasets, respectively, regarding scale-depending performance metrics. Considering that ZIP values range within [-129.43, 167.99], our predictor was able to determine ZIP synergy values with a low error (**Figure 3**). A very similar pattern was exhibited by the Bliss ensemble predictor (**Figure 4**).

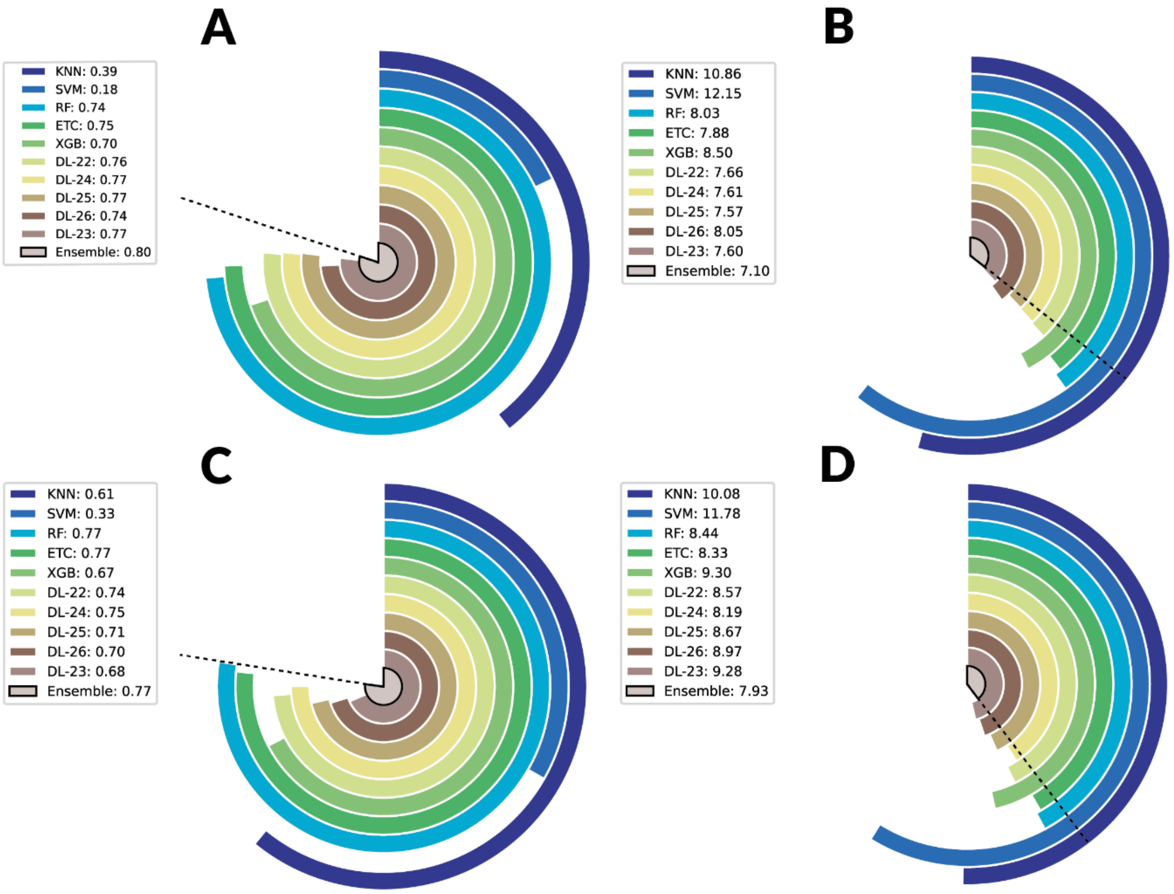

Figure 3. Circular bar plot representing the model's evaluation metrics for the ZIP synergy reference model. In each plot, the dashed line marks the higher value for Pearson and the lower value for RMSE. (A) Models performance Pearson values evaluated in the test dataset, (B) Models performance RMSE values evaluated in the test dataset, (C) Models performance Pearson values evaluated in the leave drug combinations out dataset, (D) Models performance RMSE values evaluated in the leave drug combinations out dataset.

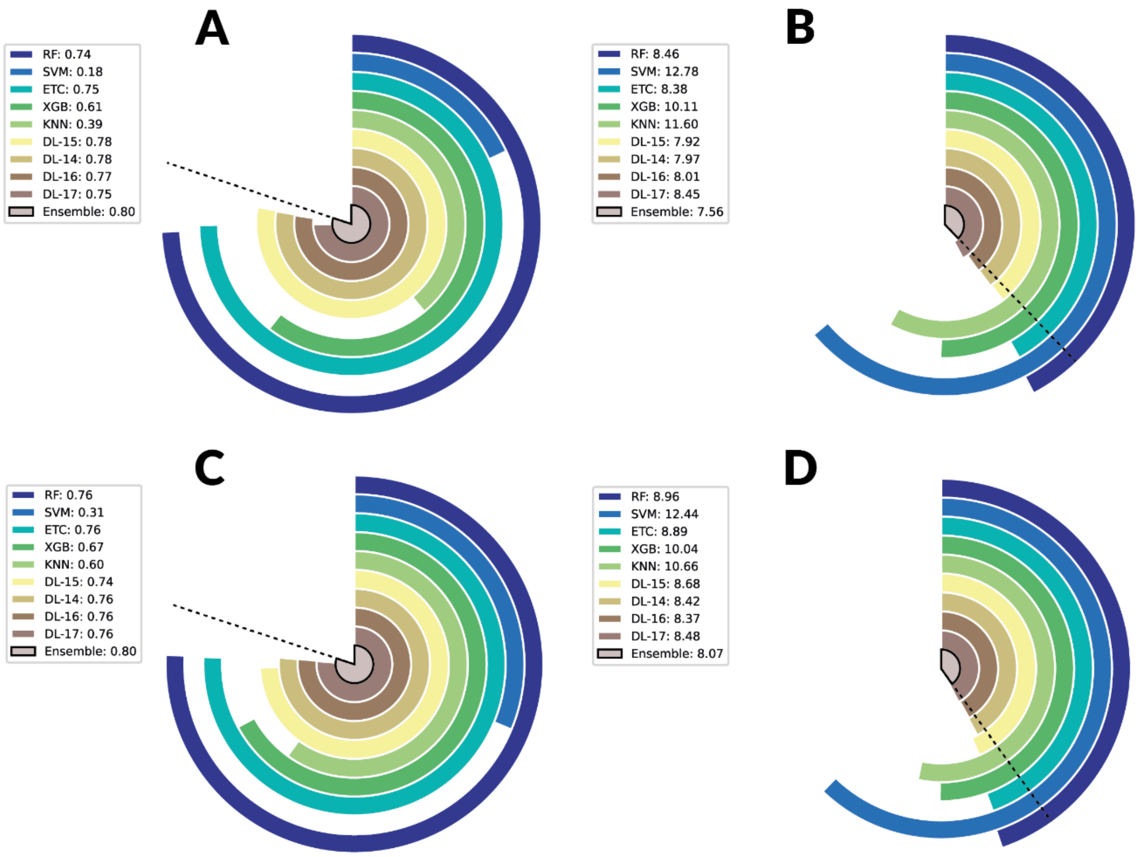

Figure 4. Circular bar plot representing the model's evaluation metrics for the Bliss synergy reference model. In each plot, the dashed line marks the higher value for Pearson and the lower value for RMSE. (A) Models performance Pearson values evaluated in the test dataset, (B) Models performance RMSE values evaluated in the test dataset, (C) Models performance Pearson values evaluated in the leave drug combinations out dataset, (D) Models performance RMSE values evaluated in the leave drug combinations out dataset.

Benchmarking with an independent dataset

The DECREASE database [27] containing 34 drugs, 13 cell lines, and 210 drug-drug-cell lines combinations was processed to attain the features associated with its data using the same pipeline applied to SYNPRED. We disregarded all samples that were not present in CCLE, since it will make the acquisition of multiomics features impossible. When calculating the class with our strict full-agreement requirements, all the possible samples turned out to be negative, which indicates that the dataset, although appropriate, seems to be limited in the range of synergy values for both classification and regression tasks. When predicting the class, our SYNPRED ensemble achieved 0.91 of accuracy

using the classification model and a Pearson of 0.61 when using the regression model with the ZIP synergy reference model.

Web-based application description

The classification and regression models for predicting the type of combinatory effect in dug pair-cell line samples is available as a web-based application at http://www.moreiralab.com/resources/synpred/. All the 11 described single models are deployed on user submission, as well as the ensemble approach. The user needs to submit two drugs as input in the *.smile format and selects from a dropdown menu, the primary body site corresponding to the tested cancer cell lines. The drugs are then subject to feature extraction by Mordred and a standard pre-processing (feature elimination and normalization) as thoroughly described in the methods section. The output, displayed in a downloadable heatmap, is the drug combination prediction effect for each of the individual cell lines calculated with the ensemble classification and regression models and using four synergy reference models (ZIP, HSA, Bliss, Loewe) plus the full-agreement metric. Furthermore, it is also displayed in the last column (“Synergy Votes”) the final tally of synergistic queries predicted by all models based on the prediction values. This facilitates the visualization of the type of combinatory effect between the two drugs and aims at strengthening the value of the prediction due to the lack of consensus between the difference synergy reference models. The results are returned to the provided email and displayed on the submission web page. Additionally, users can assess, explore, and visualize through different plots as well as export a summary of the synergy scores (calculated using ZIP, Bliss, HSA and Loewe synergy reference models) by cell line used to develop the original dataset of SYNPRED. To our knowledge, this is the first webserver that can predict new drug synergy combinations without the need of uploading a partial or full dose-response matrix. This feature is an advantage compared with other models implemented in webservers that need these types of data for drug combination response prediction [27,28].

CONCLUSIONS

Synergistic anticancer drug combinations are a powerful tool to help tackle cancer drug resistance since they can simultaneously target multiple key molecules or pathways. The rational design of combination therapies is warranted to improve the efficacy, although this is a well-known time-consuming and expensive task. In recent years, ML algorithms' applicability for drug-repurposing or novel drug design has been essential to demonstrate the importance of *in silico* methodologies to help overcome this

problem. Some classification [12,13,16] and regression [15,17–21] models using ML and omics data for predicting drug synergy combinations were already developed. However, the fittingness of the previously developed algorithms is sometimes hindered by using a single reference model (e.g., Bliss, Loewe, HSA, or ZIP) or by the difficulty in applying these models on new unseen data, since these are not straightforward to implement and require advanced bioinformatics skills. Additionally, our study leads to an innovative approach by highlighting the importance of choosing an appropriated synergy reference model and explores this choice influence in the final predictor performance. Given the different sensitivity observed between these reference models in evaluating the degree of combination, a more comprehensive and rigorous approach that leverages all metrics to predict drug synergy is an asset.

This study introduced a new synergy prediction model, SYNPRED, that combines comprehensive multiomics data of cancer cell lines with physicochemical and structural features of drugs. This work is one of the first that takes four different synergy reference models (Bliss, HSA, Loewe, and ZIP) and uses one of the most comprehensive and balanced databases regarding the synergistic-non synergistic distribution, the NCI-ALMANAC. Our top-ranked classification and regression models, an ensemble developed with the best machine learning models, achieved state-of-the-art performance to predict synergistic drug combinations in an independent dataset. Besides, we provide the complete workflow for a standalone deployment in our GitHub coupled with a freely available and easy-to-use webserver (http://www.moreiralab.com/resources/synpred/) that only requires two drugs' SMILES as inputs, thus alleviating the need of uploading a conventional and laborious dose-response matrix. SYNPRED can be a valuable tool to the scientific and medical community for drug repurposing or *in-silico* discovery of new anticancer drug combinations.

Additionally, given the importance of multiomics data in cell line classification and therapy response, we combined all the available multiomics features in the CCLE database to explore their individual contribution to model development. The knowledge mined from this analysis demonstrates the capacity of different ML models to deal with multiomics data, with DL algorithms being much more able to learn and leverage this complex type of features. We found that the most ranked proteins in each of the most contributing multiomics features are important cancer biomarkers or have a role in tumorigenesis, demonstrating DNN models' capacity to capture their significance and use this information for the final

model development. In the future, we expect to include protein-protein interactions data and network analysis to improve the model performance, aiming to identify drug combinations with potential new targets across different cell lines.

AVAILABILITY OF SOURCE CODE AND REQUIREMENTS

Project name: SYNPRED

Project home page: https://github.com/MoreiraLAB/synpred

Operating system(s): Linux, Mac OS X, Windows

Programming language: Python and R

Other requirements: Python 3.8.2 or higher, R 3.6.3 or higher

License: GPL-3.0

DATA AVAILABILITY

SYNPRED is a free, open-source web-based application available for non-commercial use at http://www.moreiralab.com/resources/synpred/ without any login or registration requirements. The source code of the web-based application implementation is deposited in the GitHub repository (https://github.com/MoreiraLAB/synpred) to allow the stand-alone use of the application and further integration and comparison with other models. The code is fully developed in Python and R languages; hence, it can be deployed fully without charge. The multiomics data included in this study are available at the corresponding references mentioned in the main text.

ADDITIONAL FILES

Supplementary Table 1. Conditions for dimensionality reduction with autoencoders. Hidden and bottleneck layers definition according to the Number of Features.

Supplementary Table 2. Conditions for dimensionality reduction with autoencoders. Number of epochs of the autoencoder training according to either the Number of Samples or Number of Features.

Supplementary Table 3. Final datasets to be subjected to training.

Supplementary Table 4. Gridsearch combination parameters using 10% on the training set with Deep Learning algorithms.

Supplementary Table 5. Gridsearch combination parameters using 10% on the training set with non-Deep Learning algorithms.

Supplementary Table 6. Gridsearch combination parameters of the ensemble neural network.

Table 7. Final metrics of the classification models evaluated in an independent test set and three different scenarios (leave cell out, leave drugs out and leave drug combinations out) using Full-Agreement synergy values.

Table 8. Final metrics of the regression models evaluated in an independent test set and three different scenarios (leave cell out, leave drugs out and leave drug combinations out) using Bliss synergy reference model.

Table 9. Final metrics of the regression models evaluated in an independent test set and three different scenarios (leave cell out, leave drugs out and leave drug combinations out) using HSA synergy reference model.

Table 10. Final metrics of the regression models evaluated in an independent test set and three different scenarios (leave cell out, leave drugs out and leave drug combinations out) using Loewe synergy reference model.

Table 11. Final metrics of the regression models evaluated in an independent test set and three different scenarios (leave cell out, leave drugs out and leave drug combinations out) using ZIP synergy reference model.

ABBREVIATIONS

AI: Artificial Intelligence; ACC: Accuracy; ANN: Artificial Neural Network; AUROC: Area Under the Receiver Operator Curve; CCLE: Cancer Cell Line Encyclopaedia; CNV: Copy Number Variation; DL: Deep Learning; DNN: Deep Neural Networks; ENS: Ensemble; ETC: Extreme Randomized Trees; F1: F1-score; GPU: Graphics Processing Unit; HL: Hidden Layers; HSA: Highest Single Agent; kNN: k-Nearest Neighbours; MAE: Mean Absolute Error; miRNA: micro-RNA; ML: Machine Learning; MLP: Multi-Layer Perceptron; MSE: Mean-Square Error; PC: Principal Component; PCA: Principal Component Analysis; PREC: Precision; R^2: Coefficient of Determination; REC: Recall; ReLU: Rectified Linear Unit; RF: Random Forest; RMSE: Root-Mean-Square Deviation; SGD: Stochastic Gradient Descent; SMILE: Simplified Molecular-Input Line-Entry System; SVM: Support Vector Machine; SYNPRED: SYNergy PREDiction; XGBoost: Extreme Gradient Boosting; ZIP: Zero Interaction Potency

COMPETING INTERESTS

The authors declare that they have no competing interests.

FUNDING

This work was supported by the European Regional Development Fund through the COMPETE 2020 - Operational Programme for Competitiveness and Internationalisation and Portuguese national funds via Fundação para a Ciência e a Tecnologia (FCT) [LA/P/0058/2020, UIDB/04539/2020, UIDP/04539/2020, POCI-01-0145-FEDER-031356, and DSAIPA/DS/0118/2020]. FCT also supported A.J.P. with a PhD scholarship [SFRH/BD/144966/2019].

AUTHOR CONTRIBUTIONS

António J. Preto, Methodology; Software; Validation; Formal analysis; Investigation; Resources; Writing – review & editing; Visualization. Pedro Matos-Filipe, Methodology; Software; Investigation; Resources; Data curation; Writing – original draft preparation. Joana Mourão, Conceptualization; Methodology; Formal analysis; Data curation; Writing – original draft preparation; Writing – review & editing; Supervision; Project administration. Irina S. Moreira, Conceptualization; Writing – review & editing; Visualization; Supervision; Project administration; Funding acquisition.

ACKNOWLEDGEMENTS

Authors would like to acknowledge STRATAGEM - New diagnostic and therapeutic tools against multidrug-resistant tumors, CA17104.

REFERENCES

1. IARC IA for R on C. GLOBOCAN - Cancer Tomorrow via Global Cancer Observatory. 2020.

2. Vasan N, Baselga J, Hyman DM. A view on drug resistance in cancer. *Nature*. 2019; doi: 10.1038/s41586-019-1730-1.

3. Chatterjee N, Bivona TG. Polytherapy and Targeted Cancer Drug Resistance. *Trends in Cancer*. 2019; doi: 10.1016/j.trecan.2019.02.003.

4. Roell KR, Reif DM, Motsinger-Reif AA. An Introduction to Terminology and Methodology of Chemical Synergy—Perspectives from Across Disciplines. *Front Pharmacol*. 2017; doi: 10.3389/fphar.2017.00158.

5. Brandão M, Pondé NF, Poggio F, Kotecki N, Salis M, Lambertini M, et al.. Combination therapies for the treatment of HER2-positive breast cancer: current and future prospects. *Expert Review of Anticancer Therapy*. 2018; doi: 10.1080/14737140.2018.1477596.

6. Westerweel PE, te Boekhorst PAW, Levin M-D, Cornelissen JJ. New Approaches and Treatment Combinations for the Management of Chronic Myeloid Leukemia. *Front Oncol*. 2019; doi: 10.3389/fonc.2019.00665.

7. Xu J, Qiu Y. Current opinion and mechanistic interpretation of combination therapy for castration-resistant prostate cancer. *Asian J Androl*. 2019; doi: 10.4103/aja.aja_10_19.

8. Ribas A, Lawrence D, Atkinson V, Agarwal S, Miller WH, Carlino MS, et al.. Combined BRAF and MEK inhibition with PD-1 blockade immunotherapy in BRAF-mutant melanoma. *Nat Med*. 2019; doi: 10.1038/s41591-019-0476-5.

9. Wang Z, Deisboeck TS. Dynamic Targeting in Cancer Treatment. *Front Physiol*. 2019; doi: 10.3389/fphys.2019.00096.

10. Wang Z, Li H, Guan Y. Machine Learning for Cancer Drug Combination. *Clin Pharmacol Ther*. 2020; doi: 10.1002/cpt.1773.

11. Janizek JD, Celik S, Lee S-I. Explainable machine learning prediction of synergistic drug combinations for precision cancer medicine. Cancer Biology; 2018 May.

12. Li H, Li T, Quang D, Guan Y. Network Propagation Predicts Drug Synergy in Cancers. *Cancer Res*. 2018; doi: 10.1158/0008-5472.CAN-18-0740.

13. Celebi R, Bear Don't Walk O, Movva R, Alpsoy S, Dumontier M. In-silico Prediction of Synergistic Anti-Cancer Drug Combinations Using Multi-omics Data. *Sci Rep*. 2019; doi: 10.1038/s41598-019-45236-6.

14. Malyutina A, Majumder MM, Wang W, Pessia A, Heckman CA, Tang J. Drug combination sensitivity scoring facilitates the discovery of synergistic and efficacious drug combinations in cancer. Gallo J, editor. *PLoS Comput Biol*. 2019; doi: 10.1371/journal.pcbi.1006752.

15. Zhang T, Zhang L, Payne PRO, Li F. Synergistic Drug Combination Prediction by Integrating Multiomics Data in Deep Learning Models. In: Markowitz J, editor. *Translational Bioinformatics for Therapeutic Development*. New York, NY: Springer US;

16. Wang J, Liu X, Shen S, Deng L, Liu H. DeepDDS: deep graph neural network with attention mechanism to predict synergistic drug combinations. *Briefings in Bioinformatics*. 2021; doi: 10.1093/bib/bbab390.

17. Preuer K, Lewis RPI, Hochreiter S, Bender A, Bulusu KC, Klambauer G. DeepSynergy: Predicting anti-cancer drug synergy with Deep Learning. *Bioinformatics*. 2018; doi: 10.1093/bioinformatics/btx806.

18. Zhang H, Feng J, Zeng A, Payne P, Li F. Predicting Tumor Cell Response to Synergistic Drug Combinations Using a Novel Simplified Deep Learning Model. Bioinformatics; 2020 Apr.

19. Kuru HI, Tastan O, Cicek AE. MatchMaker: A Deep Learning Framework for Drug Synergy Prediction. *IEEE/ACM Trans Comput Biol Bioinform*. 2021; doi: 10.1109/TCBB.2021.3086702.

20. Liu Q, Xie L. TranSynergy: Mechanism-driven interpretable deep neural network for the synergistic prediction and pathway deconvolution of drug combinations. Schlessinger A, editor. *PLoS Comput Biol*. 2021; doi: 10.1371/journal.pcbi.1008653.

21. Xia F, Shukla M, Brettin T, Garcia-Cardona C, Cohn J, Allen JE, et al.. Predicting tumor cell line response to drug pairs with deep learning. *BMC Bioinformatics*. 2018; doi: 10.1186/s12859-018-2509-3.

22. Bliss CI. The toxicity of poisons applied jointly. *Annals of Applied Biology*. 1939; doi: 10.1111/j.1744-7348.1939.tb06990.x.

23. Foucquier J, Guedj M. Analysis of drug combinations: current methodological landscape. *Pharmacology research & perspectives*. 2015; doi: 10.1002/prp2.149.

24. Loewe S, Muischnek H. Über Kombinationswirkungen. *Archiv für Experimentelle Pathologie und Pharmakologie*. 1926; doi: 10.1007/BF01952257.

25. Chou T-C. Drug Combination Studies and Their Synergy Quantification Using the Chou-Talalay Method. *Cancer Research*. 2010; doi: 10.1158/0008-5472.CAN-09-1947.

26. Yadav B, Wennerberg K, Aittokallio T, Tang J. Searching for Drug Synergy in Complex Dose-Response Landscapes Using an Interaction Potency Model. *Computational and structural biotechnology journal*. 2015; doi: 10.1016/j.csbj.2015.09.001.

27. Ianevski A, Giri AK, Gautam P, Kononov A, Potdar S, Saarela J, et al.. Prediction of drug combination effects with a minimal set of experiments. *Nat Mach Intell*. 2019; doi: 10.1038/s42256-019-0122-4.

28. Zagidullin B, Aldahdooh J, Zheng S, Wang W, Wang Y, Saad J, et al.. DrugComb: an integrative cancer drug combination data portal. *Nucleic Acids Research*. 2019; doi: 10.1093/nar/gkz337.

29. Holbeck SL, Camalier R, Crowell JA, Govindharajulu JP, Hollingshead M, Anderson LW, et al.. The National Cancer Institute ALMANAC: A Comprehensive Screening Resource for the Detection of Anticancer Drug Pairs with Enhanced Therapeutic Activity. *Cancer Research*. 2017; doi: 10.1158/0008-5472.CAN-17-0489.

30. Shoemaker RH. The NCI60 human tumour cell line anticancer drug screen. *Nature Reviews Cancer*. 2006; doi: 10.1038/nrc1951.

31. DCTD. DTP, DCTD Tumor Repository - A catalog of in vitro cell lines, transplantable animal and human tumors and yeast. National Cancer Institute at Frederick; 2020 Jul.

32. Zheng S, Wang W, Aldahdooh J, Malyutina A, Shadbahr T, Pessia A, et al.. SynergyFinder Plus: towards a better interpretation and annotation of drug combination screening datasets. Bioinformatics; 2021 Jun.

33. Kim S, Chen J, Cheng T, Gindulyte A, He J, He S, et al.. PubChem 2019 update: improved access to chemical data. *Nucleic Acids Research*. 2018; doi: 10.1093/nar/gky1033.

34. Moriwaki H, Tian Y-S, Kawashita N, Takagi T. Mordred: a molecular descriptor calculator. *Journal of Cheminformatics*. 2018; doi: 10.1186/s13321-018-0258-y.

35. Pedregosa F, Varoquaux G, Gramfort A, Michel V, Thirion B, Grisel O, et al.. Scikit-learn: Machine Learning in Python. *Journal of Machine Learning Research*. 12:2825–302011;

36. Barretina J, Caponigro G, Stransky N, Venkatesan K, Margolin AA, Kim S, et al.. The Cancer Cell Line Encyclopedia enables predictive modelling of anticancer drug sensitivity. *Nature*. 2012; doi: 10.1038/nature11003.

37. Bairoch A. The Cellosaurus, a Cell-Line Knowledge Resource. *J Biomol Tech*. 2018; doi: 10.7171/jbt.18-2902-002.

38. Ghandi M, Huang FW, Jané-Valbuena J, Kryukov GV, Lo CC, McDonald ER, et al.. Next-generation characterization of the Cancer Cell Line Encyclopedia. *Nature*. 2019; doi: 10.1038/s41586-019-1186-3.

39. The International HapMap Consortium. The International HapMap Project. *Nature*. 2003; doi: 10.1038/nature02168.

40. Venkatraman ES, Olshen AB. A faster circular binary segmentation algorithm for the analysis of array CGH data. *Bioinformatics*. 2007; doi: 10.1093/bioinformatics/btl646.

41. Chaudhary K, Poirion OB, Lu L, Garmire LX. Deep Learning–Based Multi-Omics Integration Robustly Predicts Survival in Liver Cancer. *Clin Cancer Res*. American Association for Cancer Research; 2018; doi: 10.1158/1078-0432.CCR-17-0853.

42. Zhang L, Lv C, Jin Y, Cheng G, Fu Y, Yuan D, et al.. Deep Learning-Based Multi-Omics Data Integration Reveals Two Prognostic Subtypes in High-Risk Neuroblastoma. *Front Genet*. Frontiers; 2018; doi: 10.3389/fgene.2018.00477.

43. Simidjievski N, Bodnar C, Tariq I, Scherer P, Andres Terre H, Shams Z, et al.. Variational Autoencoders for Cancer Data Integration: Design Principles and Computational Practice. *Front Genet*. Frontiers; 2019; doi: 10.3389/fgene.2019.01205.

44. Hinton GE, Salakhutdinov RR. Reducing the Dimensionality of Data with Neural Networks. *Science*. American Association for the Advancement of Science; 2006; doi: 10.1126/science.1127647.

45. Wang Y, Yao H, Zhao S. Auto-encoder based dimensionality reduction. *Neurocomputing*. 2016; doi: 10.1016/j.neucom.2015.08.104.

46. Abadi M, Agarwal A, Barham P, Brevdo E, Chen Z, Citro C, et al.. TensorFlow: Large-Scale Machine Learning on Heterogeneous Distributed Systems. 2015;

47. Kingma DP, Ba J. Adam: A Method for Stochastic Optimization. *arXiv:14126980 [cs]*. 2017;

48. Meng C, Oana A. Zeleznik, Gerhard G. Thallinger, Bernhard Kuster, Amin M. Gholami, Aedín C. Culhane. Dimension reduction techniques for the integrative analysis of multi-omics data. *Briefings in Bioinformatics*. 2016; doi: 10.1093/bib/bbv108.

49. Preto AJ, Moreira IS. SPOTONE: Hot Spots on Protein Complexes with Extremely Randomized Trees via Sequence-Only Features. *IJMS*. 2020; doi: 10.3390/ijms21197281.

50. Botchkarev A. A New Typology Design of Performance Metrics to Measure Errors in Machine Learning Regression Algorithms. *IJIKM*. 2019; doi: 10.28945/4184.

51. de Winter JCF, Gosling SD, Potter J. Comparing the Pearson and Spearman correlation coefficients across distributions and sample sizes: A tutorial using simulations and empirical data. *Psychological Methods*. 2016; doi: 10.1037/met0000079.

52. Breiman L. Random Forests. *Machine Learning*. 45:5–322001;

53. Geurts P, Ernst D, Wehenkel L. Extremely randomized trees. *Machine Learning*. 63:3–422006;

54. Fan RE, Chang KW, Hsieh CJ, Wang XR, Lin CJ. LIBLINEAR: A library for large linear classification. *Journal of Machine Learning Research*. 9:1871–42008;

55. Zadrozny B, Elkan C. Transforming classifier scores into accurate multiclass probability estimates. *KDD ’02: Proceedings of the eighth ACM SIGKDD international conference on Knowledge discovery and data mining*. 2002; doi: 10.1145/775047.775151.

56. Altman NS. An introduction to kernel and nearest-neighbor nonparametric regression. *The American Statistician*. 46:175–851992;

57. Chen T, Guestrin C. XGBoost: A Scalable Tree Boosting System. *Proceedings of the 22nd ACM SIGKDD International Conference on Knowledge Discovery and Data Mining*. 2016; doi: 10.1145/2939672.2939785.

58. Noemí DeCastro-García, Ángel Luis Muñoz Castañeda, David Escudero García, Miguel V. Carriegos. Effect of the Sampling of a Dataset in the Hyperparameter Optimization Phase over the Efficiency of a Machine Learning Algorithm. *Advances in Complex Systems and Their Applications to Cybersecurity*. 2019; doi: 10.1155/2019/6278908.

59. Swersky K, Snoek J, Adams RP. Multi-Task Bayesian Optimization. *NIPS'13: Proceedings of the 26th International Conference on Neural Information Processing Systems*. 2:2004–122013;

60. Mikhail Korobov, Konstantin Lopuhin. ELI5.

61. O'Neil J, Benita Y, Feldman I, Chenard M, Roberts B, Liu Y, et al.. An Unbiased Oncology Compound Screen to Identify Novel Combination Strategies. *Mol Cancer Ther*. 2016; doi: 10.1158/1535-7163.MCT-15-0843.

62. Forcina GC, Conlon M, Wells A, Cao JY, Dixon SJ. Systematic Quantification of Population Cell Death Kinetics in Mammalian Cells. *Cell Systems*. 2017; doi: 10.1016/j.cels.2017.05.002.

63. Mathews Griner LA, Guha R, Shinn P, Young RM, Keller JM, Liu D, et al.. High-throughput combinatorial screening identifies drugs that cooperate with ibrutinib to kill activated B-cell–like diffuse large B-cell lymphoma cells. *Proc Natl Acad Sci USA*. 2014; doi: 10.1073/pnas.1311846111.

64. Plotly Technologies Inc. Collaborative data science. Plotly. Montréal, QC: Plotly Technologies Inc. Collaborative data science;

65. Grinberg M. Flask web development: developing web applications with python. O'Reilly Media, Inc.;

66. Grapov D, Fahrmann J, Wanichthanarak K, Khoomrung S. Rise of Deep Learning for Genomic, Proteomic, and Metabolomic Data Integration in Precision Medicine. *OMICS: A Journal of Integrative Biology*. 2018; doi: 10.1089/omi.2018.0097.

67. Wang J, Xiao K, Hou F, Tang L, Luo D, Liu G, et al.. POU2F1 Promotes Cell Viability and Tumor Growth in Gastric Cancer through Transcriptional Activation of lncRNA TTC3-AS1. Palmirotta R, editor. *Journal of Oncology*. 2021; doi: 10.1155/2021/5570088.

68. Nagaoka K, Fujii K, Zhang H, Usuda K, Watanabe G, Ivshina M, et al.. CPEB1 mediates epithelial-to-mesenchyme transition and breast cancer metastasis. *Oncogene*. 2016; doi: 10.1038/onc.2015.350.

69. Zhao S, Geng Y, Cao L, Yang Q, Pan T, Zhou D, et al.. Deciphering the performance of polo-like kinase 1 in triple-negative breast cancer progression according to the centromere protein U-phosphorylation pathway. *Am J Cancer Res*. 11:2142–582021;

70. Gao Y, Zhang X, Wang T, Zhang Y, Wang Q, Hu Y. HNRNPCL1, PRAMEF1, CFAP74, and DFFB: Common Potential Biomarkers for Sporadic and Suspected Lynch Syndrome Endometrial Cancer. *CMAR*. 2020; doi: 10.2147/CMAR.S262421.

71. Karstensen KT, Schein A, Petri A, Bøgsted M, Dybkær K, Uchida S, et al.. Long Non-Coding RNAs in Diffuse Large B-Cell Lymphoma. *ncRNA*. 2020; doi: 10.3390/ncrna7010001.

72. Wang X, Sun Q, Chen C, Yin R, Huang X, Wang X, et al.. *ZYG11A* serves as an oncogene in non-small cell lung cancer and influences *CCNE1* expression. *Oncotarget*. 2016; doi: 10.18632/oncotarget.6904.

73. Zhang D, Zhao J, Han C, Liu X, Liu J, Yang H. Identification of hub genes related to prognosis in glioma. *Bioscience Reports*. 2020; doi: 10.1042/BSR20193377.

74. Dou Y, Duan Q, Qi C, Hou L, Wang H. An intergenic region ALK fusion identified by DNA sequencing and validated by IHC in an early-stage lung adenocarcinoma. *J Cancer Res Clin Oncol*. 2021; doi: 10.1007/s00432-021-03526-5.

75. Hou J, Liu G, Zhang P, Wang B, Yan Q, Wu P, et al.. Experimental Study of Somatic Variants of Osteosarcoma by Whole-Exome Sequencing. *Med Sci Monit*. 2020; doi: 10.12659/MSM.920826.

76. Bosch LJW, Oort FA, Neerincx M, Khalid-de Bakker CAJ, sive Droste JST, Melotte V, et al.. DNA Methylation of Phosphatase and Actin Regulator 3 Detects Colorectal Cancer in Stool and Complements FIT. *Cancer Prev Res*. 2012; doi: 10.1158/1940-6207.CAPR-11-0315.

77. Hubers AJ, Heideman DAM, Burgers SA, Herder GJM, Sterk PJ, Rhodius RJ, et al.. DNA hypermethylation analysis in sputum for the diagnosis of lung cancer: training validation set approach. *Br J Cancer*. 2015; doi: 10.1038/bjc.2014.636.

78. Bankovic J, Stojsic J, Jovanovic D, Andjelkovic T, Milinkovic V, Ruzdijic S, et al.. Identification of genes associated with non-small-cell lung cancer promotion and progression. *Lung Cancer*. 2010; doi: 10.1016/j.lungcan.2009.04.010.

79. de Araújo LJT, Lerario AM, de Castro M, Martins CS, Bronstein MD, Machado MC, et al.. Transcriptome Analysis Showed a Differential Signature between Invasive and Non-invasive Corticotrophinomas. *Front Endocrinol*. 2017; doi: 10.3389/fendo.2017.00055.

80. Pedersen A, Mikkelsen E, Cronin-Fenton D, Kristensen N, Pham TM, Pedersen L, et al.. Missing data and multiple imputation in clinical epidemiological research. *CLEP*. 2017; doi: 10.2147/CLEP.S129785.

81. Zhang Z. Missing data imputation: focusing on single imputation. *Ann Transl Med*. 2016; doi: 10.3978/j.issn.2305-5839.2015.12.38.

82. Gilvary C, Dry JR, Elemento O. Multi-task learning predicts drug combination synergy in cells and in the clinic. Cancer Biology; 2019 Mar.

83. Di Veroli GY, Fornari C, Wang D, Mollard S, Bramhall JL, Richards FM, et al.. Combenefit: an interactive platform for the analysis and visualization of drug combinations. *Bioinformatics*. 2016; doi: 10.1093/bioinformatics/btw230.

84. Akoglu H. User's guide to correlation coefficients. *Turkish Journal of Emergency Medicine*. 2018; doi: 10.1016/j.tjem.2018.08.001.

Click here to access/download;Figure;Graphical_Abstract.png

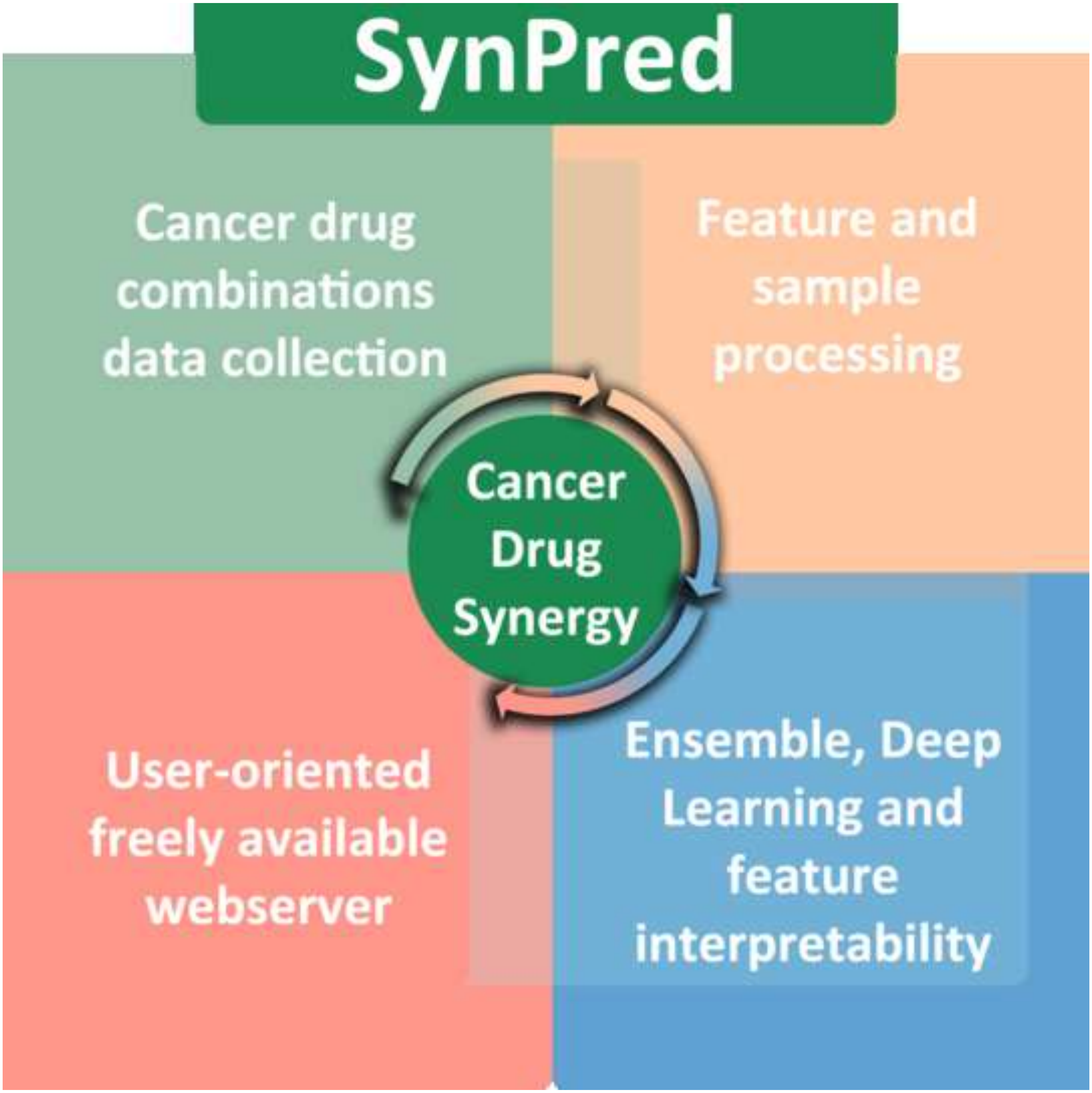

Click here to access/download;Figure;Figure1.png

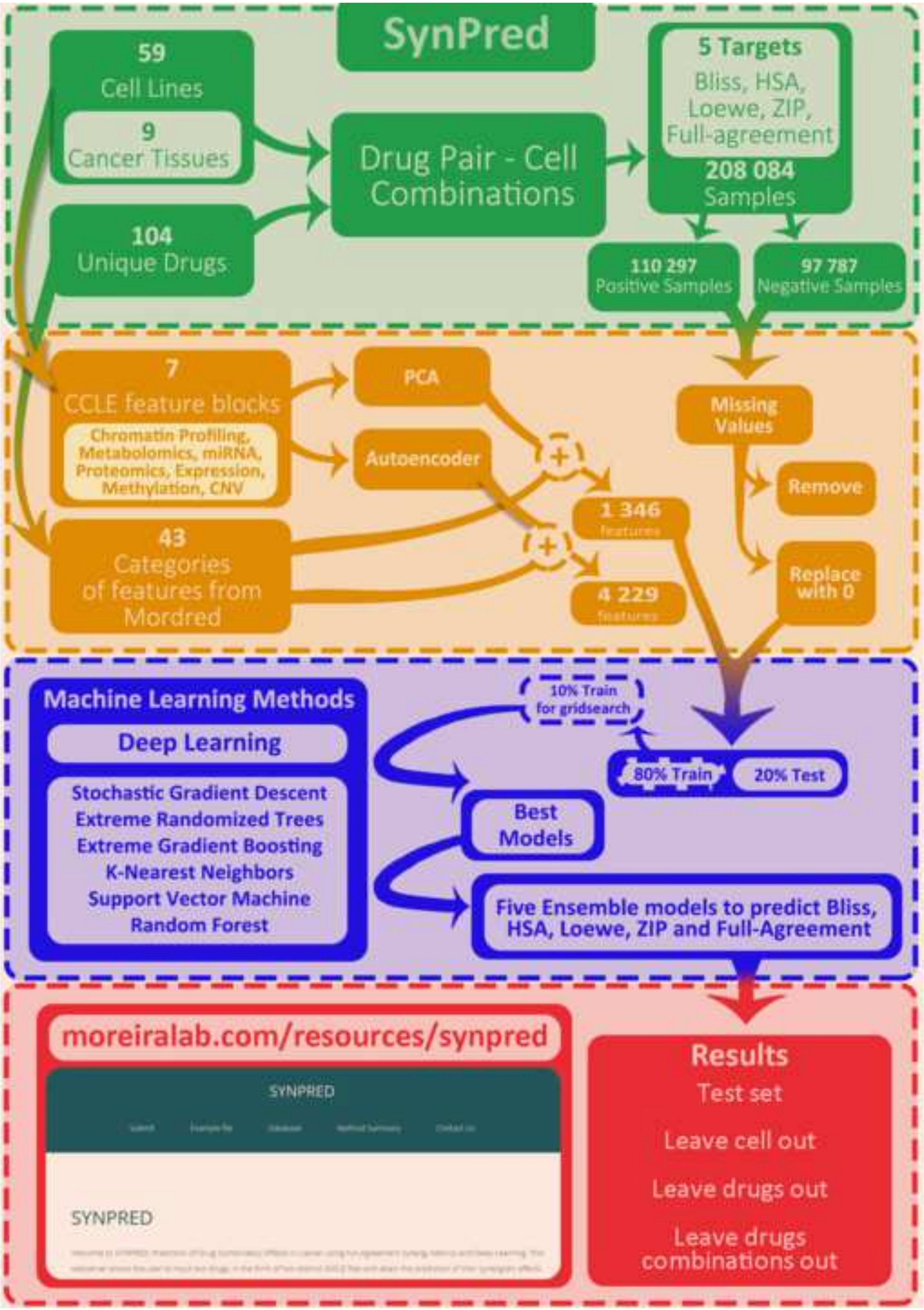

Click here to access/download;Figure;Figure2.png

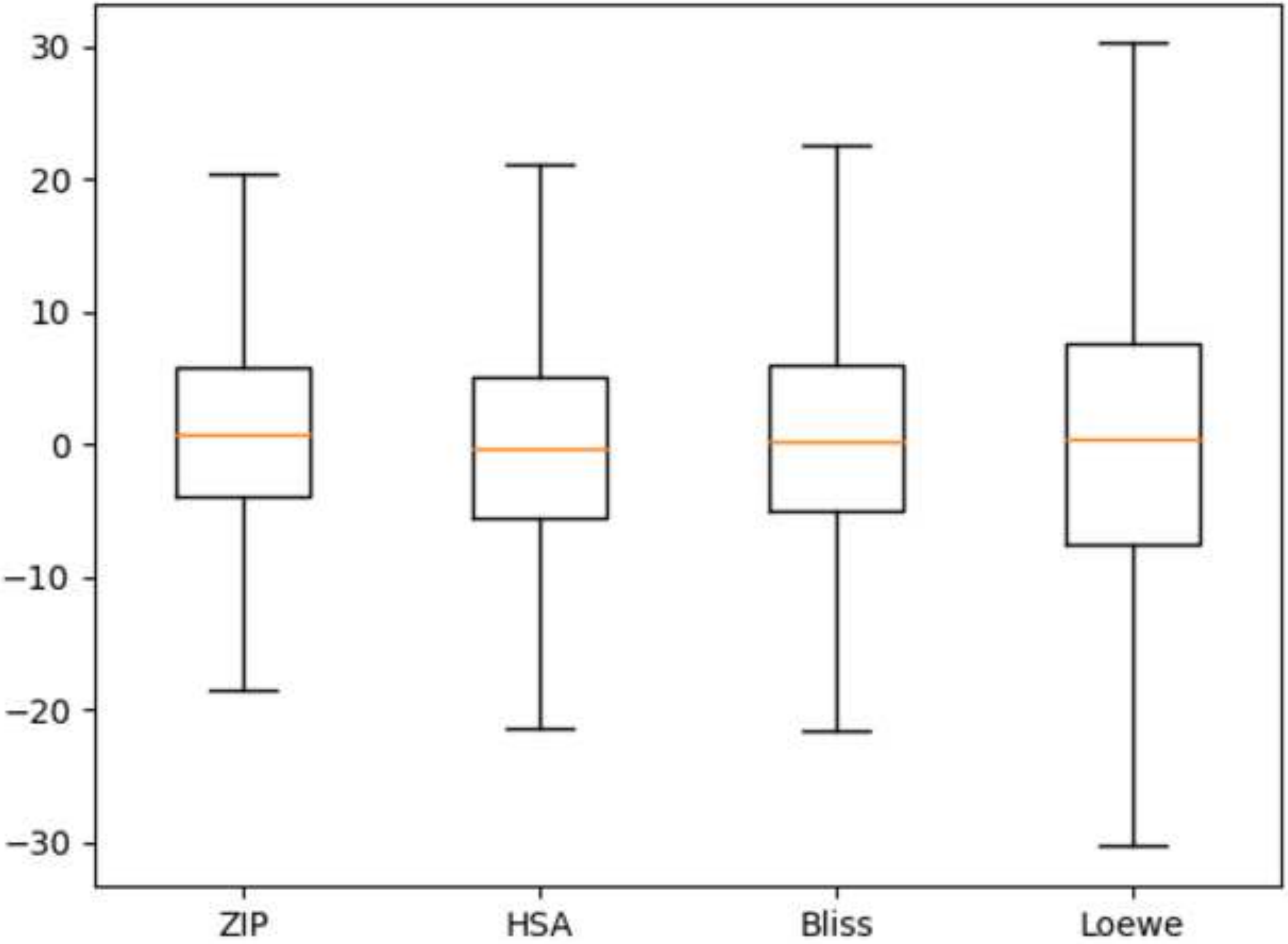

Click here to access/download;Figure;Figure3.png

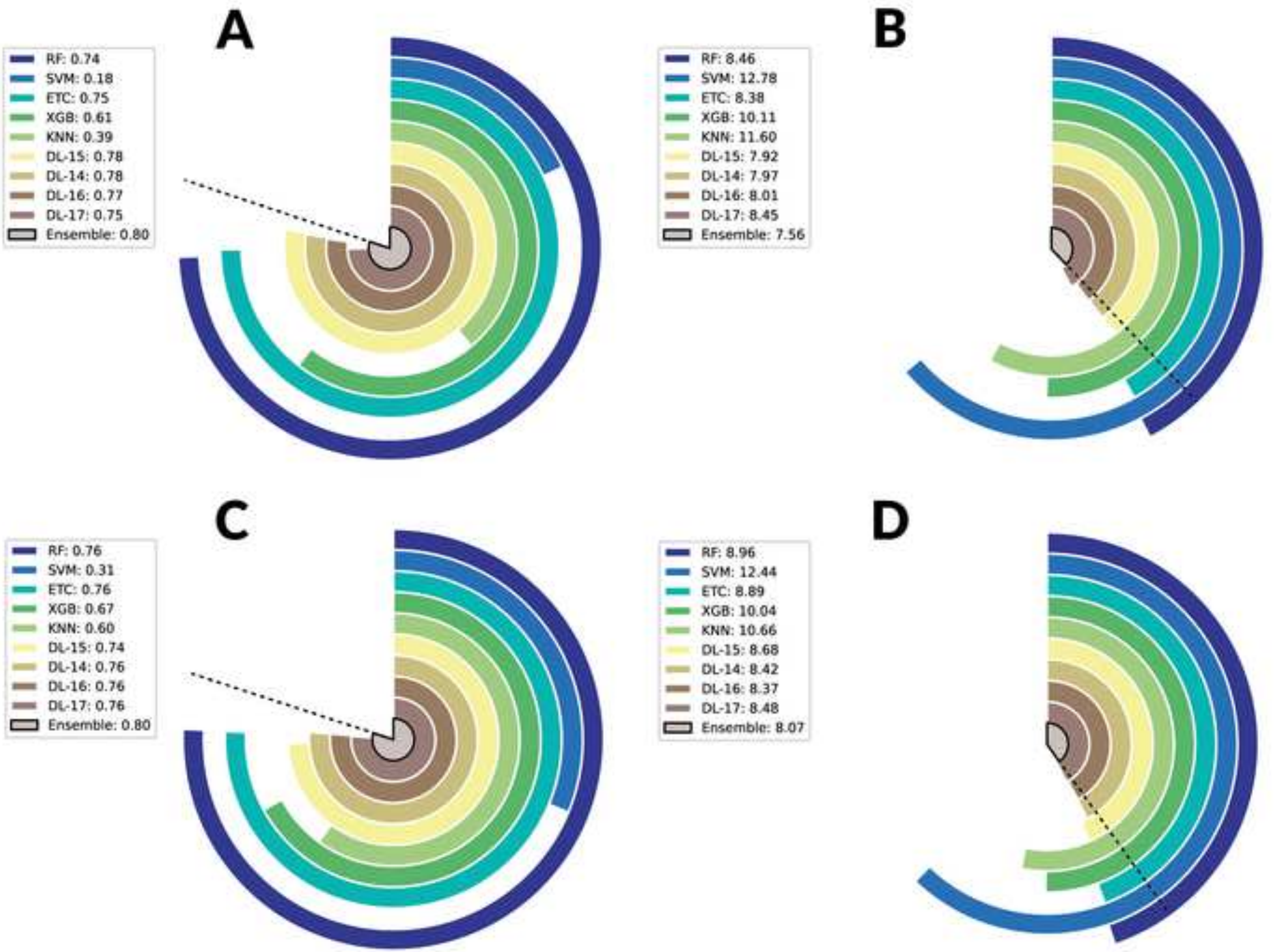

Click here to access/download;Figure;Figure4.png

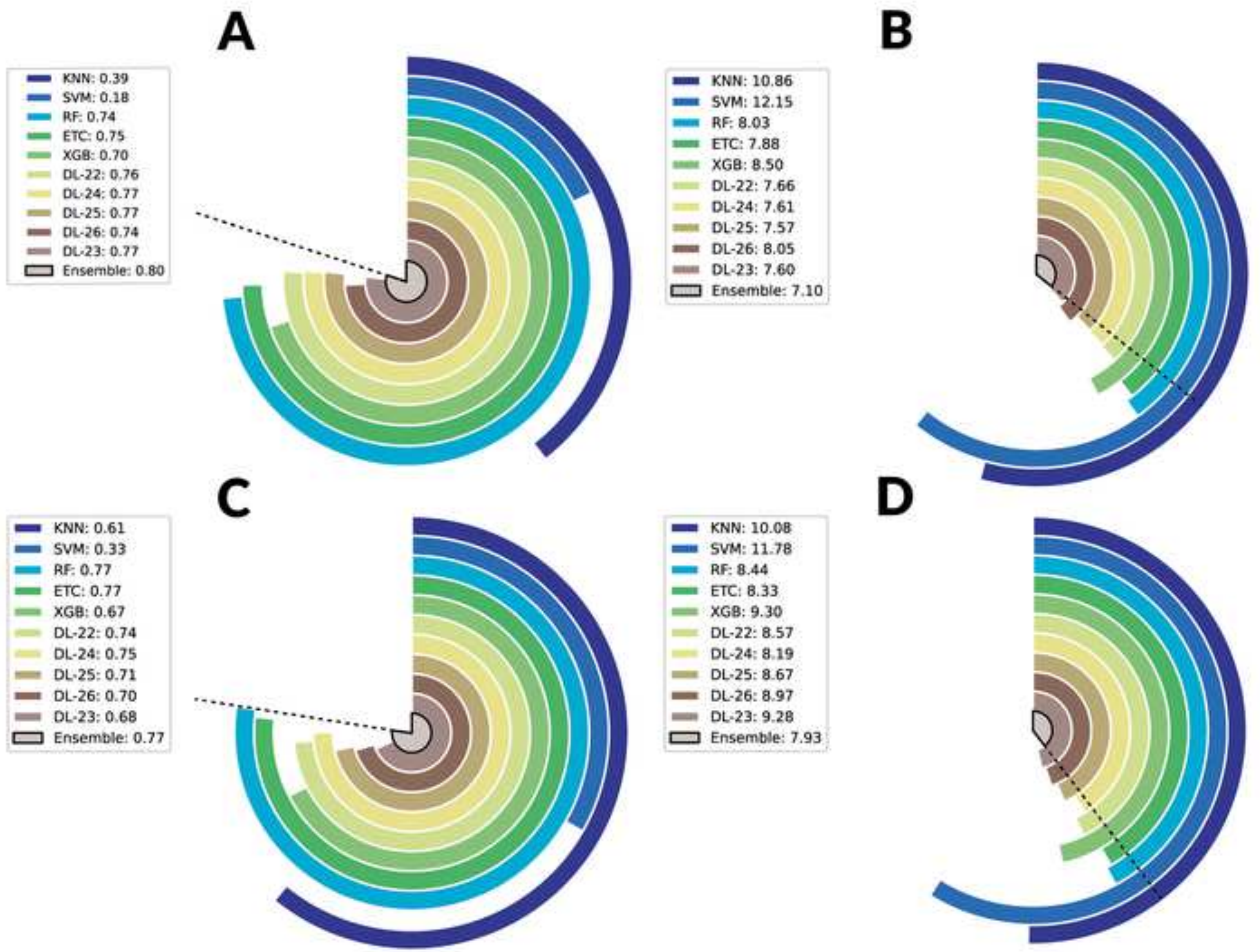

SI

Click here to access/download
Supplementary Material
SynPred_SI_GIGA-D-21-00121.docx

Cover_letter_GIGA_d_21_00121 Click here to access/download;Personal Cover;Cover_Lettter_GIGA-D-21-00121.docx

Coimbra, 17th December 2021

RE: Manuscript ID No.: GIGA-D-21-00121

Dear Prof. Hans Zauner,

Thank you for handling our manuscript. Following your email from June 10th with an invitation for a new submission upon addressing the concerns of the reviewers, we are now resubmitting our manuscript to be considered to GigaScience.

We would also like to thank the reviewers for the constructive comments on the manuscript, which were determinant for its improvement. We believe that the present version of the manuscript successfully addresses their concerns. All changes to the manuscript are marked in yellow. The answers to the reviewer's specific comments are included below. We hope that our manuscript "SYNPRED: Prediction of Drug Combination Effects in Cancer using Different Synergy Metrics and Ensemble Learning" fulfils now the necessary quality criteria to be published at GigaScience.

Yours sincerely,
Irina S. Moreira, Ph.D.

REVIEWER 1:

In this study, the authors propose a model called SynPred, based on ensemble learning for the synergy prediction problem using drugs' chemical features. They compared their ensemble learning performance with single learners and showed improvements compared to the state of the art. I have the following comments and concerns.

Major Concerns

Q1: *NCI-Almanac data is used in this model, and authors calculated Bliss, Loewe, HSA, and ZIP synergy scores from dose-response matrices. However, NCI-Almanac data has already provided a synergy score called ComboScore. I wonder why authors did not use the original synergy metric of the dataset rather than calculating other metrics. Authors should consider using ComboScore directly to develop their model.*

A: We thank the reviewer for pointing this out. First, we chose to use Bliss, Loewe, HSA, and ZIP, as these are four of the most well-documented and validated synergy reference models. With these models, it is possible to average the synergy values of different concentration pairs, while we could not find any documentation for using the same approach with ComboScore, reason why we chose not to use it. We

have clarified why ComboScore is not included in the Material and Methods section of the manuscript (please see lines 131-141).

Q2: *Authors model the synergy prediction task as a classification problem. However, all the metrics they consider (Loewe, HSA, etc.) are continuous. Authors binarize these metrics and then apply a full-agreement condition. Both discretization and the full-agreement property decrease the complexity of the problem and the task is much easier than the tasks tackled by the referenced models such as DeepSynergy, AuDNNSynergy, and MatchMaker, and the results could be over-optimistic. Authors should train a model from literature for their task, they may at least convert one of these models into a classification model and train them with hyperparameter tuning. Following the previous comment, authors should assess the performance of SynPred in the regression setting. They may use one of the synergy scores (i.e. Loewe score) and build a SynPred model that is trained for regression task. Thus, they can compare their performance with the referenced models.*

A: We thank the reviewer for the relevant comment and the corresponding suggestion that significantly improved the scientific contribution of the work. We have restructured the whole pipeline and corresponding manuscript to include the Full-agreement classification predictor plus regression predictors corresponding to the four synergy reference models (Bliss, HSA, Loewe, and ZIP), developed with fully independent hyperparameter tuning. We decided to include all the four synergy reference models to address which metrics should be considered when developing this type of regression model. Although significantly increasing the challenge of the task, this approach also proved to be incredibly beneficial under the scope of achieving an unbiased predictor. Furthermore, we now display the results of the most used individual predictors (XGB, ETC, kNN, RF, SGD, SVM and DL) to compare with the final ensemble models (please see lines 398-422 of the revised manuscript).

Q3: *Test and train splits of the model are purely based on addressing the class imbalance. Authors must consider different split schemes such as drug-combinations-out, drugs-out, and cell-lines-out splitting schemes which are mentioned in the DeepSynergy paper. Splitting just based on class imbalance gives over-optimistic results since a drug pair X-Y can be included in both train and test datasets with different cell lines.*

A: We greatly acknowledge the reviewer for a valuable suggestion. We have added drug-combinations-out, drugs-out, and cell-lines-out splits (please see lines 246-263 of the revised manuscript).

Q4: *Authors stated that they normalized data with zero mean and unit variance (lines 155 and 156). However, the authors normalize data without considering train and test splits. This may lead to information leakage between train and test splits.*

A: Previously, we had only normalized the drug-related data considering the train and test splits. However, due to high memory usage requirements, this same process was not applied to the OMICs

data. Upon the reviewer's suggestion, we have now also applied the normalization of the OMICs data under the same principles, as such avoiding leakage between train and test splits.

Q5: *On page 12, the authors analyzed the top genes in general. But, this analysis should be done for each tissue, and tissue-specific top genes can be found. This analysis makes sense if the reported tissue-specific top genes are related to cancer related to the given tissue.*

A: Although we understand the reviewer's suggestion, our focus was not to discover new biomarkers but instead only to assess the most contributing genes towards the final synergy prediction models associated with cancer prognosis, diagnosis, progression, or treatment. As such, we decided to leave this analysis as it is, and we hope to have now better clarified our aim.

Q6: *Authors compare their results with the models in the literature, such as the ones in DeepSynergy and AuDNNSynergy. However, they directly take these performance results from the corresponding papers. Both datasets and the features are different in those studies. Therefore, it is not meaningful to compare their results. Further, these studies solve the regression problem while SynPred deals with classification task. Authors need to compare their models with the literature by running referenced models.*

A: We thank the reviewer for pointing this out. However, when addressing this problem, we realized that the appropriate benchmark with other prediction models was not viable for several reasons. Many of the works do not make available the predictors so that it is possible to redeploy them adequately. Some of them do not make the code available. Although making part of the code available to replicate the experiment, others do not display the whole pipeline, for instance, regarding feature extraction and selection. The partial implementation of the models would lead to skewed results. Finally, the span of works regarding cancer synergy drugs combinations is also hindered by the usage of the dataset, particularly regarding the OMICs part. Since the source of OMICs data used (CCLE) is not available for all cell lines, this significantly narrows down comparison with other works. However, we understand the value and point of the reviewer. So, we have now highlighted the performance of individual ML models frequently used in this type of task and compared it with their final ensemble models. Please check lines 377-436 of the revised manuscript.

Q7: *In benchmarking with DECREASE section, they said that "When calculating the class with our strict full-agreement requirements, all the possible samples turned out to be negative, ...". I do not understand how and why they make predictions when all the labels are negative. Please clarify.*

A: We understand the reviewer's point. However, this was the only available dataset (apart from NCI-ALMANAC) that fulfilled all the requirements for determining synergy reference model values, as explained in the manuscript (please see lines 322-332). This dataset was also used to attain a reliable regression model. Although the samples are originally non-synergistic, the good performance of the

regression models was also displayed (particularly when using the ZIP reference model). This suggests that, although we were handicapped by only having been able to benchmark a dataset with non-synergistic samples, our models were not only able to correctly predict the samples as non-synergistic, but also within close range of the actual continuous value (please see lines 437-446).

Q8: *Authors should discuss the novelty of their model. What is the novel part of SynPred, rather than ensemble and aggregate the individual models' output compared to others?.*

A: After the reviewer's suggestions, the authors now emphasized the novelty of the work in the "Conclusions" section. We now empirically address the importance of using an appropriate synergy reference model by developing individual predictors for the more commonly addressed in the literature. This approach allows a previously unseen assessment of the behaviour of each synergy reference model under similar circumstances. Under the scope of usability, this is now embodied by a new output in SYNPRED webserver, "Synergy Votes", that displays the tallied result of synergy prediction between five independently trained predictors. Besides, we provide the complete workflow for a standalone deployment in the GitHub coupled with a freely available and easy-to-use webserver (http://www.moreiralab.com/resources/synpred/) that only requires two drugs' SMILES as inputs, thus alleviating the need of uploading a conventional and laborious dose-response matrix. Please check lines 475-480 and 484-495 of the revised manuscript.

Minor Concerns and Suggestions

Q9: *The authors explain parameter tuning on page 9 (starting from line 182). However, they do not explain the models until that point. Explaining parameter tuning before presenting models is confusing. Further, authors always using the term 'Deep Learning' for their neural network model. They should name the model correctly based on the architecture.*

A: Following the reviewer suggestion, we have now begun by presenting the models ("Development of Machine Learning Models – Neural Networks with Keras", "Development of Machine Learning Models – Machine Learning algorithms with scikit-learn") and, afterwards, parameter optimization (Development of Machine Learning Models – parameter optimization"). Regarding the second reviewer suggestion, we have labelled all the DL-based models (with more than one hidden layer) according to the architecture in Tables 7-11_Supplementary Information. Please check SI and lines 282-297 of the revised manuscript.

Q10: *The authors stated that they used permutation importance, but they do not explain or cite the permutation importance. Please explain.*

A: In compliance with the reviewer's suggestion, this information is now within the main text. Please check line 317 of the revised manuscript.

Q11: *Figure 2 is problematic since it does not include any axis labels. Authors must give the axis labels and explain what this figure tells us in the caption. Further, the colors and text font sizes are not appropriate. It is difficult to read.*

A: The authors agree with the reviewer on this subject. As such, we decided to take out this figure of the main text and redirect the reader to the dynamic version on the website, which has, upon hovering, all the information required. In compliance with this suggestion, the authors have labelled all the website figures (e.g., Figure W). By taking this approach, the value of the dynamic visualization was maximized.

Q12: *Table 3 includes training results which is unnecessary.*

A: We have now removed the training results from the manuscript as suggested.

Q13: *The performance comparisons should be more effective. Authors give nearly all the results in text. It makes it very hard to follow and compare the performances. I think that illustration of results with* ***some figures*** *can be better.*

A: Following the reviewer´s suggestion we have now developed circular bar plots (Figures 3 and 4) to display the most relevant results. Due to the massive number of results, the remaining ones are shown in Table 7-11_Supplementary Material. Please check SI and pages 21 and 22 of the revised manuscript.

<u>REVIEWER 2:</u>

In this paper, the authors propose a drug combination prediction model that ensembles AI algorithms using omics and biophysical traits. Due to lack of accessibility and usability of available tools, the authors have their models made available via a user-friendly web interface. The authors trained their models on the NCI-ALMANAC dataset which contains 311,466 drug pairs-cell line combinations spanning 104 unique drugs and 59 unique cell lines. They trained a binary classifier to identify synergistic and non-synergistic combinations by taking full agreement classes between the four synergy scoring metrics (i.e. Bliss, Loewe, Highest Single Agent and Zero Interaction Potency) The model validated on independent test set from the Ianevski study achieved a high accuracy (0.98). The paper makes a few contributions such as a methodology combining multiple synergy metrics, comparison of DL and non-DL state-of-the-art approaches to predict drug synergy combinations, and a web interface for researchers to explore drug combination prediction for the two drugs submitted. I have only a few major concerns:

Major Concerns

Q1: *It is unclear why the cell line features have a lower contribution for non-DL-based models. The authors should elaborate more on this. An ablation study may be helpful to add.*

A: We would like to thank the reviewer for taking an interest in this topic of our work. The changes were performed on the protocol and manuscript in compliance with reviewers' suggestions. We have now shown that some non-DL-based models can often make as much use of the cell line features as the DL-based ones. However, DL-based models still make a more extensive use. The suggestion of ablation study performance, however valuable, is addressed by the chosen method itself. By calculating Permutation Importance, the randomization of each of the columns' values ensures that its contribution is assessed as if it was not present and, instead, only the remaining features were contributing to the prediction. We have now clarified in the main text. Please lines 316-321 and 340-366 of the revised manuscript.

Q2: *What are the top combination predictions from the DL model and can you validate the model's top predictions?*

A: Following the reviewers' suggestion, we have now included drug-combinations-out, drugs-out, and cell-lines-out splits. When deploying the trained predictors on these splits, it was shown that most regression predictors' performance on the drug-combinations-out was maintained, thus enforcing the model's strength on unseen drug combinations. Please lines 246-263, table 4 and figures 3 and 4 of the revised manuscript.
