# Supplementary material for: SYNPRED: prediction of drug combination effects in cancer using different synergy metrics and ensemble learning
Source: Gigascience. 2022 Sep 26;11:giac087. doi: 10.1093/gigascience/giac087 (PMC9511701; doi:10.1093/gigascience/giac087)
Supplement: giac087_GIGA-D-21-00416_Revision_2 [file giac087_giga-d-21-00416_revision_2.pdf]

GigaScience

SYNPRED: Prediction of Drug Combination Effects in Cancer using Different Synergy Metrics and Ensemble Learning

--Manuscript Draft--

**Manuscript Number:**	GIGA-D-21-00416R2
**Full Title:**	SYNPRED: Prediction of Drug Combination Effects in Cancer using Different Synergy Metrics and Ensemble Learning
**Article Type:**	Technical Note
**Funding Information:**	Fundação para a Ciência e a Tecnologia (LA/P/0058/2020) — Prof. Irina Sousa Moreira<br>Fundação para a Ciência e a Tecnologia (POCI-01-0145-FEDER-031356) — Prof. Irina Sousa Moreira<br>Fundação para a Ciência e a Tecnologia (DSAIPA/DS/0118/2020) — Prof. Irina Sousa Moreira<br>Fundação para a Ciência e a Tecnologia (SFRH/BD/144966/2019) — Mr António J. Preto
**Abstract:**	Background: In cancer research, high-throughput screening technologies produce large amounts of multiomics data from different populations and cell types. However, analysis of such data encounters difficulties due to disease heterogeneity, further exacerbated by human biological complexity and genomic variability. The specific profile of cancer as a disease (or, more realistically, a set of diseases) urges the development of approaches that maximize the effect while minimizing the dosage of drugs. Now is the time to redefine the approach to drug discovery, bringing an Artificial Intelligence-powered informational view that integrates the relevant scientific fields and explores new territories.<br>Results: Here, we show SYNPRED, an interdisciplinary approach that leverages specifically designed ensembles of AI algorithms, links omics and biophysical traits to predict anticancer drug synergy. It uses five reference models (Bliss, Highest Single Agent, Loewe, Zero Interaction Potency and Combination Sensitivity Score), which, coupled with AI algorithms, allowed us to attain the ones with the best predictive performance and pinpoint the most appropriate reference model for synergy prediction, often overlooked in similar studies. By using an independent test set, SYNPRED exhibits state-of-the-art performance metrics either in the classification (accuracy – 0.85, precision – 0.91, recall – 0.90, AUROC – 0.80, and F1-score - 0.91) or in the regression models, mainly when using the Combination Sensitivity Score synergy reference model (RMSE – 11.07, MSE – 122.61, Pearson – 0.86, MAE – 7.43, Spearman – 0.87). Moreover, data interpretability was achieved by deploying the most current and robust feature importance approaches. A simple web-based application was constructed, allowing easy access by non-expert researchers .<br>Conclusions: The performance of SYNPRED rivals that of the existing methods that tackle the same problem, yielding unbiased results trained with one of the most comprehensive datasets available (NCI-ALMANAC). The leveraging of different reference models allowed deeper insights into which of them can be more appropriately used for synergy prediction. The Combination Sensitivity Score clearly stood out with improved performance among the full scope of surveyed approaches and synergy reference models. Furthermore, SYNPRED takes a particular focus on data interpretability, which has been in the spotlight lately when using the most advanced AI techniques.
**Corresponding Author:**	Irina Sousa Moreira<br>University of Coimbra: Universidade de Coimbra<br>Coimbra, US and Canada only PORTUGAL
**Corresponding Author Secondary Information:**	
**Corresponding Author's Institution:**	University of Coimbra: Universidade de Coimbra
**Corresponding Author's Secondary Institution:**	

Powered by Editorial Manager® and ProduXion Manager® from Aries Systems Corporation

**First Author:**	António J. Preto
**First Author Secondary Information:**	
**Order of Authors:**	António J. Preto
	Pedro Matos-Filipe
	Joana Mourão
	Irina Sousa Moreira
**Order of Authors Secondary Information:**	
**Response to Reviewers:**	Coimbra, 2h August 2022<br>RE: Manuscript ID No.: GIGA-D-21-00121<br><br>Dear Prof. Hans Zauner,<br>Thank you for handling our manuscript throughout this process.<br>All changes to the manuscript are marked in red. The answers to the reviewer's specific comments are included below. We hope that our manuscript SYNPRED: Prediction of Drug Combination Effects in Cancer using Different Synergy Metrics and Ensemble Learning" fulfils now the necessary quality criteria to be published at GigaScience.<br>Yours sincerely,<br>Irina S. Moreira, Ph.D.<br><br>Reviewer #1: Authors indicate that benchmarking of synergy prediction protocols is a complicated process. While I agree with this. , I do not agree that "..DL architectures as these are not easily applied or not available in GitHub or similar platforms." As the implementations are available on GitHub, the authors can feed their data of choice to easily train these models. There might be some adaptation choices to make such as pruning the data due to unavailability of a certain data modality, or changing the loss function to turn a model into a regressor, but as long as they justify these it should be fine and east. This is what has been done in the literature in DeepSynergy and Matchmaker papers, for instance. They have done this to a degree but I am not convinced that a fair comparison with the literature I made to conclude that this method has clear advantages over others. Authors indicate that they trained 1972 models but I think these are mostly the "baseline" models they use within their ensemble method which does not help in comparing with the literature. Below, I list my detailed comments on the revisions made:<br>First, while there are many detailed descriptive statistics such as distribution of synergy scores are presented in the main text the comparisons are pushed to the supplement which makes it hard to follow the paper. Also, details on whether or how compared methods are run/trained is missing. For instance in comparison ii), how did you reimplement DeepSynergy, and in Comparison ii) and iv) what data did you use and what hyperparameters did you use to run DeepSynergy and Matchmaker? In the current revision, in Tables S7 - S12 authors compare their method with some baseline models used in their ensemble such as random forest, KNN and some custom deep learning architectures. I did not ask for this as clearly the problem is complex and a complex model as their method is needed. Nevertheless this is a nice validation which improves the paper. They compare their method with a reimplemented (please clarify in text) version of DeepSynergy in Table S13 (They say Table S14 but I think it is a typo).<br>We have corrected the typo and changed it to Table 13_Supplementary Material.<br>As thoroughly discussed, there is a fundamental disagreement between the authors' and the reviewer on this matter. A correct benchmark on this problem is not currently feasible, as we have attempted to make it clear to the reviewer. The benchmarking problem is evidenced by the publication of ChemicalX (Rozemberczki et al. 2022), in which DeepSynergy, MatchMaker, among others algorithm are re-implemented. This work was published in 2022 by several researchers. The fact that they had to re-implement the algorithms shows what we have stated for several versions of the answers to the reviewer: the present state of the art does not allow for proper benchmark. Unfortunately, ChemicalX only provides a binary class for drug synergy prediction.<br>However, we deployed a multi-step approach that compares individually either datasets, Machine Learning deployments or literature referenced results. We indeed

Powered by Editorial Manager® and ProduXion Manager® from Aries Systems Corporation

	benchmarked our pipeline as described in the methods section:<br>i) Comparison of the best performing individual DL and ML algorithms with the ensemble approaches for each prediction task - Tables 7 to 12_Supplementary Material.<br>ii) DeepSynergy architecture implementation and comparison using our independent test set and validation sets - Table 13_Supplementary Material.<br>iii) Comparison with published methods for synergy calculations as reviewed by Kumar et al. - Table 14_Supplementary Material.<br>iv) Comparison of our regression approaches to Matchmakers' algorithm, DeepSynergy and TreeCombo - Tables 15 and 16_Supplementary Material.<br><br>First, I strongly suggest they put the result of the final method into this table (and other related ones) for the readability. I had to trace Table 4/5 to see their results and come back to compare with the values in this table. They report better results than DeepSynergy which is good. As they use the review of Kumar et al. later, there are other methods reported in this review which perform better than DeepSynergy in the regression task such as MultitaskDNN, AUDNNSynergy, and Matchmaker. However only DeepSynergy is compared. Later they perform a comparison with Matchmaker but it is ot clear to me why they had to perform a separate comparison instead of comparing them all. In supplementary table 14, they compare the performance of their method, with performance values of others reported in a review by Kumar et al. This is not meaningful at all as every method use different settings as authors also indicate. Once again, they have to retrain these (or similar) architectures with their (maybe filtered or postprocessed) data and compare. Just like done for DeepSynergy only in the regression task. They can as well retrain their method with the dataset of these methods as another point of view. For the results in Tables S15 - S16 (again the table numbers are wrong), author state "... Upon doing this, both CSS and Loewe predictors from SynPred stood very close to the performance of Matchmaker [20],". Matchmaker reports Loewe scores so if no retraining of the model is performed with CSS it is not meaningful to compare these results. I suggest authors to use the CSS scores as the label in training Matchmaker. In Loewe scores their performance actually does not stand vey close to Matchmaker (MSE vs 123, Pearson .79 vs .73). Only in Spearman correlation their result is 0.01 better. In Table S16, SynPred seems to be 50 times better than all others in terms of MSE. However, while SynPred reports various scores other than ComboScore, the results obtained for other methods are based on ComboScore. Thus, it is comparing apples and oranges and is not meaningful. Again, no training is performed in this comparison. Please do not put results with different metrics into the same table as it is very confusing for the reader.<br>The complexity of the benchmark problem is thoroughly addressed throughout the manuscript, and it cannot be reduced to a method against method comparison.<br>We have reimplemented DeepSynergy the same way the other authors that claim do benchmark DeepSynergy did, by reimplementing a neural network with the same dimensions and hyperparameters although on other data. We also compared the results of our predictor on Matchmakers' dataset and the results attained by these authors. The clarifications regarding implementation are now in page 16 of the revised manuscript.<br>Table S16 displays several predictors, many of which do not have ComboScore as target. We clearly state which SynPred predictors we are referring to by adding the name of target variable in front of SynPred.<br>We did expand our comments MSE, precisely because we are aware that error derived metrics are particularly hard to compare between datasets and methods. Clarified in page 26 of the revised manuscript.<br>We do not believe that adding table 4 and 5 in some of supplementary tables will increase readability, just the opposite and we did not follow this suggestion.<br>We have changed the numbering of tables as indeed these were incorrect.<br><br>The details on the dataset used, the train/validation/test splits and number of samples etc. are missing so it is hard to evaluate these scores.<br>This statement is purely wrong, all requested data is available on Table 3_Supplementary Material and detailed in text (subsection "Model evaluation and performance metrics", lines 252-276).<br><br>TYPOs: NCI ALMANAC, Matchmakers'<br>We have followed reviewer advice and changed the two types: NCI-ALMANC towards

	NCI ALMANAC and Matchmaker to Matchmakers' in the main document as well as in SI.
**Additional Information:**	
**Question**	**Response**
Are you submitting this manuscript to a special series or article collection?	No
**Experimental design and statistics**<br><br>Full details of the experimental design and statistical methods used should be given in the Methods section, as detailed in our Minimum Standards Reporting Checklist. Information essential to interpreting the data presented should be made available in the figure legends.<br><br>Have you included all the information requested in your manuscript?	Yes
**Resources**<br><br>A description of all resources used, including antibodies, cell lines, animals and software tools, with enough information to allow them to be uniquely identified, should be included in the Methods section. Authors are strongly encouraged to cite Research Resource Identifiers (RRIDs) for antibodies, model organisms and tools, where possible.<br><br>Have you included the information requested as detailed in our Minimum Standards Reporting Checklist?	Yes
**Availability of data and materials**<br><br>All datasets and code on which the conclusions of the paper rely must be either included in your submission or deposited in publicly available repositories (where available and ethically appropriate), referencing such data using a unique identifier in the references and in the “Availability of Data and Materials”	Yes

section of your manuscript.<br><br>Have you have met the above requirement as detailed in our Minimum Standards Reporting Checklist?	

Powered by Editorial Manager® and ProduXion Manager® from Aries Systems Corporation

Manuscript

Click here to access/download;Manuscript;Synpred-R3_for_production.docx

SYNPRED: Prediction of Drug Combination Effects in Cancer using Different Synergy Metrics and Ensemble Learning

António J. Preto[1,2], Pedro Matos-Filipe[1], Joana Mourão[1] and Irina S. Moreira[3,4*]

[1]University of Coimbra, Center for Neuroscience and Cell Biology, 3004-504 Coimbra, Portugal

[2]PhD Programme in Experimental Biology and Biomedicine, Institute for Interdisciplinary Research (IIIUC), University of Coimbra, Casa Costa Alemão, 3030-789 Coimbra, Portugal

[3]University of Coimbra, Department of Life Sciences, Calçada Martim de Freitas, 3000-456 Coimbra, Portugal

[4]CNC - Center for Neuroscience and Cell Biology, CIBB - Center for Innovative Biomedicine and Biotechnology, 3004-504 Coimbra, Portugal

ORCID iDs: António J Preto [0000-0003-4203-2230]; Pedro Matos-Filipe [0000-0003-0492-0683]; Joana Mourão [0000-0002-1296-5964]; Irina Sousa Moreira [0000-0003-2970-5250];

* To whom correspondence should be addressed.

Tel: (+351) 239 240 227

Email: irina.moreira@cnc.uc.pt

ABSTRACT

Background: In cancer research, high-throughput screening technologies produce large amounts of multiomics data from different populations and cell types. However, analysis of such data encounters difficulties due to disease heterogeneity, further exacerbated by human biological complexity and genomic variability. The specific profile of cancer as a disease (or, more realistically, a set of diseases) urges the development of approaches that maximize the effect while minimizing the dosage of drugs. Now is the time to redefine the approach to drug discovery, bringing an Artificial Intelligence-powered informational view that integrates the relevant scientific fields and explores new territories.

Results: Here, we show SYNPRED, an interdisciplinary approach that leverages specifically designed ensembles of AI algorithms, links omics and biophysical traits to predict anticancer drug synergy. It uses five reference models (Bliss, Highest Single Agent, Loewe, Zero Interaction Potency and Combination Sensitivity Score), which, coupled with AI algorithms, allowed us to attain the ones with the best predictive performance and pinpoint the most appropriate reference model for synergy prediction, often overlooked in similar studies. By using an independent test set, SYNPRED exhibits state-of-the-art performance metrics either in the classification (accuracy – 0.85, precision – 0.91, recall – 0.90, AUROC – 0.80, and F1-score - 0.91) or in the regression models, mainly when using the Combination Sensitivity Score synergy reference model (RMSE – 11.07, MSE – 122.61, Pearson – 0.86, MAE – 7.43, Spearman – 0.87). Moreover, data interpretability was achieved by deploying the most current and robust feature importance approaches. A simple web-based application was constructed, allowing easy access by non-expert researchers.

Conclusions: The performance of SYNPRED rivals that of the existing methods that tackle the same problem, yielding unbiased results trained with one of the most comprehensive datasets available (NCI ALMANAC). The leveraging of different reference models allowed deeper insights into which of them can be more appropriately used for synergy prediction. The Combination Sensitivity Score clearly stood out with improved performance among the full scope of surveyed approaches and synergy reference models. Furthermore, SYNPRED takes a particular focus on data interpretability, which has been in the spotlight lately when using the most advanced AI techniques.

KEYWORDS

Ensemble-Learning; Interpretability; Omics; Biophysics; Drug Synergy; Cancer.

BACKGROUND

Cancer, a heterogeneous group of diseases, is one of the leading causes of mortality and the most significant barrier to increasing life expectancy worldwide. The International Agency for Research on Cancer estimates that, by 2040, approximately 29.5 million new cancer cases and 16.4 million deaths will be reported, mainly due to the population's growth and ageing [1]. One of the significant contributors to this disease's global burden is the development of therapy resistance and, consequently, tumour relapse. Drug resistance in cancer is a multifactorial problem driven by the tumour microenvironment and genetic and nongenetic/epigenetic mechanisms that, along with cell plasticity, contribute to tumour heterogeneity [2]. In clinical settings, this problem is minimized with a combination of drugs administered together or in sequence, i.e., polytherapy. Targeting multiple components of different or interconnected cancer pathways is an efficient strategy to block vital biological processes [3,4].

Drug combinations with a synergistic effect, i.e., when the total therapeutic effect of both drugs is greater than the expected additive monotherapy effect [5], were successfully developed and applied in the treatment of different types of tumours, such as human epidermal growth factor receptor 2-positive breast cancer [6], chronic myeloid leukaemia [7], prostate cancer [8] or BRAF-mutant melanoma [9]. Nevertheless, this simultaneous administration can also result in a reduced therapeutic effect and possible toxicity (designated antagonism) or in the same beneficial effect when compared with the expected additive monotherapy effect (additivity) [5]. The experimental identification of successful synergistically effective combinations is a well-known time-consuming, and expensive task. Therefore, there is still a significant need for efficient and user-friendly computational methods, available in easy to use interfaces, to complement and speed-up the traditional approaches by predicting the best synergistic drug combinations [10,11].

In the last years, the development and improvement of high-throughput technologies and computational tools boosted the use of large volumes of multi-omics data (e.g., genomic, transcriptomic, proteomic) essential to dissect and uncover the complex molecular signatures of cancer. Machine Learning (ML)

algorithms have attracted particular attention for their ability to learn new associations and extract valuable insights from this type of data. A few ML models based on eXtreme Gradient Boosting, Random Forest, Elastic Nets, Support Vector Machine, and Naïve Bayes were already developed to predict the best combination of anticancer drugs by the integration of omics data with chemoinformatic properties of drugs or network information of their targets [12–15]. Likewise, Deep-Learning (DL) implemented via Deep-Neural Networks (DNNs) was particularly useful in dealing with the high multi-dimensionality of omics data in supervised and unsupervised contexts. DL classification and regression models such as AuDNNsynergy [16], DeepDDS [17], DeepSynergy [18], DeepSignalingSynergy [19], Matchmakers' [20], TranSynergy [21] or the work by Xia and colleagues [22] were recently developed for drug combination prediction. Nearly all the surveyed works developed drug synergy prediction models based upon a single reference model, which is in most cases the Loewe reference model [14,16–18,20,21]. Currently, there is a wide scope of well-studied available reference models, including the Bliss independence [23], Highest Single Agent – HSA [24], Loewe additivity [25,26] and Zero Interaction Potency – ZIP [27]. Furthermore, recently Malyutina et al. developed the Combination Sensitivity Score (CSS), which measures drug combination synergy using their IC_{50} [15]. As such, this led us to the question of whether the development of a novel prediction approach should be based solely upon a single reference model. Besides, most of the available web interfaces such as DECREASE [28] or DrugComb [29] require for synergy prediction the upload of a full or partial mandatory dose-response matrix (experimentally determined), which hinders its systematic use by the scientific community and handicaps its usefulness.

To overcome the current problems found in the field, we developed SYNPRED (SYNergy PREDiction), a collection of *in-silico* ensemble classification and regression models that considers several synergy references models: Bliss, Loewe, HSA, ZIP and CSS. It was developed by integrating multi-omics features of cell lines, phenotypic, and biophysical data, particularly physicochemical and structural features of drugs. SYNPRED displays a good predictive performance and inherently addresses the issue at a broader and more profound angle than the existing approaches, which generally focus on either classification or a single regression task and typically use a single synergy reference model. We made available the stand-alone deployment at https://github.com/MoreiraLAB/synpred, which allows the user the opportunity to undergo bulk prediction with SYNPRED. Additionally, for the first time, a user-friendly web-based application was assembled and made freely available online at

http://www.moreiralab.com/resources/synpred/ to predict drug combinations, requiring only the upload of the two drugs Simplified Molecular-Input Line-Entry System (SMILEs) to be tested. This interactive platform will allow users with different backgrounds, from scientists to clinicians, to test, reproduce and validate our models and data. The workflow used for the development of SYNPRED is depicted in **Figure 1**.

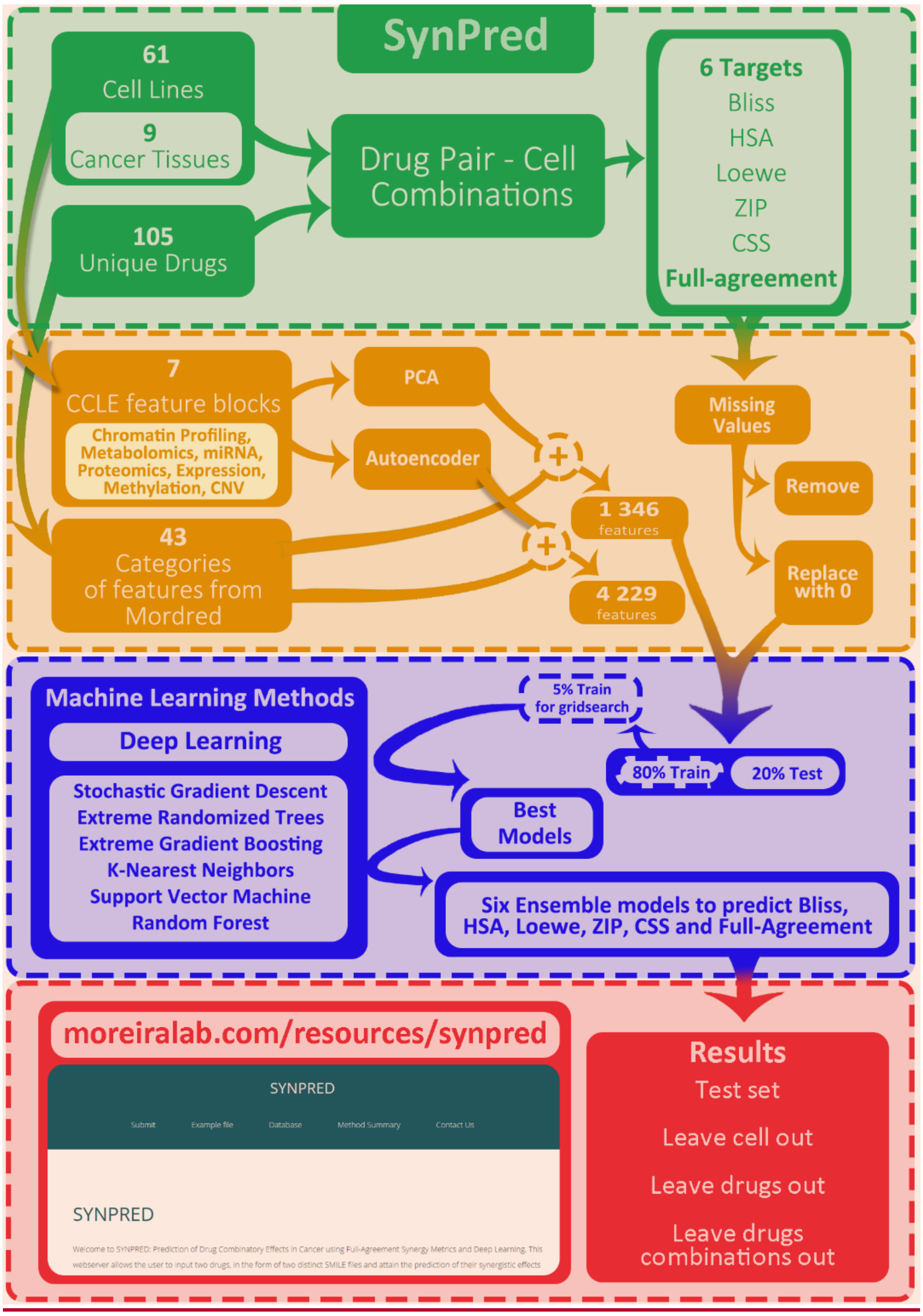

Figure 1. SYNPRED workflow summary. (Green) - Dataset construction. The National Cancer Institute - A Large Matrix of Anti-Neoplastic Agent Combinations database (phenotypic data) and the

Cancer Cell Line Encyclopedia (CCLE) (multiomics data) were used for this purpose. Four reference models (Bliss, Highest Single Agent-HSA, Loewe, Zero Interaction Potency-ZIP) in addition to the Combination Sensitivity Score-CSS were used to quantify the combination degree and retrieve a full agreement between all metrics. (Orange) – Feature extraction and data pre-processing. Included normalization and dimensionality reduction using autoencoder or Principal Component Analysis (PCA). (Blue) – Gridsearch and prediction models development using a training set. (Red) – Model evaluation using different classification and regression metrics in an independent test set and three different scenarios: i) leave cell out dataset, ii) leave drugs out dataset, iii) leave drug combinations out dataset.

DATA AND METHODS

Experimental drug combination phenotypic data. Drug combination phenotypic data was acquired via bulk-download from the largest-to-date dataset from National Cancer Institute - A Large Matrix of Anti-Neoplastic Agent Combinations (NCI ALMANAC) through https://wiki.nci.nih.gov/display/NCIDTPdata/NCI ALMANAC [30]. To this date, the dataset includes phenotypic data of tested cancer cell lines (growth percentage) of 105 unique FDA-approved drugs. These drugs were tested in combination against 61 cell lines from 9 cancer types currently included in the NCI [31,32], comprising a total of 311.466 drug pair/cell line combinations. Drug sensitivity assays included in NCI ALMANAC were performed at the NCI's Frederick National Laboratory for Cancer Research, the Stanford Research Institute, and the University of Pittsburgh. Briefly, for each assay, cells were cultivated for 48 hours in a 3x3 or a 5x3 concentration matrix (different concentration values for each drug in combination) and the endpoint determined by Sulforhodamine B or CellTiter-Glo [30]. From these records, the authors retrieved the cell growth percentage at each drug concentration point, which corresponds to the percentage of growth of the cell lines in the presence of each combination, yielding a final viability assessment.

Combination scores and class definition. The phenotypic data from high-throughput drug combination screens were retrieved from DrugComb [29]. DrugComb extends its synergy metrics calculations from "SynergyFinder" [33], which leverages the percentage of cell growth included in the dataset to assess the degree of combination for each pair of drug concentrations by using several synergy reference

models. As such, only the most well-studied synergy reference models described in the literature were included as they were the only that met the criteria of characterising the effects of a drug pair on a cell line with a final single synergy score. This approach narrowed down our options to the four most well-known synergy reference models: Bliss independence (**Equation 1**) [23], Loewe additivity (**Equation 2**) [25,26], Highest Single Agent (HSA) (**Equation 3**) [24], and Zero Interaction Potency (ZIP) (**Equation 4**) [27]. In addition to the mentioned synergy reference models, we also used CSS metric [15], a higher sensitivity score (CSS) [29].

$$yBliss = y1 + y2 - y1y2$$

Equation 1: Bliss independence model. yBliss – Bliss response; y1 – drug1 response; y2 – drug2 response.

$$yLoewe = \frac{Emin + Emax\left(\frac{x1 + x2}{m}\right)^{\lambda}}{1 + \left(\frac{x1 + x2}{m}\right)^{\lambda}}$$

Equation 2: Loewe additivity model. *yLoewe* – Loewe response; *Emin* – minimum drug response; *Emax* – maximum drug response; *m* – dose that produces midpoint effect between *Emin* and *Emax*; *λ* – shape parameter indicating the slope of the curve; *x1* – drug 1 dose; *x2* – drug 2 dose.

$$yHSA = \max(y1, y2)$$

Equation 3: Highest Single Agent (HSA) model; *yHSA* – HSA response; *y1* – drug 1 response; *y2* – drug 2 response.

$$yZIP = \frac{\left(\frac{x1}{m1}\right)^{\lambda1}}{1 + \left(\frac{x1}{m1}\right)^{\lambda1}} + \frac{\left(\frac{x2}{m2}\right)^{\lambda2}}{1 + \left(\frac{x2}{m2}\right)^{\lambda2}} - \left(\frac{\left(\frac{x1}{m1}\right)^{\lambda1}}{1 + \left(\frac{x1}{m1}\right)^{\lambda1}} * \frac{\left(\frac{x2}{m2}\right)^{\lambda2}}{1 + \left(\frac{x2}{m2}\right)^{\lambda2}}\right)$$

Equation 4: Zero Interaction Potency (ZIP) model; *yZIP* – ZIP response; *x1* – drug 1 dose; *x2* – drug 2 dose; *m1* – dose that produces midpoint effect for drug 1; *m2* – dose that produces midpoint effect for drug 2; *λ1* – shape parameter indicating the slope of the curve for drug 1; *λ2* – shape parameter indicating the slope of the curve for drug 2.

Having computed Bliss, HSA, Loewe, ZIP and CSS, a binary classifier was first developed to identify the type of combinatory effect present in each drug pair-cell line sample, where the values above the threshold (0, as defined for each metric by SynergyFinder [33]) (https://synergyfinder.fimm.fi/synergy/synfin_docs/) were defined as synergistic, and the remaining ones were classified as non-synergistic. The dataset used for classification training considered full-agreement combination assessment, i.e., we only kept the instances on which combination classification was the same across the four previous reference predictors. For the dataset used, this process yielded 29.779 synergistic samples and 9.029 non-synergistic samples. For the regression model deployment, we used the values attained directly from DrugComb to each synergy reference model (Bliss, HSA, Loewe, ZIP) as well as CSS. Most synergy reference model values were in similar scales (Loewe = [-116.63, 86.69], ZIP = [-36.08,66.66], HSA = [-81.75,64.29], Bliss = [-77.07,78.65]) (**Figure 2, Figures W1-W6** of the SYNPRED webserver). CSS stood in the interval [-54.05,99.84], albeit with larger interquartile distances than the synergy reference models.

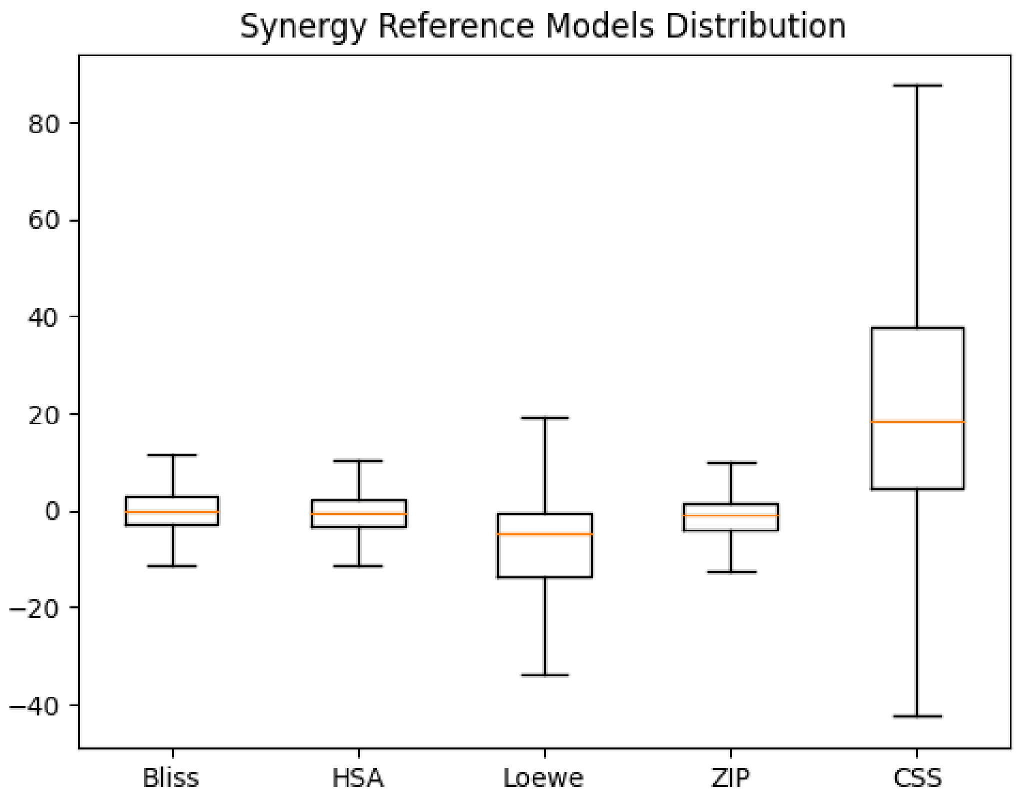

Figure 2. Box plot representing the distribution of synergy scores (y-axis) in respect to the five reference models: Bliss, HSA, Loewe, ZIP and CSS (x-axis). The black boxes represent the difference between the upper 75% and the lower 25% quartiles (interquartile range); the horizontal orange line is the median; the whiskers are the lower and upper values that are not outliers or extremes (not represented as some of these values are off range).

Drug molecular descriptors. Each drug included in NCI ALMANAC was analysed to extract its physicochemical and structural features. A SMILE representation of the drugs was acquired from PubChem [34]. SMILEs were then used to mine molecular descriptors using the Python package "Mordred" (version 1.1.2) [35]. In total, was retrieved an array of 1.613 numeric features of 43 different categories making a two-dimensional molecular description of the drugs. Feature-arrays comprising non-numerical attributes or displaying zero variance were deleted. This pre-processing left 586 features describing each drug included in NCI ALMANAC, distributed across 28 categories (**Table 1**). The resulting features were subjected to normalisation by removing the mean and scaling to unit variance with scikit-learn's StandardScaler [36].

Table 1. Number of features according to the molecular descriptor category of Mordred. Features are categorized as Energetic (E), Pharmacological (P), Structural (S) or Miscellaneous (M - in case of evaluating characteristics of multiple fields).

**Number of Features per Descriptor Category**					
E	Acidity/Basicity	2	S	Information Content	36
P	ADME	3	S	Molecular Complexity	1
S	Aromatics	2	P	Molecular Operating Environment	51
S	Atom Count	16	S	Molecule Graph	5
S	Atom-bond Connectivity	2	S	Path Count	21
M	Autocorrelation	180	E	Polarizability	2
S	Bond Count	9	S	Ring Count	66
E	Atomic Orbitals	10	S	Rotatable Bonds	1

S	Chirality	38	S	Topological Charges	21
S	Constitutional	14	S	Topological Index	7
E	Energy State	68	S	Topological Polar Surface Area	2
S	Fragment Complexity	1	S	Walk Counts	21
S	Framework	1	S	Weight	2
S	Hydrogen Bonds	2	M	Wildman-Crippen	2

Omics data of cancer cell lines. Omics data (expression, copy number variation, and methylation, global chromatin profiling, metabolomics, microRNA, proteomic profiling) describing the cancer cell lines were acquired via bulk download from the Cancer Cell Line Encyclopedia - CCLE (https://sites.broadinstitute.org/ccle/) [37]. The number of cell lines included in CCLE varies depending on the type of omics data available at the time. Correspondence of cell line IDs between NCI ALMANAC and CCLE was performed according to data available at the Swiss Institute of Bioinformatics Cellosaurus Website [38]. According to the affected tissue, annotations acquired through Cellossaurus split the CCLE cell lines into 21 different cancer types. In agreement with the original publications [37,39], expression data were obtained through RNA-sequencing and processed to obtain level expression in transcripts per million by the expectation-maximization algorithm (file: CCLE_RNAseq_rsem_genes_tpm_20180929.txt.gz). Copy Number Variation (CNV) data were acquired from the Affymetrix SNP6.0 Arrays (file: CCLE_copynumber_byGene_2013-12-03.txt.gz). Copy numbers were normalized by the most similar HapMap normal samples [40]. Segmentation of normalized log2 (CN/2) ratios was achieved using the circular binary segmentation algorithm [37,41]. Methylation data were derived by quantifying CpG islands using Reduced Representation Bisulfite Sequencing (file: CCLE_RRBS_tss_CpG_clusters_20181022.txt.gz). Global chromatin profiling was attained using multiple reaction monitoring for 42 combinations of histone marks (file: CCLE_GlobalChromatinProfiling_20181130.csv). Metabolomics data were acquired in parallel with global chromatin profiling by reporting the abundance measures of 225 metabolites (file: CCLE_metabolomics_20190502.csv). MicroRNA associated with cancer dependencies was correlated, regarding 734 microRNAs, with the Achilles gene dependency dataset. Protein profiling was measured with Reverse Phase Protein Arrays for 213 antibodies (file: CCLE_RPPA_20181003.csv) [39].

Dimensionality reduction of omics data. Data were normalized by removing the mean and scaling to unit variance with scikit-learn's StandardScaler [36]. Due to the omics data's high complexity, we performed dimensionality reduction to minimize the noise introduced in the dataset by highlighting the essential features. The datasets already described were used to build and train a Multi-Layer Perceptron (MLP) autoencoder, an unsupervised Artificial Neural Network (ANN) with a typical "hourglass" architecture, which is often used to perform dimensionality reduction in vast and high-dimensional datasets such as the ones observed with omics data [42–44]. This type of MLPs usually consists of three parts; an encoder that abstracts the input into hidden variables, i.e., a latent-space representation, a bottleneck layer that holds the smallest Hidden Layer (HL) (for purposes of dimensionality reduction, this is the layer that defines the size of the reduced dataset), and a decoder that reconstructs the original input data from the hidden data [45,46]. Seven autoencoders, one for each of the CCLE feature blocks, were developed by using Keras with a TensorFlow for Graphics Processing Units (GPU) (Version 2.3.1) backend [47]. Each of the autoencoders comprised seven layers, of which five were HLs. The input and output layers follow the number of available features in all cell lines, as displayed in **Table 2**. The number of nodes within the bottleneck layer of each of the seven autoencoders (used for extraction of the encoded features) corresponds to the autoencoder's final number of features. The two HL in each of the encoder and decoder sections vary in size according to the number of samples and features available (**Table 1_Supplementary Material**). In this stage, all models used Adam [48] as an optimizer function with a learning rate of 0.001. Rectified Linear Unit (ReLU) activation function was used in all layers. Mean Square Error (MSE) was used as a loss function. The models were trained for 1000, 250, or 100 epochs, depending on the dataset size (**Table 2_Supplementary Material**). After training, each autoencoder's bottleneck layer was used to perform dimensionality reduction of the omics data according to **Table 2**.

PCA, a commonly used method for dimensionality reduction [49], was also applied in the same datasets as the autoencoder, for which 25 Principal Components (PCs) were defined. It means that, by using PCA, each of the datasets was transformed to yield only 25 features, totalling 175 features to describe each unique cell line. As shown in **Table 2**, each feature block from CCLE had its variance explained in a range from 0.89 to 0.99. Since the seven blocks were used simultaneously for each sample, each cell line is thoroughly described by the components extracted with the PCA. Missing values (in both

autoencoder and PCA) were processed by either dropping the sample entirely or replacing the missing values by zero.

Table 2. Number of features pertaining the omics data and the corresponding amount for both the autoencoder and the PCA processing.

Omics Data	Number of available cell lines	Number of available features	Number of features after autoencoder	Number of features after PCA	Explained variance (PCA)
Expression	1019	57820	1156	25	0.89
Copy Number Variation	1043	23316	466		0.91
Methylation	843	56146	1122		0.92
Global Chromatin Profiling	897	42	21		0.99
Metabolomics	928	225	112		0.99
microRNA	954	734	73		0.95
Proteomics	899	214	107		0.93

Model evaluation and performance metrics

After data acquisition and pre-processing, we gather all datasets and to evaluate the results in the most unbiased manner possible, we randomly isolated three datasets considering different scenarios:

i) leave cell out dataset - 3 randomly chosen cell lines belonging to different tissue types (regression dataset: 13.810 combinations; classification dataset: 1.396 synergistic and 429 non-synergistic samples after processing the 13.810 combinations for full agreement) (for the tissue type classification see **Figure W1** of the SYNPRED webserver).

ii) leave drugs out dataset - 5 drugs with the majority belonging to different hierarchical clusters (regression dataset: 25.993 combinations; classification dataset: 2.934 synergistic and 622 non-

synergistic samples after processing the 25.993 combinations for full agreement) (for drugs hierarchical clustering see **Figure W8** of the SYNPRED webserver).

iii) leave drug combinations out dataset - 5 drug combinations (regression dataset: 360 combinations; classification dataset: 74 synergistic and 6 non-synergistic samples after processing the 360 combinations for full agreement).

After extracting the datasets for validation, we split the remaining data into training and test sets on an 80/20 ratio (**Table 3_Supplementary Material**). As such, the train dataset was composed of 195.996 combinations to be used for regression tasks that upon full agreement processing, yielded 20.291 synergistic and 6.419 non-synergistic samples for classification tasks. The test set was composed of 48.999 combinations to be used for regression tasks, which upon full agreement processing, yielded 5.084 synergistic and 1.553 non-synergistic samples for classification tasks. The described data splitting was performed prior to any model training, thus ensuring all the prediction models' performance evaluation is deployed on the same data. The binary classification models were evaluated through accuracy (acc), precision (prec), recall (rec), Area Under the Receiver Operating Characteristic (AUROC) as well as F1-score as previously described [50]. The regression models were evaluated through Root-Mean-Square Error (RMSE), Mean Squared Error (MSE), Mean Absolute Error (MAE) [51], Pearson and Spearman correlation coefficients [52].

Development of Machine Learning models

Neural Networks with Keras. The classification and regression neural networks were fully developed using Keras with a TensorFlow (Version 2.3.1) backend [47]. Weights were updated using Adam optimizer [48] and a learning rate of 0.0001 along 125 epochs with binary cross-entropy (classification) and MSE (regression) as the loss functions. All the HL were connected through ReLU activation, while the output layer was subject to sigmoid (classification) or linear activation (regression). As an initial approach, we performed a gridsearch for parameter optimization using 5% of the training set, fully detailed in the "Parameter optimization" section. The best performing parameters were further selected and used to train the models with the complete train dataset.

Machine Learning algorithms with scikit-learn. The datasets presented in this work were also trained with the most commonly used algorithms for synergy prediction tasks, namely with Random Forest (RF) [53], Extreme Randomized Trees (ETC) [50,54], Support Vector Machines (SVM) [55], Stochastic

Gradient Descent (SGD) [56], k-Nearest Neighbours (kNN) [57], and Extreme Gradient Boosting (XGBoost) [58]. The RF, ETC, SVM, SGD, and kNN models were built using the Python package "SciKit Learn" (Version 0.22.1) [36]. The XGBoost model was built using its dedicated package for Python (available at the Python Package Index as "xgboost") [58]. These six algorithms were also subject to gridsearch for parameter's optimization using 5% of the training set as described in the "Parameter optimization" section, with the best ones used to train the models with the full dataset.

Parameter optimization. To properly perform parameters' optimization in all the algorithms described, a grid search was performed using in-house scripts for Keras DL models and scikit-learn's GridSearchCV with 3-fold cross-validation (for ML algorithms with scikit-learn). We used 5% of the training set [59], a value in agreement with subset usage for parameter optimization [60], since using the full training dataset would exponentially increase an already long task. For each of the Keras classification and regression DL models, we performed gridsearch with 192 runs with parameters covering the four available dimensionality reduction datasets (PCA, PCA_drop, autoencoder, autoencoder_drop), 12 different network architectures, and four different dropout rates (0.00, 0.25, 0.50, 0.75) (**Table 4_Supplementary Material**). In the case of each of the six classification and regression ML models trained with scikit-learn, we used a total of 820 runs, including different parameters and dataset combinations (**Table 5_Supplementary Material**). Finally, for the six possible targets (full agreement, Bliss, HSA, Loewe, ZIP and CSS), we trained each of the six ML models with the best corresponding performing parameters. We then assessed the best performing architectures and dropout rates for the DL-based models. For each of the possible evaluation metrics we then trained the best performing parameters which can lead to different number of DL-based models depending on the synergy reference model used due to parameters overlap.

Ensemble algorithms. After selecting the previous best-performing models we replaced the outliers by the average of the remaining prediction values. For some tasks, a few of the individual predictors had notably bad performance (mostly SGD and KNN), as such we considered outliers the synergy prediction values above or below 10 times the average of the remaining prediction values; this was necessary to allow the ensemble neural networks to converge. These were used to constitute a new feature representation of the samples that could undergo ensemble model training. The ensemble models were first subjected to a new gridsearch for parameter optimization (**Table 6_Supplementary Material**),

taking the target probability of the selected algorithms as features, ultimately developing a neural network that worked as an ensemble method. This neural network had a learning rate of 0.0001, trained for three epochs, used the Adam optimizer [48] and binary cross-entropy and MSE for classification and regression, respectively, as the loss functions. All the HL were connected through ReLU activation, while the output layer was subject to sigmoid or linear activation for classification and regression, respectively. The best-performing ensemble models were trained with the prediction-based feature space.

Feature Contribution

To understand what the top contributors were to the most for accurate predictions, we assessed their predictive power. For that, we needed first to break down the process of assessing feature contribution into two stages due to the dimensionality reduction of cell lines. First, since the best performing dimensionality reduction approach was the PCA, we considered the explained variance by each of the features concerning the respective Principal Component (PC). This information was then extracted as an attribute from the PCA object using scikit-learn [36]. Secondly, we used the eli5 package [61], with Python deployment, to assess final feature weight by deploying Permutation Importance [53], a method that allows iterative exclusion of each of the features, to assess its contribution to the predictive model. The Permutation Importance was deployed on the test set because it would not be possible to assess the feature contribution under unbiased conditions if the training set had been used. However, it is worth noting that this evaluation occurs after all model training; hence, it does not influence the test results.

Benchmark

Benchmarking of synergy prediction protocols is a very complicated process. As reviewed by Zagidullin et al. [29], the datasets available completely differ in the amount of information used, with DrugComb [29] assembling the most important ones (ALMANAC [30], ONEIL [62], FORCINA [63], CLOUD [64]). As showed by Kumar et al. [65], majority of authors used NCI ALMANAC to train and the concept of Loewe additivity model [14,16–18,20,21]. Furthermore, comparison to the available methodologies implies that authors adapt the published proposed DL architectures as these are not easily applied or not available in GitHub or similar platforms (e.g., pruning the data due to unavailability of a certain data modality, or changing the loss function to turn a model into a regressor).

As such, we followed a multi-step approach to benchmark our pipeline:

i) Comparison of DL architectures and simpler ML algorithms (RF, ETC, SVM, SGD, kNN and XGBoost models) with ensemble approaches in 4 different test scenarios.

ii) DeepSynergy [18] architecture implementation and comparison using our independent test set and validation sets as this is one of the most common approaches. As described in the original paper, we retrained a model using two hidden layers, the first with 8192 and the second with 4096 neurons. Furthermore, two dropout layers were added, the first with 0.2 rate and the second with 0.5 rate. The activation function used between the hidden layers was hyperbolic tangent and on the output layer linear activation was used. This was trained over 250 epochs with a learning rate of 0.00001 and an Adam optimizer.

iii) Comparison with published methods for synergy calculations using both regression (12 models) and classification (13 models) approaches as reviewed by Kumar et al. [65].

iv) Comparison of our regression approaches to algorithms for which the training dataset was clearly available to make sure the comparison would be as fair as possible. As such, we compared to the Matchmakers' algorithm [20], using the adapted DrugCombo (retrieved from Matchmakers' [20] Github) and NCI ALMANAC complete datasets, which, in turn, enables us also to compare with DeepSynergy [18] and TreeCombo [12] as these were also evaluated by the authors [20]. Upon the data considered, we performed our own feature extraction, as described in the SynPred pipeline. Thus, the comparison is now possible between the full methods, of which the feature extraction is a part, enabling us to the comparison with the values reported by the authors.

Web-based application interface implementation

The SYNPRED prediction models were implemented in a web-based application at http://www.moreiralab.com/resources/synpred/. The website's plots and front-end were constructed with plotly [66] and Flask [67], both freely available Python packages, on a framework that uses an in-house adaptation of Javascript, CSS, and HTML scripts. All the back-end hosting was mediated with Flask [67].

RESULTS AND DISCUSSION

Measuring feature importance for model development

To understand the importance of each group of included features for the final model performance and to attain a more interpretable model, we analysed each of the individual models with Permutation Importance. We perceived that more complex models, particularly DL-based models with different architectures, tend to make more extensive use of the omics-based features to over 70% of the total feature contribution (**Figures W9-W12** of the SYNPRED webserver). Contrarily, simpler models, such as kNN and SGD, made almost exclusive use of the drug features (above 90%) (**Figures W16** and **W18** of the SYNPRED webserver). Other non-DL based models made variable (between 20%-80%) usage of the omics features (**Figures W13-W15** and **W17** of the SYNPRED webserver). This observation highlights the importance of DL models to take full advantage of omics data for capturing the complexity of each cancer profile, thus improving drug pair-cell line combinations predictions. The advantages of using these algorithms when dealing with multi-dimensional omics data, particularly the great flexibility of DL architectures, was also previously emphasised [68].

We then looked for a possible biological relevance of the Top 5 genes in each group of the most critical multiomics features to understand if genes contributing more for the prediction models were also implicated in tumorigenesis. Of the 15 ranked genes from expression, methylation and CNV variations, all of them, are used as prognostic cancer markers, or have a role in tumour progression and treatment (**Table 3**). These data suggest that our models, especially DNNs, are likely to capture the most relevant information for each group of multiomics features for synergistic drug combinations. The remaining ranked genes organised by each ML model's best-contributing features are presented in interactive Sankey diagrams on the website landing page (**Figures W9-W18**).

Table 3. Permutation importance of the Top 5 proteins associated with expression, methylation and CNV features as well as their associated biological relevance.

Type of Feature	Gene Name	Protein Description	Biological relevance [a]
**Expression**	TMSB4X	Thymosin beta-4 X-linked	Prognostic marker in renal cancer (unfavourable)
	MTCO2	Mitochondrially encoded cytochrome c oxidase II	Prognostic marker in liver cancer (favorable) and pancreatic cancer (favorable)
	MT-RNR2	Mitochondrially encoded 16S RRNA	Associated with survival outcomes in cancer patients [69]
	MT-CO3	Mitochondrially encoded cytochrome c oxidase III	Prognostic marker in pancreatic cancer (favorable) and liver cancer (favorable)
	COX6C	Cytochrome C oxidase subunit 6C	Associated with breast cancer, thyroid tumors, uterine cancer, prostate cancer and esophageal cancer [70] although not reported as prognostic
**Methylation**	C11ORF52	Chromosome 11 open reading frame 52	Associated with lung cancer [71] although not reported as prognostic
	NPY1R	Neuropeptide Y receptor Y1	Prognostic marker in breast cancer (favourable)
	TMBIM6	Transmembrane BAX inhibitor motif containing 6	Prognostic marker in renal cancer (favorable), head and neck cancer (unfavorable) and breast cancer (unfavorable)
	C2CD4D	C2 calcium dependent domain containing 4D	C2CD4D-AS1 overexpression contributes to the malignant phenotype

Type of Feature	Gene Name	Protein Description	Biological relevance [a]
			of lung adenocarcinoma cells [72] although not reported as prognostic
	EDNRB	Endothelin receptor type B	Prognostic marker in renal cancer (favorable)
**CNV**	UTY	Ubiquitously transcribed tetratricopeptide repeat containing, Y-linked	Associated with cutaneous melanoma, bladder urothelial carcinoma, B cell lymphoma, small cell lung cancer, oligodendroglioma, chondroblastic osteosarcoma, and cutaneous melanoma [73,74], although not reported as prognostic
	MACROD2	Mono-ADP Ribosylhydrolase 2	Associated with growth of intestinal tumours [75], although not reported as prognostic
	WWOX	WW domain containing oxidoreductase	Prognostic marker in renal cancer (favorable) and breast cancer (unfavorable)
	DAZ2	Deleted in azoospermia 2	Associated with oligozoospermia [76] which is, in turn, highly associated with testicular cancer [77], although not reported as prognostic
	KANK1	KN motif and ankyrin repeat domains 1	Upregulating Kank1 gene inhibits human gastric and lung cancers progress [78,79], although not reported as prognostic

[a] The protein description and biological importance were retrieved from The Human Proteins Atlas (https://www.proteinatlas.org/) and The Human Gene Database (https://www.genecards.org/). When this

information was not listed in these databases, we presented the study that supports the biological relevance. Favourable and unfavourable is related to gene contribution for cancer progression.

Tuning and choosing the best Machine Learning parameters

ML performance and training time are deeply affected by specific model parameters, so an appropriate choice of the best ones should always be performed. With that in mind, we used a gridsearch approach to test a comprehensive array of parameters and dataset combinations, including parameters for several ML methods, a comprehensive set of DL configurations and pre-processing setups, as described above. Regarding the pre-processing datasets, autoencoder datasets performed worse in the training sets and slightly worse for the test set, which led us to discard it as there was no benefit to the increased training time caused by the significantly higher dimensionality. We proceed with the dataset in which PCA was used for dimensionality reduction and replacing the missing values with 0, as these approaches performed better for most gridsearch runs [80,81] (**Table 3_Supplementary Material**).

SYNPRED models for drug combination prediction

After selecting the best parameters for both DL with Keras and ML with scikit-learn, we trained models with the full training set according to the parameters in the best gridsearch performing metrics. The best individual models were used to attain each sample prediction to make the final ensemble for the four-synergy reference model plus the full-agreement. The final models were then evaluated in the test set and three different scenarios: leave cell out, leave drugs out and leave drug combinations out**,** by attaining different classification (**Table 7_Supplementary Material**) or regression (**Table 8-12_Supplementary Material**) evaluation metrics.

Classification model performance. Prior to ensemble development, the best independent performing model was XGBoost with the following parameters: alpha = 0.25, max_depth = 6, n_estimators = 100. After ensemble, our final full-agreement SYNPRED comprised four DL-based and six ML-based models, attained with a DL architecture with three hidden layers of size one hundred and a dropout rate of 0.60. When applied in an independent test set, our ensemble model displayed better performance (accuracy = 0.85, precision = 0.91, recall = 0.90, AUROC = 0.80, and F1-score = 0.90) than any other classic ML or DL models, including reference ones such as SVM, RF or XGBoost frequently used for

synergy prediction classification tasks (**Table 4, Table 7_Supplementary Material**) [13,14,82]. In the three independent scenarios, the full-agreement ensemble SYNPRED achieved higher precision values by returning the most relevant results than any other of the individual models. However, it saw a significant drop in the leave cells, drugs, and drug combinations out datasets.

Table 4. Best results obtained for the classification ensemble model.

Subset used for evaluation [a]	Accuracy	Precision	Recall	AUROC	F1-Score
**Test**	0.85	0.91	0.90	0.80	0.90
**Leave cells out**	0.37	0.89	0.13	0.55	0.22
**Leave drugs out**	0.33	0.86	0.13	0.53	0.22
**Leave drug combinations out**	0.24	1.00	0.21	0.61	0.35

[a] The final model had a dropout rate of [0.4] and an architecture of [10, 10, 10].

Regression model performance. Concerning the five regression tasks (**Table 5**), CSS (**Table 12_Supplementary Material**) clearly stands out - in either the metrics or the datasets considered - while the remaining four: ZIP, HSA, Bliss and Loewe (**Table 8-11_Supplementary Material**) followed closely behind. Although in agreement with the presented data, this is unexpected considering the literature on the subject, which mainly uses Loewe. Indeed, historically, Loewe has been systematically chosen as the target regression reference model [14,16–18,20,21]. For most cases in which this happens, there is no comparison with the remaining reference models. The few available comparative studies are mainly done outside of the spectrum of synergy prediction and somewhat under the scope of analysing provided drug combination dose-response matrix data [33,83]. By deploying unbiased data-driven selection of the model, SYNPRED empirically assesses how realistically viable is the representation of five of the most common synergy reference models against a real biological dataset. Zagidullin et al. has already point to the value of such agglomerative approaches [29].

The results of our best final ensemble regression model (CSS) outperformed all the individual predictors when evaluated in the test dataset and leave drug combinations out scenario, one of the most challenging ones (**Table 5**). Regarding correlation metrics, and comparing to the literature standards [84], CSS achieved strong Pearson values (0.86 on test and 0.74 on the leave cells out dataset). The CSS had 11.07 and 13.63 RMSE on the test and leave cells out datasets, respectively, regarding scale-

depending performance metrics. Considering that CSS values range within [-54.05,99.84], our predictor was able to determine CSS synergy values with a low error (**Figure 3**). A similar pattern was exhibited by the Loewe ensemble predictor (**Figure 4**).

Table 5. Best results obtained for the regression ensemble models, considering the test dataset.

Synergy reference model	RMSE	MSE	Pearson	MAE	Spearman
**CSS**	11.07	122.61	0.86	7.43	0.87
**Loewe**	10.58	111.92	0.71	6.49	0.68
**Bliss**	4.35	18.92	0.71	3.07	0.59
**HSA**	4.09	16.70	0.73	2.86	0.64
**ZIP**	3.86	14.87	0.70	2.74	0.66

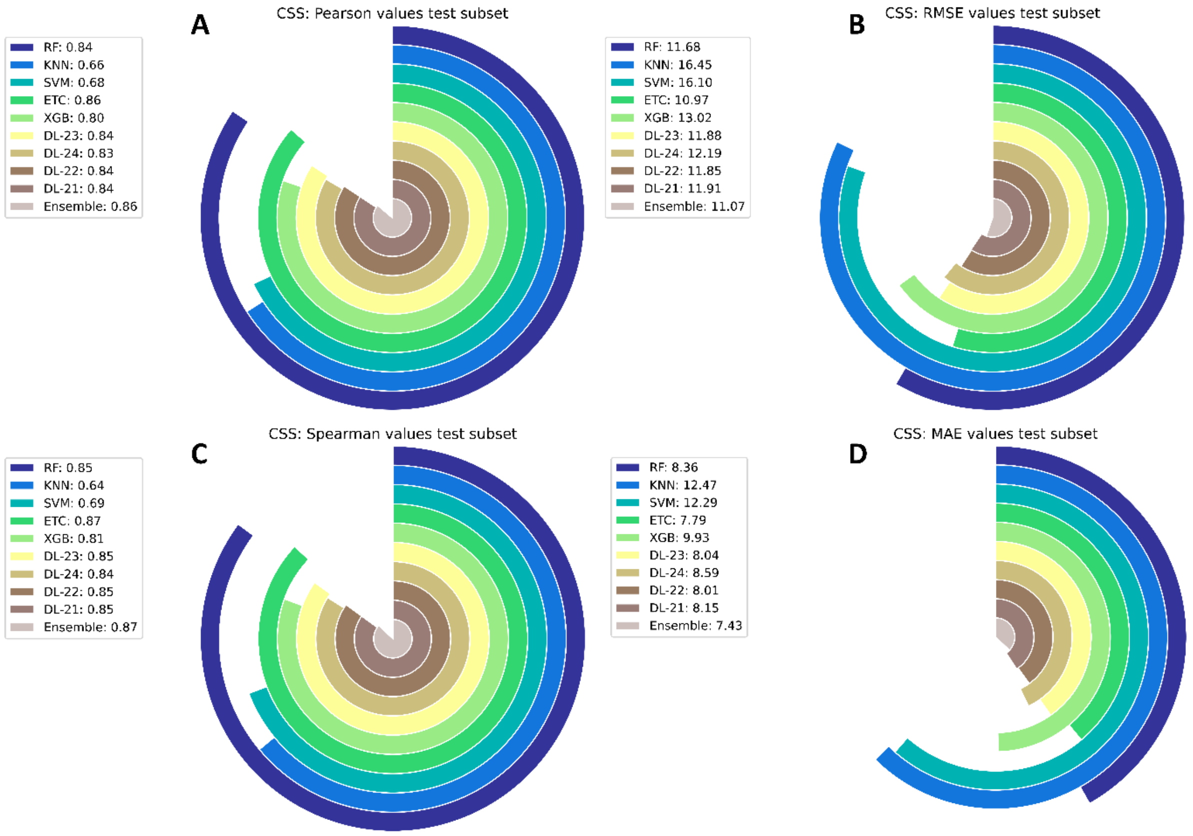

Figure 3. Circular bar plot representing the model's evaluation metrics for the CSS synergy reference model. (A) Models performance Pearson values evaluated in the test dataset, (B) Models performance RMSE values evaluated in the test dataset, (C) Models performance Spearman values evaluated in the test dataset, (D) Models performance MAE values evaluated in test dataset.

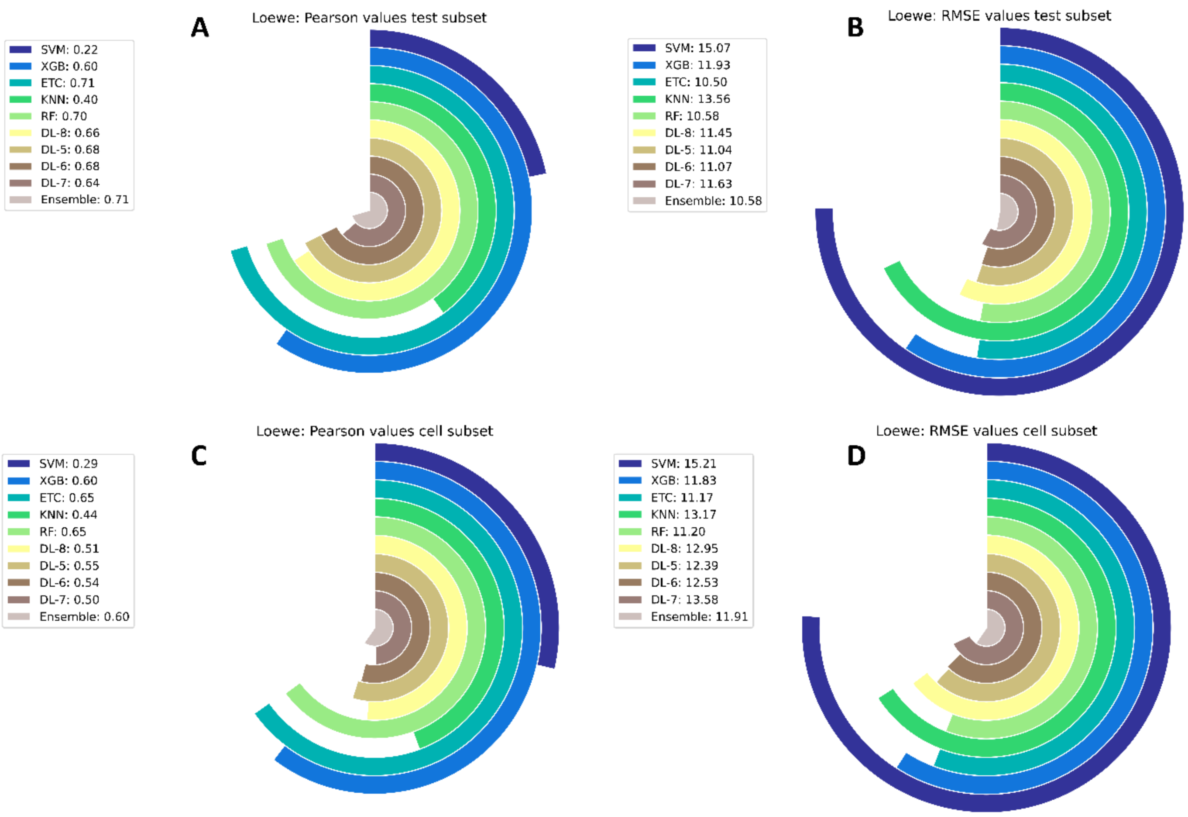

Figure 4. Circular bar plot representing the model's evaluation metrics for the Loewe synergy reference model. (A) Models performance Pearson values evaluated in the test dataset, (B) Models performance RMSE values evaluated in the test dataset, (C) Models performance Pearson values evaluated in the leave cells out dataset, (D) Models performance RMSE values evaluated in the leave cells out dataset.

Benchmark

We benchmarked our pipeline following a multi-step approach as described in the methods section:

i) Comparison of the best performing individual DL and ML algorithms with the ensemble approaches for each prediction task - Tables 7 to 12_Supplementary Material.

ii) DeepSynergy [18] architecture implementation and comparison using our independent test set and validation sets - **Table 13_Supplementary Material.**

iii) Comparison with published methods for synergy calculations as reviewed by Kumar et al. [65] - **Table 14_Supplementary Material**.

iv) Comparison of our regression approaches to Matchmakers' algorithm [20], DeepSynergy [18] and TreeCombo [12] - **Tables 15** and **16_Supplementary Material**.

Regarding i), ensemble/aggregation of algorithms consistently outperform, or stand very close to the best individual predictors. XGBoost and Extreme Randomized Trees were typically the second-best predictors. This showcases how SynPred leverages previous information on algorithms such as TreeCombo [12] (uses an individual XGBoost algorithm) or DeepSynergy [18], which is, in essence, the literature parent of several of the neural networks with conic architecture we used. In fact, in ii), (**Table 13_Supplementary Material**) it can be seen that the DeepSynergy [18] implementation on SynPred's pipeline behaves similarly to other DNN approaches in SynPred. These are good performers, but unable to beat the ensemble algorithms.

When comparing the reported performance for algorithms in their own settings iii), as reviewed by Kumar et al. **[65]**, once again we need to take into account a very broad array of circumstances, such as algorithms, datasets (filtered or postprocessed), and synergy reference models (**Table 14_Supplementary Material**). The high possible combination of factors that leads to the final methods' performance are huge, and therefore this comparison has to be conducted with a limited few.

For instance, SynPred highest performer predictor is clearly the CSS predictor. However, it is not possible to justly compare our result to predictors that only focus on the Loewe synergy reference model. On the other hand, when considering the most recurring synergy reference model (Loewe), albeit SynPred shows lower Pearson and Spearman correlations, it also shows much lower errors (RMSE and MSE) in comparison to the best remaining algorithms. All this serves to highlight the need to account for different synergy reference models, which had not been previously achieved, but already suggested to be a valuable approach **[29]**.

Finally (iv), we performed closer comparisons (although still not optimal) in **Table 15_Supplementary Material** and **Table 16_Supplementary Material**. These more recent applications use more readily available datasets. Regarding **Table 15_Supplementary Material**, SynPred was ran against Matchmakers's' [20] processing of DrugComb [29]. Upon doing this, both CSS and Loewe predictors

from SynPred stood very close to the performance of Matchmakers' [20], which is remarkable, since this was the dataset used by the authors [20] to train the dataset. When inspecting **Table 16_Supplementary Material**, in which the predictors were deployed upon NCI ALMANAC [85], the dataset in which SynPred focuses, SynPred clearly stands out in all the synergy reference models with Pearson and Spearman correlations performance increasements between 30.51% and 42.37%, and between 36.36% and 56.36%, respectively. Although MSE metrics are particularly hard to compare between datasets and methods, significant improvements were also observed.

Web-based application description

The classification and regression models for predicting the type of combinatory effect in dug pair-cell line samples is available as a web-based application at http://www.moreiralab.com/resources/synpred/. All the 11 described single models are deployed on user submission, as well as the ensemble approach. The user needs to submit two drugs as input in the *.smile format and selects from a dropdown menu, the primary body site corresponding to the tested cancer cell lines. The drugs are then subject to feature extraction by Mordred and a standard pre-processing (feature elimination and normalization) as thoroughly described in the methods section. The output, displayed in a downloadable heatmap, is the drug combination prediction effect for each of the individual cell lines calculated with the ensemble classification and regression models and using five synergy reference models (ZIP, HSA, Bliss, Loewe, CSS) plus the full-agreement metric. Furthermore, it is also displayed in the last column ("Synergy Votes") the final tally of synergistic queries predicted by all models based on the prediction values. This facilitates the visualization of the type of combinatory effect between the two drugs and aims at strengthening the value of the prediction due to the lack of consensus between the difference synergy reference models. The results are returned to the provided email and displayed on the submission web page. Additionally, users can assess, explore, and visualize through different plots as well as export a summary of the synergy scores (calculated using ZIP, Bliss, HSA, Loewe, and CSS synergy reference models) by cell line used to develop the original dataset of SYNPRED. To our knowledge, this is the first webserver that can predict new drug synergy combinations without the need of uploading a partial or full dose-response matrix. This feature is an advantage compared with other models implemented in webservers that need these types of data for drug combination response prediction [28,29]. The output is displayed, upon submission, as shown in Figure 5.

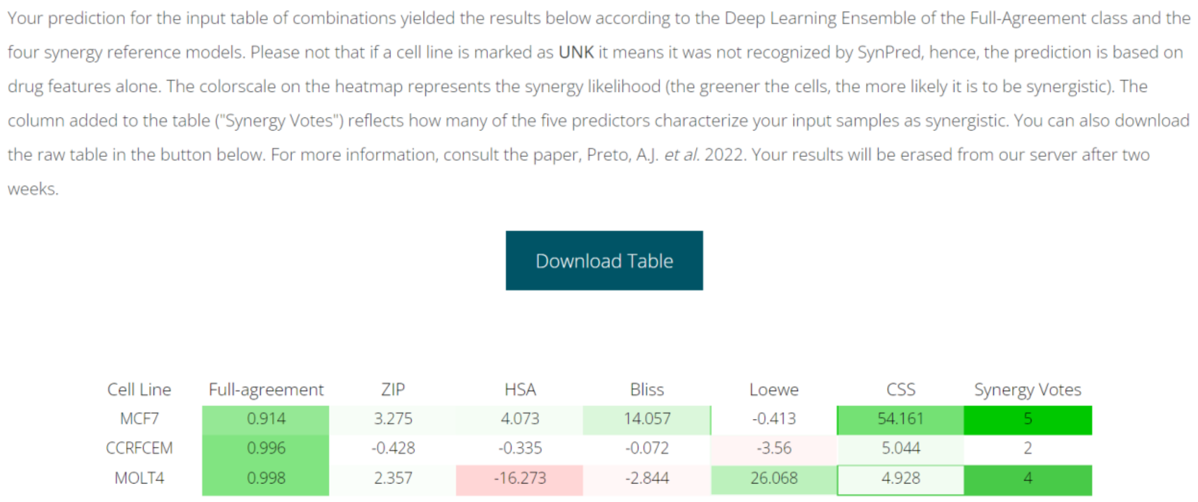

Your prediction for the input table of combinations yielded the results below according to the Deep Learning Ensemble of the Full-Agreement class and the four synergy reference models. Please not that if a cell line is marked as UNK it means it was not recognized by SynPred, hence, the prediction is based on drug features alone. The colorscale on the heatmap represents the synergy likelihood (the greener the cells, the more likely it is to be synergistic). The column added to the table ("Synergy Votes") reflects how many of the five predictors characterize your input samples as synergistic. You can also download the raw table in the button below. For more information, consult the paper, Preto, A.J. *et al.* 2022. Your results will be erased from our server after two weeks.

Download Table

Cell Line	Full-agreement	ZIP	HSA	Bliss	Loewe	CSS	Synergy Votes
MCF7	0.914	3.275	4.073	14.057	-0.413	54.161	5
CCRFCEM	0.996	-0.428	-0.335	-0.072	-3.56	5.044	2
MOLT4	0.998	2.357	-16.273	-2.844	26.068	4.928	4

Figure 5. Example of the SYNPRED output prediction, green coloured cells represent synergistic prediction, while red coloured cells represent non-synergistic samples.

CONCLUSIONS

Synergistic anticancer drug combinations are a powerful tool to help tackle cancer drug resistance since they can simultaneously target multiple key molecules or pathways. The rational design of combination therapies is warranted to improve the efficacy, although this is a well-known time-consuming and expensive task. In recent years, ML algorithms' applicability for drug-repurposing or novel drug design has been essential to demonstrate the importance of *in silico* methodologies to help overcome this problem. Some classification [13,14,17] and regression [16,18–22] models using ML and omics data for predicting drug synergy combinations were already developed. However, the fittingness of the previously developed algorithms is sometimes hindered by using a single reference model (e.g., Bliss, Loewe, HSA, ZIP or CSS) or by the difficulty in applying these models on new unseen data, since these are not straightforward to implement and require advanced bioinformatics skills. Additionally, our study leads to an innovative approach by highlighting the importance of choosing an appropriated synergy reference model and explores this choice influence in the final predictor performance. Given the different sensitivity observed between these reference models in evaluating the degree of combination, a more comprehensive and rigorous approach that leverages all metrics to predict drug synergy is an asset.

This study introduced a new synergy prediction model, SYNPRED, that combines comprehensive multiomics data of cancer cell lines with physicochemical and structural features of drugs. This work is one of the first that takes five different synergy reference models (Bliss, HSA, Loewe, ZIP and CSS) and uses one of the most comprehensive and balanced databases regarding the synergistic-non synergistic distribution, the NCI ALMANAC. Our top-ranked classification and regression models, an ensemble developed with the best machine learning models, achieved state-of-the-art performance to predict synergistic drug combinations in an independent dataset. The best performing prediction model in SYNPRED is, undoubtedly, CSS (RMSE – 11.07, MSE – 122.61, Pearson – 0.86, MAE – 7.43, Spearman – 0.87). However, we advise the users to take into consideration the aggregate of results, albeit with a higher focus on CS. To aid the user interpretation of the results, there is also a "Voting classifier" output, that tallies the results of the 6 predictors; if more than 5 predictors yield a positive result, it is likely that the submission sample is synergistic, while if it is only one or lower, it is likely to be non-synergistic. Besides, we provide the complete workflow for a standalone deployment in our GitHub coupled with a freely available and easy-to-use webserver (http://www.moreiralab.com/resources/synpred/) that only requires two drugs' SMILES as inputs, thus alleviating the need of uploading a conventional and laborious dose-response matrix. SYNPRED can be a valuable tool to the scientific and medical community for drug repurposing or *in-silico* discovery of new anticancer drug combinations.

Additionally, given the importance of multiomics data in cell line classification and therapy response, we combined all the available multiomics features in the CCLE database to explore their individual contribution to model development. The knowledge mined from this analysis demonstrates the capacity of different ML models to deal with multiomics data, with DL algorithms being much more able to learn and leverage this complex type of features. We found that the most ranked proteins in each of the most contributing multiomics features are important cancer biomarkers or have a role in tumorigenesis, demonstrating DNN models' capacity to capture their significance and use this information for the final model development. In the future, we expect to include protein-protein interactions data and network analysis to improve the model performance, aiming to identify drug combinations with potential new targets across different cell lines.

AVAILABILITY OF SOURCE CODE AND REQUIREMENTS

Project name: SYNPRED

Project home page: https://github.com/MoreiraLAB/synpred

Operating system(s): Linux, Mac OS X, Windows

Programming language: Python and R

Other requirements: Python 3.8.2 or higher, R 3.6.3 or higher

License: GPL-3.0

Biotools: Synpred

RRID: [EDITOR's NOTE: RRID to be confirmed – PRODUCTION: Please ask authors to add the RRID nr here]

DATA AVAILABILITY

SYNPRED is a free, open-source web-based application available at http://www.moreiralab.com/resources/synpred/ without any login or registration requirements. The source code of the web-based application implementation is deposited in the GitHub repository (https://github.com/MoreiraLAB/synpred) to allow the stand-alone use of the application and further integration and comparison with other models. The code is fully developed in Python and R languages; hence, it can be deployed fully without charge. The multiomics data included in this study are available at the corresponding references mentioned in the main text. Supporting data and an archival copy of the code are also available via the GigaScience database GigaDB [89].

ADDITIONAL FILES

Supplementary Table 1. Conditions for dimensionality reduction with autoencoders. Hidden and bottleneck layers definition according to the Number of Features.

Supplementary Table 2. Conditions for dimensionality reduction with autoencoders. Number of epochs of the autoencoder training according to either the Number of Samples or Number of Features.

Supplementary Table 3. Final datasets to be subjected to training.

Supplementary Table 4. Gridsearch combination parameters using 5% on the training set with Deep Learning algorithms.

Supplementary Table 5. Gridsearch combination parameters using 5% on the training set with non-Deep Learning algorithms.

Supplementary Table 6. Gridsearch combination parameters of the ensemble neural network.

Supplementary Table 7. Final metrics of the classification models evaluated in an independent test set and three different scenarios (leave cell out, leave drugs out and leave drug combinations out) using Full-Agreement synergy values.

Supplementary Table 8. Final metrics of the regression models evaluated in an independent test set and three different scenarios (leave cell out, leave drugs out and leave drug combinations out) using Bliss synergy reference model.

Supplementary Table 9. Final metrics of the regression models evaluated in an independent test set and three different scenarios (leave cell out, leave drugs out and leave drug combinations out) using HSA synergy reference model.

Supplementary Table 10. Final metrics of the regression models evaluated in an independent test set and three different scenarios (leave cell out, leave drugs out and leave drug combinations out) using Loewe synergy reference model.

Supplementary Table 11. Final metrics of the regression models evaluated in an independent test set and three different scenarios (leave cell out, leave drugs out and leave drug combinations out) using ZIP synergy reference model.

Supplementary Table 12. Final metrics of the regression models evaluated in an independent test set and three different scenarios (leave cell out, leave drugs out and leave drug combinations out) using CSS synergy reference model.

Supplementary Table 13. DeepSynergy [86] reimplementation on the dataset that yielded the best results for SynPred (with PCA preprocessing and missing values replacement with 0), against the synergy reference model the original work targeted - Loewe.

Supplementary Table 14. Comparison of final metrics of the classification and regression models of SynPred to the methods reviewed by Kumar et al [65].

Supplementary Table 15. Comparison of the performance of SynPred and other recent algorithms, according to their respective reporting metrics upon deployment in DrugCombo [87].

Supplementary Table 16. Comparison of the performance of SynPred and other recent algorithms, according to their respective reporting metrics upon deployment in NCI ALMANAC [88].

ABBREVIATIONS

AI: Artificial Intelligence; ACC: Accuracy; ANN: Artificial Neural Network; AUROC: Area Under the Receiver Operator Curve; CCLE: Cancer Cell Line Encyclopaedia; CNV: Copy Number Variation; DL: Deep Learning; DNN: Deep Neural Networks; ENS: Ensemble; ETC: Extreme Randomized Trees; F1: F1-score; GPU: Graphics Processing Unit; HL: Hidden Layers; HSA: Highest Single Agent; kNN: k-Nearest Neighbours; MAE: Mean Absolute Error; miRNA: micro-RNA; ML: Machine Learning; MLP: Multi-Layer Perceptron; MSE: Mean-Square Error; PC: Principal Component; PCA: Principal Component Analysis; PREC: Precision; REC: Recall; ReLU: Rectified Linear Unit; RF: Random Forest; RMSE: Root-Mean-Square Deviation; SGD: Stochastic Gradient Descent; SMILE: Simplified Molecular-Input Line-Entry System; SVM: Support Vector Machine; SYNPRED: SYNergy PREDiction; XGBoost: Extreme Gradient Boosting; ZIP: Zero Interaction Potency

COMPETING INTERESTS

The authors declare that they have no competing interests.

FUNDING

This work was supported by the European Regional Development Fund through the COMPETE 2020 - Operational Programme for Competitiveness and Internationalisation and Portuguese national funds via Fundação para a Ciência e a Tecnologia (FCT) [LA/P/0058/2020, UIDB/04539/2020, UIDP/04539/2020, POCI-01-0145-FEDER-031356, and DSAIPA/DS/0118/2020]. FCT also supported

A.J.P. with a PhD scholarship [SFRH/BD/144966/2019]. Funding for open access charge: Fundação para a Ciência e a Tecnologia [DSAIPA/DS/0118/2020].

AUTHOR CONTRIBUTIONS

António J. Preto, Methodology; Software; Validation; Formal analysis; Investigation; Resources; Writing – review & editing; Visualization. Pedro Matos-Filipe, Methodology; Software; Investigation; Resources; Data curation; Writing – original draft preparation. Joana Mourão, Conceptualization; Methodology; Formal analysis; Data curation; Writing – original draft preparation; Writing – review & editing; Supervision; Project administration. Irina S. Moreira, Conceptualization; Writing – review & editing; Visualization; Supervision; Project administration; Funding acquisition.

ACKNOWLEDGEMENTS

Authors would like to acknowledge STRATAGEM - New diagnostic and therapeutic tools against multidrug-resistant tumors, CA17104.

REFERENCES

1. IARC IA for R on C. GLOBOCAN - Cancer Tomorrow via Global Cancer Observatory. 2020.

2. Vasan N, Baselga J, Hyman DM. A view on drug resistance in cancer. *Nature*. 2019; doi: 10.1038/s41586-019-1730-1.

3. Chatterjee N, Bivona TG. Polytherapy and Targeted Cancer Drug Resistance. *Trends in Cancer*. 2019; doi: 10.1016/j.trecan.2019.02.003.

4. Piochi LF, Gaspar AT, Rosário-Ferreira N, Preto AJ, Moreira IS. From single-omics to interactomics: How can ligand-induced perturbations modulate single-cell phenotypes? *Advances in Protein Chemistry and Structural Biology*. Academic Press;

5. Roell KR, Reif DM, Motsinger-Reif AA. An Introduction to Terminology and Methodology of Chemical Synergy—Perspectives from Across Disciplines. *Front Pharmacol*. 2017; doi: 10.3389/fphar.2017.00158.

6. Brandão M, Pondé NF, Poggio F, Kotecki N, Salis M, Lambertini M, et al.. Combination therapies for the treatment of HER2-positive breast cancer: current and future prospects. *Expert Review of Anticancer Therapy*. 2018; doi: 10.1080/14737140.2018.1477596.

7. Westerweel PE, te Boekhorst PAW, Levin M-D, Cornelissen JJ. New Approaches and Treatment Combinations for the Management of Chronic Myeloid Leukemia. *Front Oncol*. 2019; doi: 10.3389/fonc.2019.00665.

8. Xu J, Qiu Y. Current opinion and mechanistic interpretation of combination therapy for castration-resistant prostate cancer. *Asian J Androl*. 2019; doi: 10.4103/aja.aja_10_19.

9. Ribas A, Lawrence D, Atkinson V, Agarwal S, Miller WH, Carlino MS, et al.. Combined BRAF and MEK inhibition with PD-1 blockade immunotherapy in BRAF-mutant melanoma. *Nat Med*. 2019; doi: 10.1038/s41591-019-0476-5.

10. Wang Z, Deisboeck TS. Dynamic Targeting in Cancer Treatment. *Front Physiol*. 2019; doi: 10.3389/fphys.2019.00096.

11. Wang Z, Li H, Guan Y. Machine Learning for Cancer Drug Combination. *Clin Pharmacol Ther*. 2020; doi: 10.1002/cpt.1773.

12. Janizek JD, Celik S, Lee S-I. Explainable machine learning prediction of synergistic drug combinations for precision cancer medicine. Cancer Biology; 2018 May.

13. Li H, Li T, Quang D, Guan Y. Network Propagation Predicts Drug Synergy in Cancers. *Cancer Res*. 2018; doi: 10.1158/0008-5472.CAN-18-0740.

14. Celebi R, Bear Don't Walk O, Movva R, Alpsoy S, Dumontier M. In-silico Prediction of Synergistic Anti-Cancer Drug Combinations Using Multi-omics Data. *Sci Rep*. 2019; doi: 10.1038/s41598-019-45236-6.

15. Malyutina A, Majumder MM, Wang W, Pessia A, Heckman CA, Tang J. Drug combination sensitivity scoring facilitates the discovery of synergistic and efficacious drug combinations in cancer. Gallo J, editor. *PLoS Comput Biol*. 2019; doi: 10.1371/journal.pcbi.1006752.

16. Zhang T, Zhang L, Payne PRO, Li F. Synergistic Drug Combination Prediction by Integrating Multiomics Data in Deep Learning Models. In: Markowitz J, editor. *Translational Bioinformatics for Therapeutic Development*. New York, NY: Springer US;

17. Wang J, Liu X, Shen S, Deng L, Liu H. DeepDDS: deep graph neural network with attention mechanism to predict synergistic drug combinations. *Briefings in Bioinformatics*. 2021; doi: 10.1093/bib/bbab390.

18. Preuer K, Lewis RPI, Hochreiter S, Bender A, Bulusu KC, Klambauer G. DeepSynergy: Predicting anti-cancer drug synergy with Deep Learning. *Bioinformatics*. 2018; doi: 10.1093/bioinformatics/btx806.

19. Zhang H, Feng J, Zeng A, Payne P, Li F. Predicting Tumor Cell Response to Synergistic Drug Combinations Using a Novel Simplified Deep Learning Model. Bioinformatics; 2020 Apr.

20. Kuru HI, Tastan O, Cicek AE. Matchmakers': A Deep Learning Framework for Drug Synergy Prediction. *IEEE/ACM Trans Comput Biol Bioinform*. 2021; doi: 10.1109/TCBB.2021.3086702.

21. Liu Q, Xie L. TranSynergy: Mechanism-driven interpretable deep neural network for the synergistic prediction and pathway deconvolution of drug combinations. Schlessinger A, editor. *PLoS Comput Biol*. 2021; doi: 10.1371/journal.pcbi.1008653.

22. Xia F, Shukla M, Brettin T, Garcia-Cardona C, Cohn J, Allen JE, et al.. Predicting tumor cell line response to drug pairs with deep learning. *BMC Bioinformatics*. 2018; doi: 10.1186/s12859-018-2509-3.

23. Bliss CI. The toxicity of poisons applied jointly. *Annals of Applied Biology*. 1939; doi: 10.1111/j.1744-7348.1939.tb06990.x.

24. Foucquier J, Guedj M. Analysis of drug combinations: current methodological landscape. *Pharmacology research & perspectives*. 2015; doi: 10.1002/prp2.149.

25. Loewe S, Muischnek H. Über Kombinationswirkungen. *Archiv für Experimentelle Pathologie und Pharmakologie*. 1926; doi: 10.1007/BF01952257.

26. Chou T-C. Drug Combination Studies and Their Synergy Quantification Using the Chou-Talalay Method. *Cancer Research*. 2010; doi: 10.1158/0008-5472.CAN-09-1947.

27. Yadav B, Wennerberg K, Aittokallio T, Tang J. Searching for Drug Synergy in Complex Dose-Response Landscapes Using an Interaction Potency Model. *Computational and structural biotechnology journal*. 2015; doi: 10.1016/j.csbj.2015.09.001.

28. Ianevski A, Giri AK, Gautam P, Kononov A, Potdar S, Saarela J, et al.. Prediction of drug combination effects with a minimal set of experiments. *Nat Mach Intell*. 2019; doi: 10.1038/s42256-019-0122-4.

29. Zagidullin B, Aldahdooh J, Zheng S, Wang W, Wang Y, Saad J, et al.. DrugComb: an integrative cancer drug combination data portal. *Nucleic Acids Research*. 2019; doi: 10.1093/nar/gkz337.

30. Holbeck SL, Camalier R, Crowell JA, Govindharajulu JP, Hollingshead M, Anderson LW, et al.. The National Cancer Institute ALMANAC: A Comprehensive Screening Resource for the Detection of Anticancer Drug Pairs with Enhanced Therapeutic Activity. *Cancer Research*. 2017; doi: 10.1158/0008-5472.CAN-17-0489.

31. Shoemaker RH. The NCI60 human tumour cell line anticancer drug screen. *Nature Reviews Cancer*. 2006; doi: 10.1038/nrc1951.

32. DCTD. DTP, DCTD Tumor Repository - A catalog of in vitro cell lines, transplantable animal and human tumors and yeast. National Cancer Institute at Frederick; 2020 Jul.

33. Zheng S, Wang W, Aldahdooh J, Malyutina A, Shadbahr T, Pessia A, et al.. SynergyFinder Plus: towards a better interpretation and annotation of drug combination screening datasets. Bioinformatics; 2021 Jun.

34. Kim S, Chen J, Cheng T, Gindulyte A, He J, He S, et al.. PubChem 2019 update: improved access to chemical data. *Nucleic Acids Research*. 2018; doi: 10.1093/nar/gky1033.

35. Moriwaki H, Tian Y-S, Kawashita N, Takagi T. Mordred: a molecular descriptor calculator. *Journal of Cheminformatics*. 2018; doi: 10.1186/s13321-018-0258-y.

36. Pedregosa F, Varoquaux G, Gramfort A, Michel V, Thirion B, Grisel O, et al.. Scikit-learn: Machine Learning in Python. *Journal of Machine Learning Research*. 12:2825–302011;

37. Barretina J, Caponigro G, Stransky N, Venkatesan K, Margolin AA, Kim S, et al.. The Cancer Cell Line Encyclopedia enables predictive modelling of anticancer drug sensitivity. *Nature*. 2012; doi: 10.1038/nature11003.

38. Bairoch A. The Cellosaurus, a Cell-Line Knowledge Resource. *J Biomol Tech*. 2018; doi: 10.7171/jbt.18-2902-002.

39. Ghandi M, Huang FW, Jané-Valbuena J, Kryukov GV, Lo CC, McDonald ER, et al.. Next-generation characterization of the Cancer Cell Line Encyclopedia. *Nature*. 2019; doi: 10.1038/s41586-019-1186-3.

40. The International HapMap Consortium. The International HapMap Project. *Nature*. 2003; doi: 10.1038/nature02168.

41. Venkatraman ES, Olshen AB. A faster circular binary segmentation algorithm for the analysis of array CGH data. *Bioinformatics*. 2007; doi: 10.1093/bioinformatics/btl646.

42. Chaudhary K, Poirion OB, Lu L, Garmire LX. Deep Learning–Based Multi-Omics Integration Robustly Predicts Survival in Liver Cancer. *Clin Cancer Res*. American Association for Cancer Research; 2018; doi: 10.1158/1078-0432.CCR-17-0853.

43. Zhang L, Lv C, Jin Y, Cheng G, Fu Y, Yuan D, et al.. Deep Learning-Based Multi-Omics Data Integration Reveals Two Prognostic Subtypes in High-Risk Neuroblastoma. *Front Genet*. Frontiers; 2018; doi: 10.3389/fgene.2018.00477.

44. Simidjievski N, Bodnar C, Tariq I, Scherer P, Andres Terre H, Shams Z, et al.. Variational Autoencoders for Cancer Data Integration: Design Principles and Computational Practice. *Front Genet*. Frontiers; 2019; doi: 10.3389/fgene.2019.01205.

45. Hinton GE, Salakhutdinov RR. Reducing the Dimensionality of Data with Neural Networks. *Science*. American Association for the Advancement of Science; 2006; doi: 10.1126/science.1127647.

46. Wang Y, Yao H, Zhao S. Auto-encoder based dimensionality reduction. *Neurocomputing*. 2016; doi: 10.1016/j.neucom.2015.08.104.

47. Abadi M, Agarwal A, Barham P, Brevdo E, Chen Z, Citro C, et al.. TensorFlow: Large-Scale Machine Learning on Heterogeneous Distributed Systems. 2015;

48. Kingma DP, Ba J. Adam: A Method for Stochastic Optimization. *arXiv:14126980 [cs]*. 2017;

49. Meng C, Oana A. Zeleznik, Gerhard G. Thallinger, Bernhard Kuster, Amin M. Gholami, Aedín C. Culhane. Dimension reduction techniques for the integrative analysis of multi-omics data. *Briefings in Bioinformatics*. 2016; doi: 10.1093/bib/bbv108.

50. Preto AJ, Moreira IS. SPOTONE: Hot Spots on Protein Complexes with Extremely Randomized Trees via Sequence-Only Features. *IJMS*. 2020; doi: 10.3390/ijms21197281.

51. Botchkarev A. A New Typology Design of Performance Metrics to Measure Errors in Machine Learning Regression Algorithms. *IJIKM*. 2019; doi: 10.28945/4184.

52. de Winter JCF, Gosling SD, Potter J. Comparing the Pearson and Spearman correlation coefficients across distributions and sample sizes: A tutorial using simulations and empirical data. *Psychological Methods*. 2016; doi: 10.1037/met0000079.

53. Breiman L. Random Forests. *Machine Learning*. 45:5–322001;

54. Geurts P, Ernst D, Wehenkel L. Extremely randomized trees. *Machine Learning*. 63:3–422006;

55. Fan RE, Chang KW, Hsieh CJ, Wang XR, Lin CJ. LIBLINEAR: A library for large linear classification. *Journal of Machine Learning Research*. 9:1871–42008;

56. Zadrozny B, Elkan C. Transforming classifier scores into accurate multiclass probability estimates. *KDD '02: Proceedings of the eighth ACM SIGKDD international conference on Knowledge discovery and data mining*. 2002; doi: 10.1145/775047.775151.

57. Altman NS. An introduction to kernel and nearest-neighbor nonparametric regression. *The American Statistician*. 46:175–851992;

58. Chen T, Guestrin C. XGBoost: A Scalable Tree Boosting System. *Proceedings of the 22nd ACM SIGKDD International Conference on Knowledge Discovery and Data Mining*. 2016; doi: 10.1145/2939672.2939785.

59. Noemí DeCastro-García, Ángel Luis Muñoz Castañeda, David Escudero García, Miguel V. Carriegos. Effect of the Sampling of a Dataset in the Hyperparameter Optimization Phase over the Efficiency of a Machine Learning Algorithm. *Advances in Complex Systems and Their Applications to Cybersecurity*. 2019; doi: 10.1155/2019/6278908.

60. Swersky K, Snoek J, Adams RP. Multi-Task Bayesian Optimization. *NIPS'13: Proceedings of the 26th International Conference on Neural Information Processing Systems*. 2:2004–122013;

61. Mikhail Korobov, Konstantin Lopuhin. ELI5.

62. O'Neil J, Benita Y, Feldman I, Chenard M, Roberts B, Liu Y, et al.. An Unbiased Oncology Compound Screen to Identify Novel Combination Strategies. *Mol Cancer Ther*. 2016; doi: 10.1158/1535-7163.MCT-15-0843.

63. Forcina GC, Conlon M, Wells A, Cao JY, Dixon SJ. Systematic Quantification of Population Cell Death Kinetics in Mammalian Cells. *Cell Systems*. 2017; doi: 10.1016/j.cels.2017.05.002.

64. Licciardello MP, Ringler A, Markt P, Klepsch F, Lardeau C-H, Sdelci S, et al.. A combinatorial screen of the CLOUD uncovers a synergy targeting the androgen receptor. *Nat Chem Biol*. United States; 2017; doi: 10.1038/nchembio.2382.

65. Kumar V, Dogra N. A Comprehensive Review on Deep Synergistic Drug Prediction Techniques for Cancer. *Archives of Computational Methods in Engineering*. 2022; doi: 10.1007/s11831-021-09617-3.

66. Plotly Technologies Inc. Collaborative data science. Plotly. Montréal, QC: Plotly Technologies Inc. Collaborative data science;

67. Grinberg M. Flask web development: developing web applications with python. O'Reilly Media, Inc.;

68. Grapov D, Fahrmann J, Wanichthanarak K, Khoomrung S. Rise of Deep Learning for Genomic, Proteomic, and Metabolomic Data Integration in Precision Medicine. *OMICS: A Journal of Integrative Biology*. 2018; doi: 10.1089/omi.2018.0097.

69. Lin Y-H, Lim S-N, Chen C-Y, Chi H-C, Yeh C-T, Lin W-R. Functional Role of Mitochondrial DNA in Cancer Progression. *Int J Mol Sci*. MDPI; 2022; doi: 10.3390/ijms23031659.

70. Tian B-X, Sun W, Wang S-H, Liu P-J, Wang Y-C. Differential expression and clinical significance of COX6C in human diseases. *Am J Transl Res*. 13:1–102021;

71. Wu C-J, Cai T, Rikova K, Merberg D, Kasif S, Steffen M. A predictive phosphorylation signature of lung cancer. *PLoS One*. Public Library of Science; 2009; doi: 10.1371/journal.pone.0007994.

72. Wang B, Cai Y, Li X, Kong Y, Fu H, Zhou J. ETV4 mediated lncRNA C2CD4D-AS1 overexpression contributes to the malignant phenotype of lung adenocarcinoma cells via miR-3681-3p/NEK2 axis. *Cell Cycle*. 2021; doi: 10.1080/15384101.2021.2005273.

73. Wang L, Shilatifard A. UTX Mutations in Human Cancer. *Cancer Cell*. 2019; doi: 10.1016/j.ccell.2019.01.001.

74. Gozdecka M, Meduri E, Mazan M, Tzelepis K, Dudek M, Knights AJ, et al.. UTX-mediated enhancer and chromatin remodeling suppresses myeloid leukemogenesis through noncatalytic inverse regulation of ETS and GATA programs. *Nat Genet*. 2018; doi: 10.1038/s41588-018-0114-z.

75. Sakthianandeswaren A, Parsons MJ, Mouradov D, MacKinnon RN, Catimel B, Liu S, et al.. MACROD2 Haploinsufficiency Impairs Catalytic Activity of PARP1 and Promotes Chromosome Instability and Growth of Intestinal Tumors. *Cancer Discov*. United States; 2018; doi: 10.1158/2159-8290.CD-17-0909.

76. Fernandes S, Huellen K, Goncalves J, Dukal H, Zeisler J, Rajpert De Meyts E, et al.. High frequency of DAZ1/DAZ2 gene deletions in patients with severe oligozoospermia. *Molecular Human Reproduction*. 2002; doi: 10.1093/molehr/8.3.286.

77. Hanson HA, Anderson RE, Aston KI, Carrell DT, Smith KR, Hotaling JM. Subfertility increases risk of testicular cancer: evidence from population-based semen samples. *Fertil Steril*. 2015/11/18 ed. 2016; doi: 10.1016/j.fertnstert.2015.10.027.

78. Chen T, Wang K, Tong X. In vivo and in vitro inhibition of human gastric cancer progress by upregulating Kank1 gene. *Oncol Rep*. 2017; doi: 10.3892/or.2017.5823.

79. Gu Y, Zhang M. Upregulation of the Kank1 gene inhibits human lung cancer progression in�vitro and in�vivo. *Oncol Rep*. 2018; doi: 10.3892/or.2018.6526.

80. Pedersen A, Mikkelsen E, Cronin-Fenton D, Kristensen N, Pham TM, Pedersen L, et al.. Missing data and multiple imputation in clinical epidemiological research. *CLEP*. 2017; doi: 10.2147/CLEP.S129785.

81. Zhang Z. Missing data imputation: focusing on single imputation. *Ann Transl Med*. 2016; doi: 10.3978/j.issn.2305-5839.2015.12.38.

82. Gilvary C, Dry JR, Elemento O. Multi-task learning predicts drug combination synergy in cells and in the clinic. Cancer Biology; 2019 Mar.

83. Di Veroli GY, Fornari C, Wang D, Mollard S, Bramhall JL, Richards FM, et al.. Combenefit: an interactive platform for the analysis and visualization of drug combinations. *Bioinformatics*. 2016; doi: 10.1093/bioinformatics/btw230.

84. Akoglu H. User's guide to correlation coefficients. *Turkish Journal of Emergency Medicine*. 2018; doi: 10.1016/j.tjem.2018.08.001.

85. Sidorov P, Naulaerts S, Ariey-Bonnet J, Pasquier E, Ballester PJ. Predicting Synergism of Cancer Drug Combinations Using NCI ALMANAC Data. *Front Chem*. 2019; doi: 10.3389/fchem.2019.00509.

86. Preuer K, Lewis RPI, Hochreiter S, Bender A, Bulusu KC, Klambauer G. DeepSynergy: predicting anti-cancer drug synergy with Deep Learning. Wren J, editor. *Bioinformatics*. 2018; doi: 10.1093/bioinformatics/btx806.

87. Zagidullin B, Aldahdooh J, Zheng S, Wang W, Wang Y, Saad J, et al.. DrugComb: an integrative cancer drug combination data portal. *Nucleic Acids Res*. 2019; doi: 10.1093/nar/gkz337.

88. Holbeck SL, Camalier R, Crowell JA, Govindharajulu JP, Hollingshead M, Anderson LW, et al.. The National Cancer Institute ALMANAC: A Comprehensive Screening Resource for the Detection of Anticancer Drug Pairs with Enhanced Therapeutic Activity. *Cancer Res*. 2017; doi: 10.1158/0008-5472.CAN-17-0489.

89. Preto AJ, Matos-Filipe P, Mourão J, Moreira IS. Supporting data for "SYNPRED: Prediction of Drug Combination Effects in Cancer using Different Synergy Metrics and Ensemble Learning" GigaScience Database. 2022. http://dx.doi.org/10.5524/102255

figure 1

Click here to access/download;Figure;SynpredFig1UPDATED.png

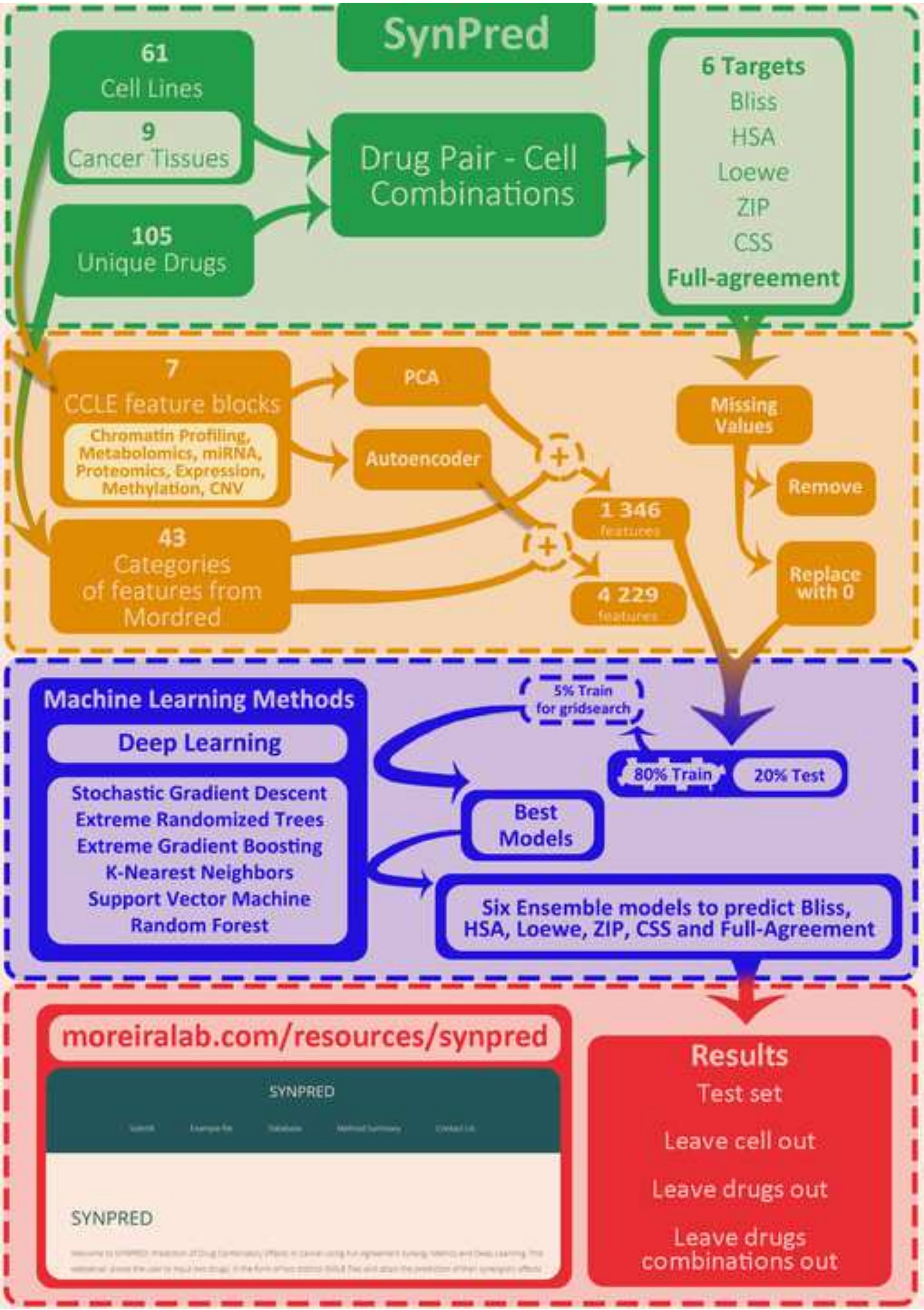

Click here to access/download;Figure;Figure_2.png

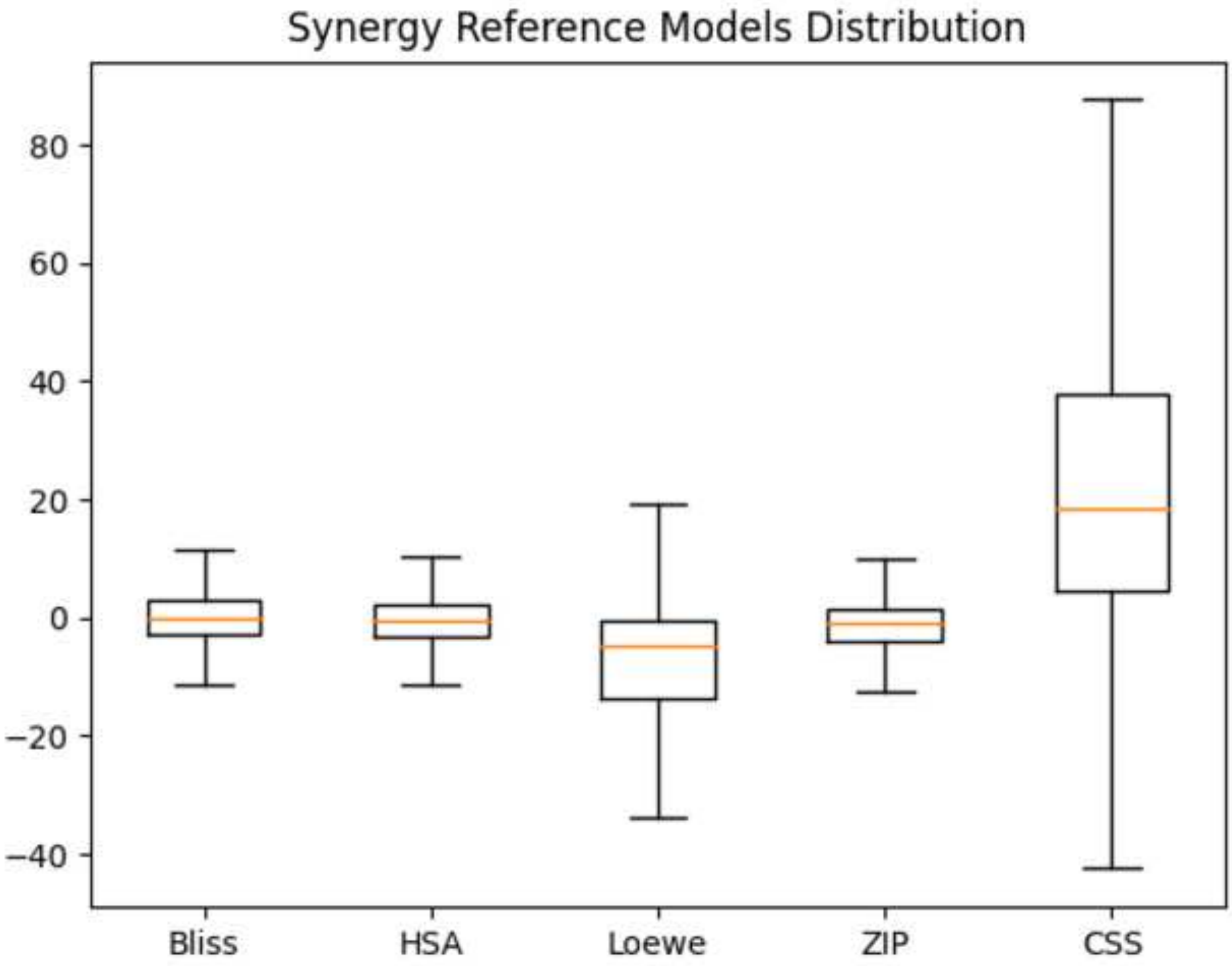

Click here to access/download;Figure;Figure_3.png

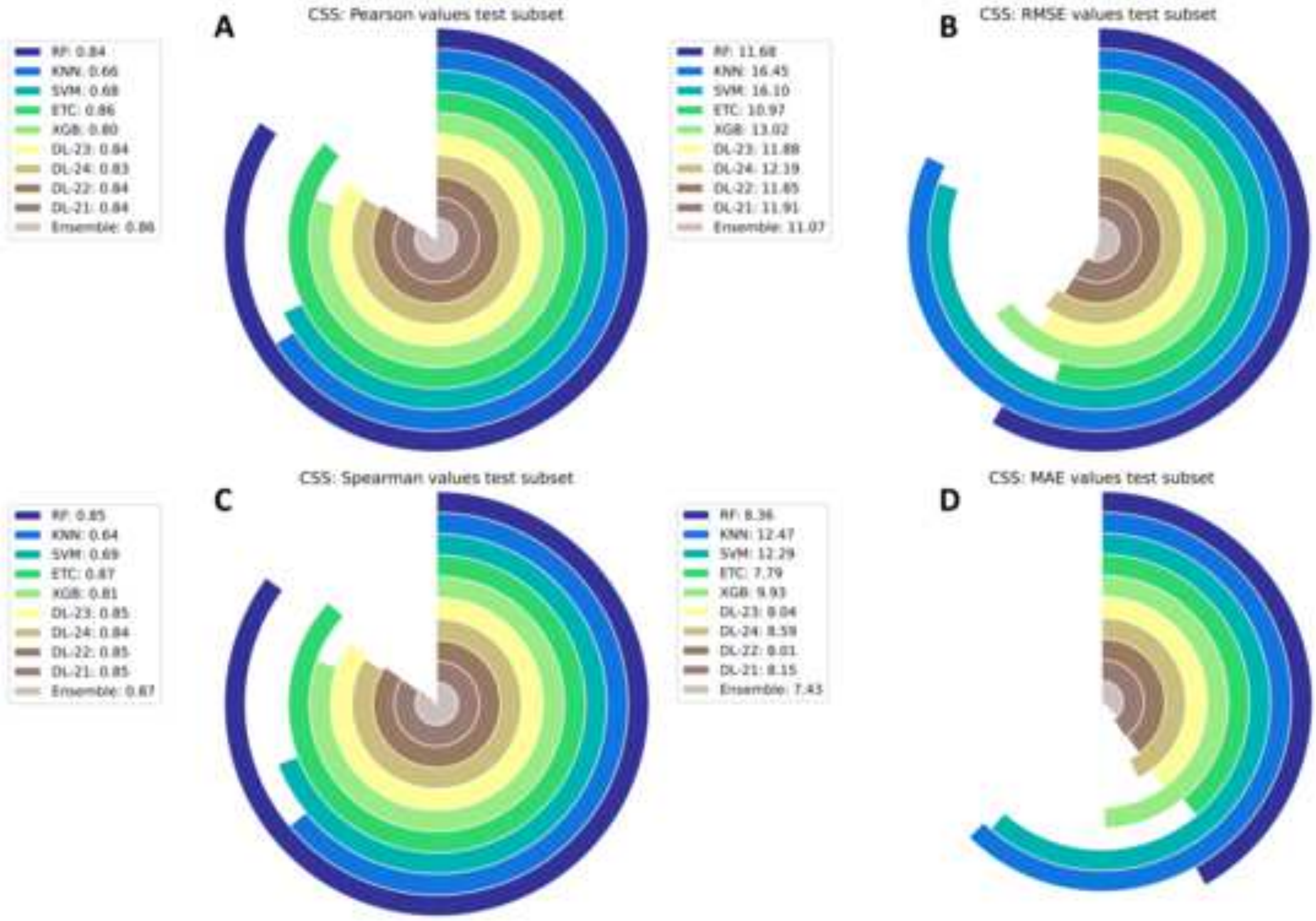

Click here to access/download;Figure;Figure_4.png

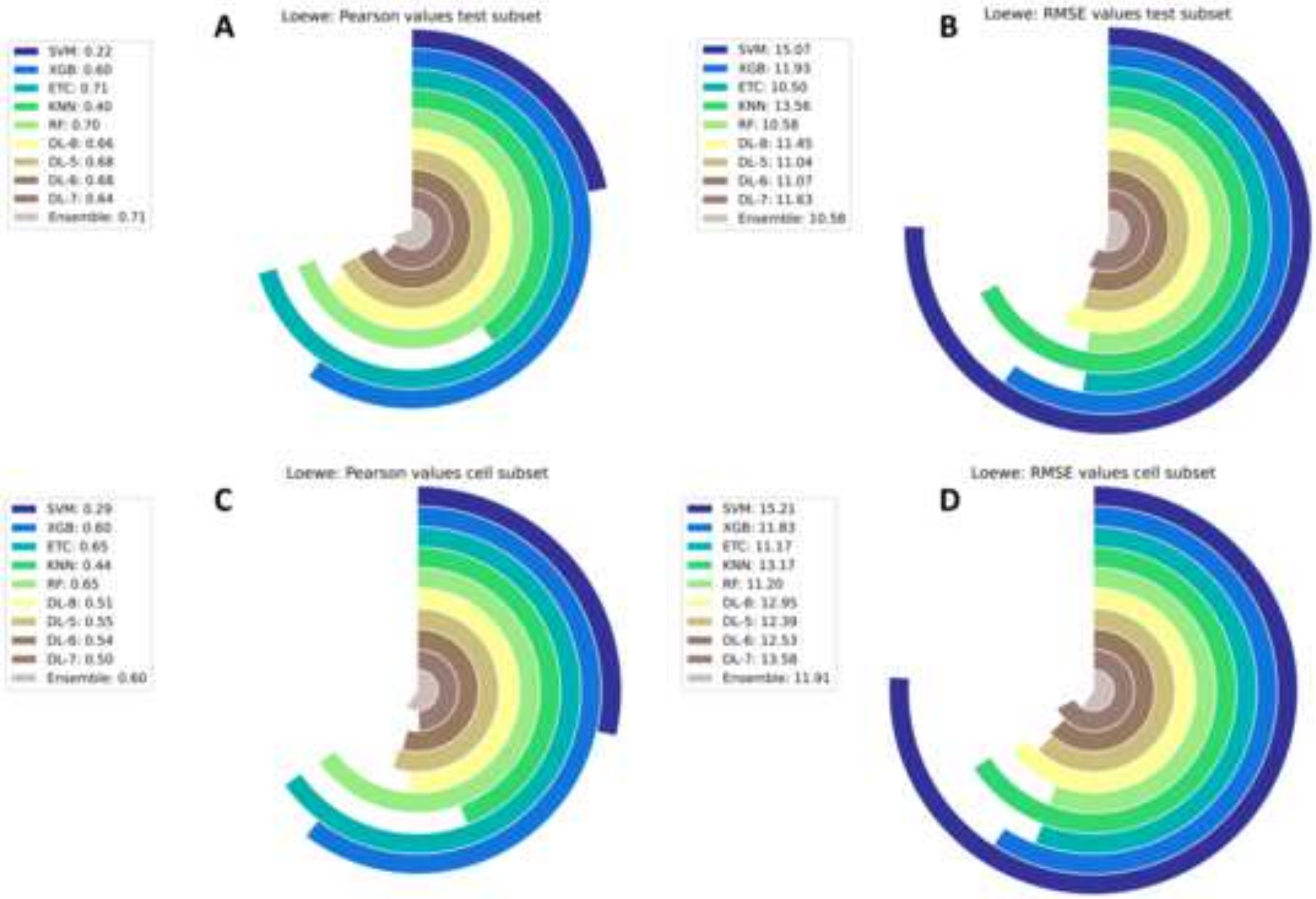

Click here to access/download;Figure;Figure_5.png

Your prediction for the input table of combinations yielded the results below according to the Deep Learning Ensemble of the Full-Agreement class and the four synergy reference models. Please not that if a cell line is marked as **UNK** it means it was not recognized by SynPred, hence, the prediction is based on drug features alone. The colorscale on the heatmap represents the synergy likelihood (the greener the cells, the more likely it is to be synergistic). The column added to the table ("Synergy Votes") reflects how many of the five predictors characterize your input samples as synergistic. You can also download the raw table in the button below. For more information, consult the paper, Preto, A.J. *et al.* 2022. Your results will be erased from our server after two weeks.

Download Table

Cell Line	Full-agreement	ZIP	HSA	Bliss	Loewe	CSS	Synergy Votes
MCF7	0.914	3.275	4.073	14.057	-0.413	54.161	5
CCRFCEM	0.996	-0.428	-0.335	-0.072	-3.56	5.044	2
MOLT4	0.998	2.357	-16.273	-2.844	26.068	4.928	4

suppl info

Click here to access/download
Supplementary Material
Synpred-SI-R3.docx

Click here to access/download;Personal Cover;Answers-SYNPRED_R3.docx

Coimbra, 2h August 2022

RE: Manuscript ID No.: GIGA-D-21-00121

Dear Prof. Hans Zauner,

Thank you for handling our manuscript throughout this process.

All changes to the manuscript are marked in red. The answers to the reviewer's specific comments are included below. We hope that our manuscript SYNPRED: Prediction of Drug Combination Effects in Cancer using Different Synergy Metrics and Ensemble Learning" fulfils now the necessary quality criteria to be published at GigaScience.

Yours sincerely,

Irina S. Moreira, Ph.D.

Reviewer #1: Authors indicate that benchmarking of synergy prediction protocols is a complicated process. While I agree with this. , I do not agree that "..DL architectures as these are not easily applied or not available in GitHub or similar platforms." As the implementations are available on GitHub, the authors can feed their data of choice to easily train these models. There might be some adaptation choices to make such as pruning the data due to unavailability of a certain data modality, or changing the loss function to turn a model into a regressor, but as long as they justify these it should be fine and east. This is what has been done in the literature in DeepSynergy and Matchmaker papers, for instance. They have done this to a degree but I am not convinced that a fair comparison with the literature I made to conclude that this method has clear advantages over others. Authors indicate that they trained 1972 models but I think these are mostly the "baseline" models they use within their ensemble method which does not help in comparing with the literature. Below, I list my detailed comments on the revisions made:

First, while there are many detailed descriptive statistics such as distribution of synergy scores are presented in the main text the comparisons are pushed to the supplement which makes it hard to follow the paper. Also, details on whether or how compared methods are run/trained is missing. For instance in comparison ii), how did you reimplement DeepSynergy, and in Comparison ii) and iv) what data did you use and what hyperparameters did you use to run DeepSynergy and Matchmaker? In the current revision, in Tables S7 - S12 authors compare their method with some baseline models used in their ensemble such as random forest, KNN and

some custom deep learning architectures. I did not ask for this as clearly the problem is complex and a complex model as their method is needed. Nevertheless this is a nice validation which improves the paper. They compare their method with a reimplemented (please clarify in text) version of DeepSynergy in Table S13 (They say Table S14 but I think it is a typo).

We have corrected the typo and changed it to **Table 13_Supplementary Material.**

As thoroughly discussed, there is a fundamental disagreement between the authors' and the reviewer on this matter. A correct benchmark on this problem is not currently feasible, as we have attempted to make it clear to the reviewer. The benchmarking problem is evidenced by the publication of ChemicalX (Rozemberczki et al. 2022), in which DeepSynergy, MatchMaker, among others algorithm are re-implemented. This work was published in 2022 by several researchers. The fact that they had to re-implement the algorithms shows what we have stated for several versions of the answers to the reviewer: the present state of the art does not allow for proper benchmark. Unfortunately, ChemicalX only provides a binary class for drug synergy prediction.

However, we deployed a multi-step approach that compares individually either datasets, Machine Learning deployments or literature referenced results. We indeed benchmarked our pipeline as described in the methods section:

i) Comparison of the best performing individual DL and ML algorithms with the ensemble approaches for each prediction task - Tables 7 to 12_Supplementary Material.

ii) DeepSynergy architecture implementation and comparison using our independent test set and validation sets - Table 13_Supplementary Material.

iii) Comparison with published methods for synergy calculations as reviewed by Kumar et al. - Table 14_Supplementary Material.

iv) Comparison of our regression approaches to Matchmakers' algorithm, DeepSynergy and TreeCombo - Tables 15 and 16_Supplementary Material.

First, I strongly suggest they put the result of the final method into this table (and other related ones) for the readability. I had to trace Table 4/5 to see their results and come back to compare with the values in this table. They report better results than DeepSynergy which is good. As they use the review of Kumar et al. later, there are other methods reported in this review which perform better than DeepSynergy in the regression task such as MultitaskDNN, AUDNNSynergy,

and Matchmaker. However only DeepSynergy is compared. Later they perform a comparison with Matchmaker but it is ot clear to me why they had to perform a separate comparison instead of comparing them all. In supplementary table 14, they compare the performance of their method, with performance values of others reported in a review by Kumar et al. This is not meaningful at all as every method use different settings as authors also indicate. Once again, they have to retrain these (or similar) architectures with their (maybe filtered or postprocessed) data and compare. Just like done for DeepSynergy only in the regression task. They can as well retrain their method with the dataset of these methods as another point of view. For the results in Tables S15 - S16 (again the table numbers are wrong), author state "... Upon doing this, both CSS and Loewe predictors from SynPred stood very close to the performance of Matchmaker [20],". Matchmaker reports Loewe scores so if no retraining of the model is performed with CSS it is not meaningful to compare these results. I suggest authors to use the CSS scores as the label in training Matchmaker. In Loewe scores their performance actually does not stand vey close to Matchmaker (MSE vs 123, Pearson .79 vs .73). Only in Spearman correlation their result is 0.01 better. *In Table S16, SynPred seems to be 50 times better than all others in terms of MSE. However, while SynPred reports various scores other than ComboScore, the results obtained for other methods are based on ComboScore. Thus, it is comparing apples and oranges and is not meaningful. Again, no training is performed in this comparison. Please do not put results with different metrics into the same table as it is very confusing for the reader.*

The complexity of the benchmark problem is thoroughly addressed throughout the manuscript, and it cannot be reduced to a method against method comparison.

We have reimplemented DeepSynergy the same way the other authors that claim do benchmark DeepSynergy did, by reimplementing a neural network with the same dimensions and hyperparameters although on other data. We also compared the results of our predictor on Matchmakers' dataset and the results attained by these authors. The clarifications regarding implementation are now in page 16 of the revised manuscript.

Table S16 displays several predictors, many of which do not have ComboScore as target. We clearly state which SynPred predictors we are referring to by adding the name of target variable in front of SynPred.

We did expand our comments MSE, precisely because we are aware that error derived metrics are particularly hard to compare between datasets and methods. Clarified in page 26 of the revised manuscript.

We do not believe that adding table 4 and 5 in some of supplementary tables will increase readability, just the opposite and we did not follow this suggestion.

We have changed the numbering of tables as indeed these were incorrect.

The details on the dataset used, the train/validation/test splits and number of samples etc. are missing so it is hard to evaluate these scores.

This statement is purely wrong, all requested data is available on Table 3_Supplementary Material and detailed in text (subsection “Model evaluation and performance metrics”, lines 252-276).

TYPOs: NCI ALMANAC, Matchmakers'

We have followed reviewer advice and changed the two types: NCI-ALMANC towards NCI ALMANAC and Matchmaker to Matchmakers' in the main document as well as in SI.
