# Supplementary material for: SYNPRED: prediction of drug combination effects in cancer using different synergy metrics and ensemble learning
Source: Gigascience. 2022 Sep 26;11:giac087. doi: 10.1093/gigascience/giac087 (PMC9511701; doi:10.1093/gigascience/giac087)
Supplement: giac087_Supplemental_File [file giac087_supplemental_file.docx]

**SYNPRED: Prediction of Drug Combination Effects in Cancer using Different Synergy Metrics and Ensemble Learning**

António J. Preto^1,2^, Pedro Matos-Filipe^1^, Joana Mourão^1^ and Irina S. Moreira^3,4*^

^1^University of Coimbra, Center for Neuroscience and Cell Biology, 3004-504 Coimbra, Portugal

^2^PhD Programme in Experimental Biology and Biomedicine, Institute for Interdisciplinary Research (IIIUC), University of Coimbra, Casa Costa Alemão, [3030-789](tel:3030-789) Coimbra, Portugal

^3^University of Coimbra, Department of Life Sciences, Calçada Martim de Freitas, 3000-456 Coimbra, Portugal

^4^CNC - Center for Neuroscience and Cell Biology, CIBB - Center for Innovative Biomedicine and Biotechnology, 3004-504 Coimbra, Portugal

* To whom correspondence should be addressed.

Tel: (+351) 239 240 227

Email: [irina.moreira@cnc.uc.pt](mailto:irina.moreira@cnc.uc.pt)

**SUPPLEMENTARY INFORMATION**

**Table 1.** Conditions for dimensionality reduction with autoencoders. Hidden and bottleneck layers definition according to the Number of Features.

| **Number of Features (NoF)** | **Hidden layers size** | **Bottleneck layer size** |
| --- | --- | --- |
| $NoF\leq250$ | HL1: $NoF*2$ | $\frac{NoF}{2}$ |
|  | HL2: $NoF$ |  |
|  | HL4: $NoF$ |  |
|  | HL5: $NoF*2$ |  |
| $250< NoF\leq1000$ | HL1: $NoF$ | $\frac{NoF}{10}$ |
|  | HL2: $\frac{NoF}{2}$ |  |
|  | HL4: $\frac{NoF}{2}$ |  |
|  | HL5: $NoF$ |  |
| $NoF\geq1000$ | HL1: $\frac{NoF}{2}$ | $\frac{NoF}{50}$ |
|  | HL2: $\frac{NoF}{4}$ |  |
|  | HL4: $\frac{NoF}{4}$ |  |
|  | HL5: $\frac{NoF}{2}$ |  |

**Table 2.** Conditions for dimensionality reduction with autoencoders. Number of epochs of the autoencoder training according to either the Number of Samples or Number of Features.

| **Number of Samples (NoS)** | **Number of Features (NoF)** | **Number of epochs** |
| --- | --- | --- |
| $NoS\leq100$ | $NoF\leq100$ | 1000 |
| $100<NoS<1000$ | $100< NoF\leq250$ | 250 |
| $NoS\leq1000$ | $NoF\geq250$ | 100 |

**Table 3.** Final datasets to be subjected to training.

| **Dataset name** | **Feature pre-processing** | **Feature’s size** | **Sample pre-processing** |  | **Random sample split** | |
| --- | --- | --- | --- | --- | --- | --- |
| PCA | PCA | 1.347 | Replace missing values with 0 | **Train:** 195.996  **Test:** 48.999  **Leave cell:** 13.810  **Leave drugs:** 25.993  **Leave drug combinations:** 360 | |  |
|  |  |  |  |  |  |  |
| Autoencoder | Autoencoder | 4.229 |  |  |  |  |
| PCA | PCA | 1.347 | Remove rows with missing values | **Train:** 118.079  **Test:** 29.756  **Leave cell:** 5.210  **Leave drugs:** 15.723  **Leave drug combinations:** 219 | |  |
|  |  |  |  |  |  |  |
| Autoencoder | Autoencoder | 4.229 |  |  |  |  |

**Table 4.** Gridsearch combination parameters using 5% on the training set with Deep Learning algorithms.

| **Dataset name** | **Architecture** | **Dropout rate** |
| --- | --- | --- |
| PCA  PCA_drop  Autoencoder  Autoencoder_drop | (100,100,100), (100,100,100,100), (500,500,500), (500,500,500,500), (1000,1000,1000), (1000,1000,1000,1000), (2500,2500,2500), (2500,2500,2500,2500), (673,336,84), (673,336,84,5), (2114,1057,264), (2114,1057,264,16) | 0.00, 0.25, 0.50, 0.75 |
|  |  |  |

**Table 5.** Gridsearch combination parameters using 5% on the training set with non-Deep Learning algorithms.

| **Method** | **Parameters** | **Dataset name** |
| --- | --- | --- |
| RF  (RandomForestClassifier) | n_estimators: (10,100,1000)  max_depth: (None, 1, 5)  min_samples_split: (2, 5,10)  min_samples_leaf: (1, 2, 4) | PCA, PCA_drop, autoencoder, autoencoder_drop |
| ETC  (ExtraTreesClassifier) | n_estimators: (10,100,1000)  min_samples_split: (2, 5,10)  max_depth: (None, 1, 5)  min_samples_leaf: (1, 2, 4) |  |
| SVM  (LinearSVC) | C: (0.1,0.5,1.0) |  |
| SGD  (SGDClassifier) | penalty: (l2, l1, elasticnet)  alpha: (0.00001, 0.0001, 0.001) |  |
| KNN  (KNeighborsClassifier) | n_neighbors: (2, 5, 10, 25) |  |
| XGB  (XGBClassifier) | max_depth: (2, 6, 10)  alpha: (0, 0.25, 0.50)  n_estimators: (10, 100, 1000) |  |

**Table 6.** Gridsearch combination parameters of the ensemble neural network.

| **Architecture** | **Dropout rate** |
| --- | --- |
| (10,10,10), (10,10,10,10,10), (10,10,10,10,10,10,10), (50,50,50), (50,50,50,50,50), (50,50,50,50,50,50,50), (100,100,100), (100,100,100,100,100), (100,100,100,100,100,100,100), (250,250,250), (250,250,250,250,250), (250,250,250,250,250,250,250), (500,500,500), (500,500,500,500,500), (500,500,500,500,500,500,500) | 0.00, 0.30, 0.60, 0.90 |

**Table 7.** Final metrics of the classification models evaluated in an independent test set and three different scenarios (leave cell out, leave drugs out and leave drug combinations out) using Full-Agreement synergy values.

| Model | Subset used for evaluation | Acc | Prec | Rec | AUROC | F1 |
| --- | --- | --- | --- | --- | --- | --- |
| ENS | Test | 0.85 | 0.91 | 0.90 | 0.80 | 0.90 |
|  | Leave cells out | 0.37 | 0.89 | 0.13 | 0.55 | 0.22 |
|  | Leave drugs out | 0.33 | 0.86 | 0.13 | 0.53 | 0.22 |
|  | Leave drug combinations out | 0.24 | 1.00 | 0.21 | 0.61 | 0.35 |
| ETC *^a^* | Test | 0.84 | 0.88 | 0.92 | 0.76 | 0.90 |
|  | Leave cells out | 0.29 | 0.12 | 0.93 | 0.57 | 0.21 |
|  | Leave drugs out | 0.21 | 0.12 | 0.92 | 0.52 | 0.21 |
|  | Leave drug combinations out | 0.35 | 0.24 | 1.00 | 0.59 | 0.39 |
| KNN *^a^* | Test | 0.78 | 0.80 | 0.95 | 0.58 | 0.87 |
|  | Leave cells out | 0.24 | 0.11 | 0.91 | 0.53 | 0.19 |
|  | Leave drugs out | 0.18 | 0.12 | 0.95 | 0.52 | 0.21 |
|  | Leave drug combinations out | 0.26 | 0.21 | 0.96 | 0.52 | 0.35 |
| RF *^a^* | Test | 0.86 | 0.88 | 0.94 | 0.76 | 0.91 |
|  | Leave cells out | 0.27 | 0.12 | 0.94 | 0.57 | 0.21 |
|  | Leave drugs out | 0.20 | 0.12 | 0.97 | 0.53 | 0.21 |
|  | Leave drug combinations out | 0.26 | 0.22 | 1.00 | 0.53 | 0.36 |
| SGD *^a^* | Test | 0.78 | 0.82 | 0.91 | 0.63 | 0.86 |
|  | Leave cells out | 0.20 | 0.11 | 0.95 | 0.53 | 0.19 |
|  | Leave drugs out | 0.11 | 0.11 | 1.00 | 0.50 | 0.20 |
|  | Leave drug combinations out | 0.35 | 0.24 | 1.00 | 0.59 | 0.39 |
| SVM *^a^* | Test | 0.71 | 0.82 | 0.81 | 0.61 | 0.81 |
|  | Leave cells out | 0.20 | 0.11 | 0.97 | 0.54 | 0.20 |
|  | Leave drugs out | 0.26 | 0.12 | 0.85 | 0.52 | 0.21 |
|  | Leave drug combinations out | 0.33 | 0.22 | 0.91 | 0.54 | 0.36 |
| XGB *^a^* | Test | 0.86 | 0.89 | 0.93 | 0.77 | 0.91 |
|  | Leave cells out | 0.29 | 0.12 | 0.91 | 0.57 | 0.21 |
|  | Leave drugs out | 0.25 | 0.12 | 0.93 | 0.54 | 0.22 |
|  | Leave drug combinations out | 0.32 | 0.23 | 1.00 | 0.57 | 0.38 |
| DL1 *^b^* | Test | 0.83 | 0.90 | 0.87 | 0.77 | 0.88 |
|  | Leave cells out | 0.32 | 0.10 | 0.76 | 0.51 | 0.18 |
|  | Leave drugs out | 0.33 | 0.13 | 0.84 | 0.55 | 0.22 |
|  | Leave drug combinations out | 0.36 | 0.24 | 1.00 | 0.60 | 0.39 |
| DL2 *^b^* | Test | 0.83 | 0.88 | 0.90 | 0.75 | 0.89 |
|  | Leave cells out | 0.35 | 0.12 | 0.87 | 0.58 | 0.21 |
|  | Leave drugs out | 0.29 | 0.12 | 0.87 | 0.54 | 0.22 |
|  | Leave drug combinations out | 0.35 | 0.24 | 1.00 | 0.59 | 0.39 |
| DL3 *^b^* | Test | 0.82 | 0.88 | 0.89 | 0.75 | 0.89 |
|  | Leave cells out | 0.41 | 0.13 | 0.84 | 0.60 | 0.22 |
|  | Leave drugs out | 0.31 | 0.13 | 0.86 | 0.55 | 0.22 |
|  | Leave drug combinations out | 0.37 | 0.24 | 0.99 | 0.60 | 0.39 |
| DL4 *^b^* | Test | 0.83 | 0.86 | 0.92 | 0.72 | 0.89 |
|  | Leave cells out | 0.22 | 0.11 | 0.90 | 0.53 | 0.19 |
|  | Leave drugs out | 0.23 | 0.12 | 0.92 | 0.53 | 0.21 |
|  | Leave drug combinations out | 0.28 | 0.22 | 1.00 | 0.55 | 0.37 |

*^a^* ML-based model architectures include: ETC (max_depth: None, min_samples_leaf: 2, min_samples_split: 10, n_estimators: 10), KNN (n_neighbors: 25), RF (max_depth: None, min_samples_leaf: 2, min_samples_split: 2, n_estimators: 100), SGD (alpha: 0.001, penalty: elasticnet), SVM (C: 0.1), and XGB (alpha: 0.25, max_depth: 6, n_estimators:100).

*^b^* DL-based model architectures include: DL1 ([100, 100, 100], 0.25, PCA_fillna), DL2 ([673, 336, 84, 5], 0.25, PCA_fillna), DL3 ([500, 500, 500, 500, 500], 0.25, PCA_fillna) and DL4 ([2500, 2500, 2500, 2500], 0.25, PCA_fillna).

**Table 8.** Final metrics of the regression models evaluated in an independent test set and three different scenarios (leave cell out, leave drugs out and leave drug combinations out) using Bliss synergy reference model.

| Model *^a^* | Subset used for evaluation | RMSE | MSE | | Pearson | | MAE | | | Spearman | |
| --- | --- | --- | --- | --- | --- | --- | --- | --- | --- | --- | --- |
| ENS | Test | 4.35 | 18.92 | 0.71 | |  | | 3.07 | 0.59 | |  |
|  | Leave cells out | 5.41 | 29.29 | 0.52 | |  | | 3.89 | 0.43 | |  |
|  | Leave drugs out | 6.04 | 36.51 | 0.46 | |  | | 4.36 | 0.40 | |  |
|  | Leave drug combinations out | 8.10 | 65.54 | 0.20 | |  | | 6.10 | 0.19 | |  |
| ETC *^b^* | Test | 4.46 | 19.88 | 0.69 | |  | | 3.14 | 0.58 | |  |
|  | Leave cells out | 5.17 | 26.73 | 0.57 | |  | | 3.68 | 0.48 | |  |
|  | Leave drugs out | 5.60 | 31.33 | 0.54 | |  | | 4.04 | 0.46 | |  |
|  | Leave drug combinations out | 6.56 | 43.02 | 0.17 | |  | | 4.92 | 0.10 | |  |
| KNN *^b^* | Test | 5.71 | 32.55 | 0.38 | |  | | 3.88 | 0.35 | |  |
|  | Leave cells out | 5.85 | 34.21 | 0.37 | |  | | 4.14 | 0.32 | |  |
|  | Leave drugs out | 6.12 | 37.40 | 0.36 | |  | | 4.42 | 0.31 | |  |
|  | Leave drug combinations out | 6.20 | 38.39 | 0.24 | |  | | 4.62 | 0.18 | |  |
| RF *^b^* | Test | 4.51 | 20.33 | 0.68 | |  | | 3.18 | 0.56 | |  |
|  | Leave cells out | 5.59 | 31.20 | 0.47 | |  | | 3.97 | 0.35 | |  |
|  | Leave drugs out | 6.16 | 37.89 | 0.35 | |  | | 4.40 | 0.31 | |  |
|  | Leave drug combinations out | 6.23 | 38.86 | 0.06 | |  | | 4.63 | 0.03 | |  |
| SVM *^b^* | Test | 6.90 | 47.61 | 0.14 | |  | | 4.95 | 0.15 | |  |
|  | Leave cells out | 6.09 | 37.06 | 0.28 | |  | | 4.27 | 0.27 | |  |
|  | Leave drugs out | 8.04 | 64.57 | 0.08 | |  | | 6.08 | 0.07 | |  |
|  | Leave drug combinations out | 7.51 | 56.45 | -0.06 | |  | | 5.65 | -0.03 | |  |
| XGB *^b^* | Test | 5.31 | 28.22 | 0.53 | |  | | 3.70 | 0.38 | |  |
|  | Leave cells out | 5.59 | 31.20 | 0.47 | |  | | 3.97 | 0.35 | |  |
|  | Leave drugs out | 6.16 | 37.89 | 0.35 | |  | | 4.40 | 0.31 | |  |
|  | Leave drug combinations out | 6.23 | 38.86 | 0.06 | |  | | 4.63 | 0.03 | |  |
| DL9 *^c^* | Test | 4.65 | 21.62 | 0.67 | |  | | 3.30 | 0.54 | |  |
|  | Leave cells out | 5.58 | 31.17 | 0.48 | |  | | 4.01 | 0.36 | |  |
|  | Leave drugs out | 6.50 | 42.30 | 0.39 | |  | | 4.73 | 0.34 | |  |
|  | Leave drug combinations out | 7.61 | 57.96 | 0.22 | |  | | 5.70 | 0.22 | |  |
| DL10 *^c^* | Test | 5.05 | 25.47 | 0.60 | |  | | 3.59 | 0.46 | |  |
|  | Leave cells out | 6.20 | 38.49 | 0.36 | |  | | 4.44 | 0.28 | |  |
|  | Leave drugs out | 7.52 | 56.53 | 0.29 | |  | | 5.42 | 0.26 | |  |
|  | Leave drug combinations out | 8.09 | 65.50 | 0.12 | |  | | 5.93 | 0.13 | |  |
| DL11 *^c^* | Test | 4.73 | 22.37 | 0.66 | |  | | 3.36 | 0.53 | |  |
|  | Leave cells out | 5.65 | 31.96 | 0.47 | |  | | 4.05 | 0.37 | |  |
|  | Leave drugs out | 6.49 | 42.06 | 0.40 | |  | | 4.68 | 0.34 | |  |
|  | Leave drug combinations out | 10.91 | 118.93 | 0.20 | |  | | 7.35 | 0.27 | |  |
| DL12 *^c^* | Test | 4.91 | 24.07 | 0.64 | |  | | 3.49 | 0.51 | |  |
|  | Leave cells out | 6.09 | 37.09 | 0.40 | |  | | 4.45 | 0.29 | |  |
|  | Leave drugs out | 6.78 | 46.02 | 0.36 | |  | | 4.98 | 0.31 | |  |
|  | Leave drug combinations out | 12.99 | 168.65 | 0.07 | |  | | 8.52 | 0.12 | |  |
| DL13 *^c^* | Test | 5.14 | 26.42 | 0.59 | |  | | 3.61 | 0.46 | |  |
|  | Leave cells out | 6.37 | 40.56 | 0.34 | |  | | 4.59 | 0.24 | |  |
|  | Leave drugs out | 7.36 | 54.09 | 0.31 | |  | | 5.35 | 0.25 | |  |
|  | Leave drug combinations out | 12.21 | 149.02 | 0.14 | |  | | 7.96 | 0.19 | |  |

*^a^* The metrics of the worst SGD-based model were not included in the table since they were out of range compared with the other DL and non-DL models.

*^b^* ML-based model architectures include: ETC (max_depth: None, min_samples_leaf: 4, min_samples_split: 10, n_estimators: 1000), KNN (n_neighbors: 25), RF (max_depth: None, min_samples_leaf: 1, min_samples_split: 5, n_estimators: 1000), SVM (C: 0.1), and XGB (alpha: 0.5, max_depth: 6, n_estimators: 10).

*^c^* DL-based model architectures include: DL9 ([1000, 1000, 1000, 1000], 0.0, PCA_fillna), DL10 ([100, 100, 100, 100], 0.0, PCA_fillna), DL11 ([2114, 1057, 264], 0.0, PCA_fillna), DL12 ([673, 336, 84], 0.0, PCA_fillna) and DL13 ([100, 100, 100], 0.0, PCA_fillna).

**Table 9.** Final metrics of the regression models evaluated in an independent test set and three different scenarios (leave cell out, leave drugs out and leave drug combinations out) using HSA synergy reference model.

| Model*^a^* | Subset used for evaluation | RMSE | MSE | Pearson | MAE | Spearman |
| --- | --- | --- | --- | --- | --- | --- |
| ENS | Test | 4.09 | 16.70 | 0.73 | 2.86 | 0.64 |
|  | Leave cells out | 5.17 | 26.76 | 0.53 | 3.67 | 0.46 |
|  | Leave drugs out | 5.84 | 34.05 | 0.42 | 4.20 | 0.38 |
|  | Leave drug combinations out | 7.03 | 49.47 | 0.37 | 5.23 | 0.36 |
| ETC *^b^* | Test | 4.28 | 18.36 | 0.70 | 2.97 | 0.62 |
|  | Leave cells out | 5.04 | 25.36 | 0.56 | 3.53 | 0.48 |
|  | Leave drugs out | 5.59 | 31.26 | 0.45 | 4.02 | 0.38 |
|  | Leave drug combinations out | 6.53 | 42.69 | 0.51 | 4.67 | 0.46 |
| KNN *^b^* | Test | 5.49 | 30.11 | 0.41 | 3.70 | 0.37 |
|  | Leave cells out | 5.58 | 31.17 | 0.39 | 3.90 | 0.35 |
|  | Leave drugs out | 5.93 | 35.17 | 0.33 | 4.31 | 0.27 |
|  | Leave drug combinations out | 7.08 | 50.13 | 0.40 | 5.29 | 0.37 |
| RF *^b^* | Test | 4.36 | 19.05 | 0.69 | 3.03 | 0.60 |
|  | Leave cells out | 5.07 | 25.74 | 0.55 | 3.55 | 0.47 |
|  | Leave drugs out | 5.77 | 33.28 | 0.39 | 4.10 | 0.35 |
|  | Leave drug combinations out | 6.60 | 43.57 | 0.56 | 4.73 | 0.49 |
| SVM *^b^* | Test | 6.31 | 39.75 | 0.20 | 4.38 | 0.23 |
|  | Leave cells out | 5.81 | 33.76 | 0.30 | 4.00 | 0.31 |
|  | Leave drugs out | 7.31 | 53.43 | 0.14 | 5.51 | 0.13 |
|  | Leave drug combinations out | 6.91 | 47.68 | 0.24 | 5.09 | 0.25 |
| XGB *^b^* | Test | 4.86 | 23.58 | 0.59 | 3.38 | 0.49 |
|  | Leave cells out | 5.26 | 27.65 | 0.50 | 3.73 | 0.42 |
|  | Leave drugs out | 5.81 | 33.77 | 0.37 | 4.16 | 0.31 |
|  | Leave drug combinations out | 6.66 | 44.31 | 0.59 | 4.83 | 0.52 |
| DL14 *^c^* | Test | 4.41 | 19.49 | 0.69 | 3.10 | 0.58 |
|  | Leave cells out | 5.43 | 29.49 | 0.47 | 3.87 | 0.37 |
|  | Leave drugs out | 6.46 | 41.68 | 0.36 | 4.69 | 0.31 |
|  | Leave drug combinations out | 10.81 | 116.93 | 0.19 | 7.32 | 0.30 |
| DL15 *^c^* | Test | 4.77 | 22.71 | 0.63 | 3.36 | 0.51 |
|  | Leave cells out | 7.00 | 48.96 | 0.29 | 4.94 | 0.25 |
|  | Leave drugs out | 7.18 | 51.49 | 0.28 | 5.24 | 0.24 |
|  | Leave drug combinations out | 11.22 | 126.04 | 0.13 | 7.47 | 0.28 |
| DL16 *^c^* | Test | 4.53 | 20.49 | 0.67 | 3.18 | 0.57 |
|  | Leave cells out | 5.56 | 30.86 | 0.46 | 3.99 | 0.38 |
|  | Leave drugs out | 6.52 | 42.54 | 0.36 | 4.74 | 0.32 |
|  | Leave drug combinations out | 11.52 | 132.63 | 0.15 | 7.60 | 0.31 |
| DL17 *^c^* | Test | 4.55 | 20.66 | 0.67 | 3.20 | 0.56 |
|  | Leave cells out | 5.57 | 31.05 | 0.45 | 4.01 | 0.35 |
|  | Leave drugs out | 6.53 | 42.63 | 0.35 | 4.78 | 0.30 |
|  | Leave drug combinations out | 10.08 | 101.61 | 0.26 | 7.02 | 0.33 |

*^a^* The metrics of the worst SGD-based model were not included in the table since they were out of range compared with the other DL and non-DL models.

*^b^* ML-based model architectures include: ETC (max_depth: None, min_samples_leaf: 4, min_samples_split: 2, n_estimators: 1000), KNN (n_neighbors: 25), RF (max_depth: None, min_samples_leaf: 2, min_samples_split: 2, n_estimators: 1000), SVM (C: 0.1), and XGB (alpha: 0, max_depth: 2, n_estimators: 100).

*^c^* DL-based model architectures include: DL14 ([2114, 1057, 264, 16], 0.0, PCA_fillna), DL15 ([100, 100, 100, 100], 0.0, PCA_fillna), DL16 ([673, 336, 84, 5], 0.0, PCA_fillna), and DL17 ([673, 336, 84], 0.0, PCA_fillna).

**Table 10.** Final metrics of the regression models evaluated in an independent test set and three different scenarios (leave cell out, leave drugs out and leave drug combinations out) using Loewe synergy reference model.

| Model *^a^* | Subset used for evaluation | RMSE | MSE | Pearson | MAE | Spearman |
| --- | --- | --- | --- | --- | --- | --- |
| ENS | Test | 10.58 | 111.92 | 0.71 | 6.49 | 0.68 |
|  | Leave cells out | 11.91 | 141.94 | 0.60 | 7.91 | 0.56 |
|  | Leave drugs out | 14.05 | 197.35 | 0.34 | 9.37 | 0.33 |
|  | Leave drug combinations out | 11.36 | 128.94 | 0.52 | 8.31 | 0.60 |
| ETC *^b^* | Test | 10.50 | 110.29 | 0.71 | 6.76 | 0.68 |
|  | Leave cells out | 11.17 | 124.81 | 0.65 | 7.50 | 0.61 |
|  | Leave drugs out | 13.18 | 173.66 | 0.40 | 9.00 | 0.40 |
|  | Leave drug combinations out | 15.91 | 253.26 | 0.30 | 11.72 | 0.54 |
| KNN *^b^* | Test | 13.56 | 183.98 | 0.40 | 9.51 | 0.36 |
|  | Leave cells out | 13.17 | 173.53 | 0.44 | 9.31 | 0.41 |
|  | Leave drugs out | 13.90 | 193.08 | 0.28 | 9.89 | 0.30 |
|  | Leave drug combinations out | 16.12 | 259.90 | 0.11 | 12.49 | -0.05 |
| RF *^b^* | Test | 10.58 | 111.85 | 0.70 | 6.85 | 0.67 |
|  | Leave cells out | 11.20 | 125.44 | 0.65 | 7.55 | 0.60 |
|  | Leave drugs out | 13.34 | 178.05 | 0.38 | 9.06 | 0.37 |
|  | Leave drug combinations out | 15.67 | 245.62 | 0.36 | 11.60 | 0.56 |
| SVM *^b^* | Test | 15.07 | 227.07 | 0.22 | 10.04 | 0.20 |
|  | Leave cells out | 15.21 | 231.34 | 0.29 | 9.85 | 0.29 |
|  | Leave drugs out | 15.68 | 245.94 | 0.12 | 10.75 | 0.13 |
|  | Leave drug combinations out | 13.53 | 182.97 | 0.14 | 10.24 | 0.11 |
| XGB *^b^* | Test | 11.93 | 142.21 | 0.60 | 8.19 | 0.53 |
|  | Leave cells out | 11.83 | 139.88 | 0.60 | 8.22 | 0.53 |
|  | Leave drugs out | 13.66 | 186.60 | 0.32 | 9.59 | 0.29 |
|  | Leave drug combinations out | 17.62 | 310.60 | 0.08 | 13.95 | -0.11 |
| DL5 *^c^* | Test | 11.04 | 121.87 | 0.68 | 6.98 | 0.63 |
|  | Leave cells out | 12.39 | 153.48 | 0.55 | 8.31 | 0.50 |
|  | Leave drugs out | 14.72 | 216.77 | 0.32 | 10.11 | 0.30 |
|  | Leave drug combinations out | 11.19 | 125.21 | 0.50 | 8.30 | 0.56 |
| DL6 *^c^* | Test | 11.07 | 122.59 | 0.68 | 6.97 | 0.63 |
|  | Leave cells out | 12.53 | 157.07 | 0.54 | 8.44 | 0.48 |
|  | Leave drugs out | 14.96 | 223.78 | 0.29 | 10.22 | 0.29 |
|  | Leave drug combinations out | 11.70 | 136.85 | 0.50 | 8.53 | 0.57 |
| DL7 *^c^* | Test | 11.63 | 135.31 | 0.64 | 7.59 | 0.56 |
|  | Leave cells out | 13.58 | 184.32 | 0.50 | 9.16 | 0.46 |
|  | Leave drugs out | 15.73 | 247.53 | 0.24 | 10.84 | 0.20 |
|  | Leave drug combinations out | 11.31 | 127.96 | 0.46 | 8.70 | 0.44 |
| DL8 *^c^* | Test | 11.45 | 131.10 | 0.66 | 7.34 | 0.60 |
|  | Leave cells out | 12.95 | 167.67 | 0.51 | 8.76 | 0.46 |
|  | Leave drugs out | 14.93 | 223.03 | 0.32 | 10.21 | 0.28 |
|  | Leave drug combinations out | 11.61 | 134.85 | 0.43 | 8.45 | 0.44 |

*^a^* The metrics of the worst SGD-based model were not included in the table since they were out of range compared with the other DL and non-DL models.

*^b^* ML-based model architectures include: ETC (max_depth: None, min_samples_leaf: 4, min_samples_split: 2, n_estimators: 1000), KNN (n_neighbors: 25), RF (max_depth: None, min_samples_leaf: 2, min_samples_split: 2, n_estimators: 1000), SVM (C: 0.1), and XBG (alpha: 0.25, max_depth: 2, n_estimators: 1000).

*^c^* DL-based model architectures include: DL5 ([1000, 1000, 1000, 1000], 0.0, PCA_fillna), DL6 ([2114, 1057, 264, 16], 0.0, PCA_fillna), DL7 ([100, 100, 100], 0.0, PCA_fillna) and DL8 ([673, 336, 84], 0.0, PCA_fillna).

**Table 11.** Final metrics of the regression models evaluated in an independent test set and three different scenarios (leave cell out, leave drugs out and leave drug combinations out) using ZIP synergy reference model.

| Model *^a^* | Subset used for evaluation | RMSE | | MSE | Pearson | MAE | Spearman |
| --- | --- | --- | --- | --- | --- | --- | --- |
| ENS | Test | | 3.86 | 14.87 | 0.70 | 2.74 | 0.66 |
|  | Leave cells out | | 4.72 | 22.30 | 0.55 | 3.51 | 0.52 |
|  | Leave drugs out | | 5.21 | 27.16 | 0.51 | 3.80 | 0.48 |
|  | Leave drug combinations out | | 7.78 | 60.48 | 0.01 | 5.14 | 0.09 |
| ETC *^b^* | Test | | 3.91 | 15.29 | 0.68 | 2.78 | 0.65 |
|  | Leave cells out | | 4.59 | 21.09 | 0.57 | 3.41 | 0.54 |
|  | Leave drugs out | | 4.85 | 23.55 | 0.58 | 3.58 | 0.52 |
|  | Leave drug combinations out | | 7.31 | 53.45 | -0.04 | 4.78 | 0.09 |
| KNN *^b^* | Test | | 4.72 | 22.26 | 0.46 | 3.35 | 0.45 |
|  | Leave cells out | | 5.08 | 25.78 | 0.43 | 3.76 | 0.40 |
|  | Leave drugs out | | 5.31 | 28.17 | 0.42 | 3.87 | 0.38 |
|  | Leave drug combinations out | | 7.06 | 49.79 | 0.08 | 4.73 | 0.05 |
| RF *^b^* | Test | | 3.97 | 15.79 | 0.67 | 2.83 | 0.64 |
|  | Leave cells out | | 4.62 | 21.30 | 0.57 | 3.43 | 0.53 |
|  | Leave drugs out | | 4.93 | 24.34 | 0.56 | 3.65 | 0.49 |
|  | Leave drug combinations out | | 6.70 | 44.88 | 0.14 | 4.53 | 0.17 |
| SVM *^b^* | Test | | 5.72 | 32.75 | 0.24 | 4.25 | 0.25 |
|  | Leave cells out | | 5.16 | 26.58 | 0.40 | 3.74 | 0.42 |
|  | Leave drugs out | | 6.68 | 44.65 | 0.17 | 5.09 | 0.16 |
|  | Leave drug combinations out | | 8.02 | 64.26 | -0.07 | 5.61 | 0.00 |
| XGB *^b^* | Test | | 4.68 | 21.92 | 0.48 | 3.36 | 0.47 |
|  | Leave cells out | | 4.91 | 24.08 | 0.50 | 3.63 | 0.48 |
|  | Leave drugs out | | 5.28 | 27.89 | 0.45 | 3.90 | 0.41 |
|  | Leave drug combinations out | | 7.24 | 52.43 | -0.13 | 4.87 | -0.00 |
| DL18*^c^* | Test | | 4.20 | 17.67 | 0.63 | 3.00 | 0.60 |
|  | Leave cells out | | 5.00 | 25.01 | 0.46 | 3.72 | 0.43 |
|  | Leave drugs out | | 5.60 | 31.40 | 0.44 | 4.11 | 0.39 |
|  | Leave drug combinations out | | 7.56 | 57.19 | 0.11 | 5.32 | 0.10 |
| DL19*^c^* | Test | | 4.20 | 17.64 | 0.64 | 2.98 | 0.60 |
|  | Leave cells out | | 5.01 | 25.13 | 0.46 | 3.73 | 0.44 |
|  | Leave drugs out | | 5.61 | 31.44 | 0.45 | 4.10 | 0.41 |
|  | Leave drug combinations out | | 6.71 | 45.06 | 0.22 | 4.66 | 0.22 |
| DL20 *^c^* | Test | | 4.37 | 19.10 | 0.59 | 3.12 | 0.57 |
|  | Leave cells out | | 5.43 | 29.46 | 0.38 | 4.05 | 0.3 |
|  | Leave drugs out | | 5.97 | 35.61 | 0.40 | 4.31 | 0.37 |
|  | Leave drug combinations out | | 7.27 | 52.89 | 0.12 | 5.28 | 0.08 |

*^a^* The metrics of the worst SGD-based model were not included in the table since they were out of range compared with the other DL and non-DL models.

*^b^* ML-based model architectures include: ETC (max_depth: None, min_samples_leaf: 2, min_samples_split: 10, n_estimators: 1000), KNN (n_neighbors: 25), RF (max_depth: None, min_samples_leaf: 2, min_samples_split: 5, n_estimators: 1000), SVM (C: 0.1), and XGB (alpha: 0.5, max_depth: 2, n_estimators: 100).

*^c^* DL-based model architectures include: DL18 ([500, 500, 500, 500], 0.0, PCA_fillna), DL19 ([2500, 2500, 2500], 0.0, PCA_fillna) and DL20 ([100, 100, 100], 0.0, PCA_fillna).

**Table 12.** Final metrics of the regression models evaluated in an independent test set and three different scenarios (leave cell out, leave drugs out and leave drug combinations out) using CSS.

| Model *^a^* | Subset used for evaluation | RMSE | | MSE | Pearson | MAE | Spearman |
| --- | --- | --- | --- | --- | --- | --- | --- |
| ENS | Test | | 11.07 | 122.61 | 0.86 | 7.43 | 0.87 |
|  | Leave cells out | | 13.63 | 185.70 | 0.74 | 10.34 | 0.75 |
|  | Leave drugs out | | 17.72 | 313.98 | 0.60 | 13.01 | 0.58 |
|  | Leave drug combinations out | | 22.63 | 512.32 | 0.46 | 15.93 | 0.43 |
| ETC *^b^* | Test | | 10.97 | 120.32 | 0.86 | 7.79 | 0.87 |
|  | Leave cells out | | 12.34 | 152.16 | 0.79 | 9.47 | 0.79 |
|  | Leave drugs out | | 16.18 | 261.71 | 0.65 | 12.09 | 0.62 |
|  | Leave drug combinations out | | 15.89 | 252.46 | 0.79 | 12.47 | 0.72 |
| KNN *^b^* | Test | | 16.45 | 270.54 | 0.66 | 12.47 | 0.64 |
|  | Leave cells out | | 13.79 | 190.09 | 0.73 | 10.53 | 0.74 |
|  | Leave drugs out | | 17.04 | 290.35 | 0.60 | 12.77 | 0.57 |
|  | Leave drug combinations out | | 27.93 | 779.93 | 0.54 | 23.66 | 0.49 |
| RF *^b^* | Test | | 11.68 | 136.45 | 0.84 | 8.37 | 0.85 |
|  | Leave cells out | | 12.80 | 163.80 | 0.77 | 9.85 | 0.77 |
|  | Leave drugs out | | 16.68 | 278.09 | 0.64 | 12.44 | 0.60 |
|  | Leave drug combinations out | | 15.39 | 236.76 | 0.80 | 12.43 | 0.72 |
| SVM *^b^* | Test | | 16.10 | 259.23 | 0.68 | 12.29 | 0.69 |
|  | Leave cells out | | 15.37 | 236.08 | 0.65 | 11.91 | 0.65 |
|  | Leave drugs out | | 19.43 | 377.55 | 0.44 | 15.30 | 0.41 |
|  | Leave drug combinations out | | 16.64 | 276.88 | 0.73 | 13.16 | 0.63 |
| XGB *^b^* | Test | | 13.02 | 169.51 | 0.80 | 9.93 | 0.81 |
|  | Leave cells out | | 13.34 | 178.04 | 0.74 | 10.40 | 0.76 |
|  | Leave drugs out | | 16.62 | 276.21 | 0.62 | 12.75 | 0.60 |
|  | Leave drug combinations out | | 15.42 | 237.79 | 0.78 | 12.47 | 0.69 |
| DL21*^c^* | Test | | 11.91 | 141.74 | 0.84 | 8.15 | 0.85 |
|  | Leave cells out | | 14.32 | 205.01 | 0.71 | 10.83 | 0.73 |
|  | Leave drugs out | | 18.59 | 345.74 | 0.57 | 13.70 | 0.55 |
|  | Leave drug combinations out | | 26.62 | 708.38 | 0.32 | 17.43 | 0.35 |
| DL22*^c^* | Test | | 11.85 | 140.45 | 0.84 | 8.01 | 0.85 |
|  | Leave cells out | | 14.72 | 216.79 | 0.70 | 11.12 | 0.72 |
|  | Leave drugs out | | 18.55 | 343.96 | 0.56 | 13.67 | 0.55 |
|  | Leave drug combinations out | | 22.96 | 527.08 | 0.46 | 16.33 | 0.43 |
| DL23*^c^* | Test | | 11.88 | 141.19 | 0.84 | 8.04 | 0.85 |
|  | Leave cells out | | 14.14 | 199.84 | 0.72 | 10.70 | 0.74 |
|  | Leave drugs out | | 18.29 | 334.35 | 0.58 | 13.42 | 0.56 |
|  | Leave drug combinations out | | 24.16 | 583.67 | 0.39 | 17.08 | 0.38 |
| DL24*^c^* | Test | | 12.19 | 148.60 | 0.83 | 8.59 | 0.84 |
|  | Leave cells out | | 14.18 | 201.07 | 0.71 | 10.89 | 0.72 |
|  | Leave drugs out | | 18.22 | 331.99 | 0.56 | 13.74 | 0.53 |
|  | Leave drug combinations out | | 21.80 | 475.35 | 0.53 | 15.43 | 0.44 |

*^a^* The metrics of the worst SGD-based model were not included in the table since they were out of range compared with the other DL and non-DL models.

*^b^* ML-based model architectures include: ETC (max_depth: None, min_samples_leaf: 1, min_samples_split: 10, n_estimators: 1000), KNN (n_neighbors: 5), RF (max_depth: None, min_samples_leaf: 2, min_samples_split: 2, n_estimators: 1000), SVM (C: 0.1), and XGB (alpha: 0, max_depth: 2, n_estimators: 1000).

*^c^* DL-based model architectures include: DL21 ([2500, 2500, 2500], 0.0, PCA_fillna), DL22 ([2500, 2500, 2500, 2500], 0.0, PCA_fillna), DL23 ([2114, 1057, 264, 16], 0.0, PCA_fillna) and DL24 ([100, 100, 100, 100], 0.0, PCA_fillna).

**Table 13.** DeepSynergy [1] reimplementation on the dataset that yielded the best results for SynPred (with PCA preprocessing and missing values replacement with 0), against the synergy reference model the original work targeted - Loewe.

|  | RMSE | MSE | Pearson | Spearman |
| --- | --- | --- | --- | --- |
| *Test* | 11.31 | 128.00 | 0.65 | 0.60 |
| *Leave cells out* | 11.70 | 136.89 | 0.61 | 0.55 |
| *Leave drugs out* | 13.88 | 192.66 | 0.32 | 0.30 |
| *Leave drug combinations out* | 11.85 | 140.39 | 0.42 | 0.41 |

**Table 14.** Comparison of final metrics of the classification and regression models of SynPred to the methods reviewed by Kumar et al [2].

| *Classification* | | | | |
| --- | --- | --- | --- | --- |
|  | *Accuracy* | *Recall* | *AUROC* | *Precision* |
| *Average* | *0.79* | *0.60* | *0.85* | *0.73* |
| *Best* | *0.93* | *0.79* | *0.96* | *0.83* |
| *SynPred* | *0.85* | *0.90* | *0.80* | *0.91* |
| *Regression* | | | | |
|  | *RMSE* | *MSE* | *Pearson* | *Spearman* |
| *Average* | *15.69* | *207.95* | *0.80* | *0.75* |
| *Best* | *15.48* | *131.90* | *0.83** | *0.81* |
| *SynPred - CSS* | 11.07 | 122.61 | 0.86 | 0.87 |
| *SynPred - Loewe* | 10.58 | 111.92 | 0.71 | 0.68 |
| *SynPred - Bliss* | 4.35 | 18.92 | 0.71 | 0.59 |
| *SynPred - HSA* | 4.09 | 16.70 | 0.73 | 0.64 |
| *SynPred - ZIP* | 3.86 | 14.87 | 0.70 | 0.66 |

*Simple ML low performance methods were excluded from average and maximum calculations. *0.95, the highest Pearson performance attributed in Kumar et al* [2]*., was taken out as further inspection of Sun et al. 2020* [3] *(DTF prediction algorithm), revelated that this value referred to the comparison between two prediction algorithms, and not between prediction values and real values. As such, the second reported best performing method* [4] *was considered.*

**Table 15.** Comparison of the performance of SynPred and other recent algorithms, according to their respective reporting metrics upon deployment in DrugCombo [5].

| DRUGCOMBO | RMSE | MSE | Pearson | Spearman |
| --- | --- | --- | --- | --- |
| *SynPred - CSS* | 15.05 | 226.45 | 0.73 | 0.75 |
| *SynPred - Loewe* | 11.11 | 123.38 | 0.67 | 0.63 |
| *SynPred - Bliss* | 11.70 | 136.80 | 0.33 | 0.54 |
| *SynPred - HSA* | 7.45 | 55.54 | 0.48 | 0.58 |
| *SynPred - ZIP* | 6.74 | 45.41 | 0.48 | 0.59 |
| *Matchmakers’** [6] | - | 74.49 | 0.79 | 0.74 |
| *Deepsynergy** [1] | - | 105.90 | ≈ 0.70 | ≈ 0.65 |
| *Treecombo** [7] | - | 13.70 | ≈ 0.60 | ≈ 0.55 |
| *RF** | - | 173.2 | ≈ 0.43 | ≈ 0.33 |
| *ElasticNET** | - | 183.4 | ≈ 0.37 | ≈ 0.31 |

** performance values retrieved from Kuru et al. [6]; ≈ as these values were only provided in Figure format by the original authors [6].*

**Table 16.** Comparison of the performance of SynPred and other recent algorithms, according to their respective reporting metrics upon deployment in NCI ALMANAC [8].

| **NCI ALMANAC** | **RMSE** | **MSE** | **Pearson** | **Spearman** |
| --- | --- | --- | --- | --- |
| ***SynPred - CSS*** | 8.35 | 68.68 | 0.77 | 0.75 |
| ***SynPred - Loewe*** | 5.80 | 33.69 | 0.84 | 0.82 |
| ***SynPred - Bliss*** | 3.13 | 9.77 | 0.83 | 0.83 |
| ***SynPred - HSA*** | 3.00 | 8.98 | 0.79 | 0.78 |
| ***SynPred - ZIP*** | 2.69 | 7.23 | 0.84 | 0.86 |
| ***Matchmakers’ [6]*** | - | 3014.5 | 0.59 | 0.55 |
| ***Deepsynergy*** [1] | - | 3881.6 | 0.40 | 0.36 |
| ***Treecombo*** [7] | - | 3651.3 | 0.47 | 0.47 |
| ***RF*** | - | 3932.2 | 0.38 | 0.39 |
| ***ElasticNET*** | - | 4176.5 | 0.30 | 0.33 |

**REFERENCES**

1. Preuer K, Lewis RPI, Hochreiter S, Bender A, Bulusu KC, Klambauer G. DeepSynergy: predicting anti-cancer drug synergy with Deep Learning. Wren J, editor. *Bioinformatics*. 2018; doi: 10.1093/bioinformatics/btx806.

2. Kumar V, Dogra N. A Comprehensive Review on Deep Synergistic Drug Prediction Techniques for Cancer. *Archives of Computational Methods in Engineering*. 2022; doi: 10.1007/s11831-021-09617-3.

3. Sun Z, Huang S, Jiang P, Hu P. DTF: Deep Tensor Factorization for predicting anticancer drug synergy. *Bioinformatics*. 2020; doi: 10.1093/bioinformatics/btaa287.

4. Wang J, Liu X, Shen S, Deng L, Liu H. DeepDDS: deep graph neural network with attention mechanism to predict synergistic drug combinations. *Briefings in Bioinformatics*. 2021; doi: 10.1093/bib/bbab390.

5. Zagidullin B, Aldahdooh J, Zheng S, Wang W, Wang Y, Saad J, et al.. DrugComb: an integrative cancer drug combination data portal. *Nucleic Acids Res*. 2019; doi: 10.1093/nar/gkz337.

6. Kuru HI, Tastan O, Cicek AE. Matchmakers’: A Deep Learning Framework for Drug Synergy Prediction. *IEEE/ACM Trans Comput Biol Bioinform*. 2021; doi: 10.1109/TCBB.2021.3086702.

7. Janizek JD, Celik S, Lee S-I. Explainable machine learning prediction of synergistic drug combinations for precision cancer medicine. 2018; doi: 10.1101/331769.

8. Holbeck SL, Camalier R, Crowell JA, Govindharajulu JP, Hollingshead M, Anderson LW, et al.. The National Cancer Institute ALMANAC: A Comprehensive Screening Resource for the Detection of Anticancer Drug Pairs with Enhanced Therapeutic Activity. *Cancer Res*. 2017; doi: 10.1158/0008-5472.CAN-17-0489.
