# Supplementary material for: SYNPRED: prediction of drug combination effects in cancer using different synergy metrics and ensemble learning
Source: Gigascience. 2022 Sep 26;11:giac087. doi: 10.1093/gigascience/giac087 (PMC9511701; doi:10.1093/gigascience/giac087)
Supplement: giac087_Response_to_Reviewer_Comments_Original_Submission [file giac087_response_to_reviewer_comments_original_submission.pdf]

# Author's Response To Reviewer Comments

Close

Reviewer reports:
Reviewer #1: I thank the authors for improving the performance assessment of the method. They now include the regression results and different experimental setups for splitting the dataset and have taken measures to prevent information leakage. The manuscript is much stronger compared to initial version. However, I still have some concerns indicated below.

Major issues:
1 - Authors indicate that they did not use ComboScore because "… it was not clear from the literature how the synergy scores of different drug concentrations could be aggregated." However, NCI-60 study itself provides ComboScores and they give all the details about the procedure. As is, the statement in the paper claims no documentation exists but it does. Please clarify this.
In our newest manuscript version, we have retrained all the AI models with the synergy reference models computed by the top accepted compound synergy database existent in the literature: DrugComb [1]– ZIP, Bliss, HSA, Bliss and Combination Sensitivity Score (CSS). The main addition here regarding previously submitted manuscript version was the use of CSS [2], which is fully documented. We are confident that this step is a further improvement as we have now assessed the effect of using five different metrics on the final models' performance.
The decision of not using ComboScore, available only for NCI ALMANAC is supported by the fact that this metric would limit the easy application of our method to this specific dataset. Moreover, as DrugComb's authors state: "However, we recommend that only if a drug combination that achieves a higher synergy score in all the models (i.e. S, BLISS, HSA, LOEWE, ZIP) as well as a higher sensitivity score (CSS) should be prioritized for deeper validations." [1]

2 - In the previous revision cycle I had asked the authors to compare their method with established methods from the literature such as DeepSynergy, AuDNNSynerygy and Matchmaker. However, there is no comparison presented.
While the authors claim that "Many of the works do not make available the predictors so that it is possible to redeploy them adequately". These have publicly available implementations. They indicate that the pipeline might not be fully available for preprocessing. In this case they need to contact the authors of the studies for clarification.
They need to either feed SynPred's preprocessed data to these models, or feed the data used by other methods to SynPred. Authors also indicate that "OMICs data is not available for all pairs." This is actually a shortcoming which prohibits increasing the complexity of their architecture. I would like to see a comparison with methods that use smaller number of features but more complex architectures so that a user can decide which method to use.
The authors can even easily modify these architectures to work with their synergy scores of interest and have them to work in classification or regression settings. The authors claim good performance, but is it better than the state of the art methods? If not, why should we use this method over others? What are other novel contributions?

Benchmarking of synergy prediction protocols is a complicated process. As highlighted by Zagidullin et al. [1], the datasets available completely differ in the amount of information used, with DrugComb [1] assembling the most important ones (ALMANAC [3], ONEIL [4], FORCINA [5], CLOUD [6]). As showed by Kumar et al. [7], majority of authors used NCI-ALMANAC [3] to train and the concept of Loewe additivity model [8–13]. Furthermore, comparison to the available methodologies implies that authors adapt the published proposed DL architectures as these are not easily applied or not available in GitHub or similar platforms. As such, we followed a multi-step approach to benchmark our pipeline:
i) Comparison of the best performing individual DL and ML algorithms with the ensemble approaches for each prediction task- Tables 7 to 12_Supplementary Material.
ii) DeepSynergy [18] architecture implementation and comparison using our independent test set and validation sets - Table 14_Supplementary Material.
iii) Comparison with published methods for synergy calculations as reviewed by Kumar et al. [7] - Table 13_Supplementary Material.

iv) Comparison of our regression approaches to MatchMaker algorithm [20], DeepSynergy [18] and TreeCombo [12] - Tables 15 and 16_Supplementary Material.
Regarding i), ensemble/aggregation of algorithms consistently outperform, or stand very close to the best individual predictors. XGBoost and Extreme Randomized Trees were typically the second-best predictors. This showcases how SynPred leverages previous information on algorithms such as TreeCombo [12] (uses an individual XGBoost algorithm) or DeepSynergy [18], which is, in essence, the literature parent of several of the neural networks with conic architecture we used. In fact, in ii), (supplementary table 13) it can be seen that the DeepSynergy [18] implementation on SynPred's pipeline behaves similarly to other DNN approaches in SynPred. These are good performers, but unable to beat the ensemble algorithms.

When comparing the reported performance for algorithms in their own settings iii), as reviewed by Kumar et al. [65], once again we need to take into account a very broad array of circumstances, such as algorithms, datasets, and synergy reference models. For instance, SynPred highest performer predictor is clearly the CSS predictor. However, it is not possible to justly compare our result to predictors that only focus on the Loewe synergy reference model. On the other hand, when considering the most recurring synergy reference model (Loewe), albeit SynPred shows lower Pearson and Spearman correlations, it also shows much lower errors (RMSE and MSE) in comparison to the best remaining algorithms. All this serves to highlight the need to account for different synergy reference models, which had not been previously achieved, but already suggested to be a valuable approach [29].

Finally (iv), we performed closer comparisons (although still not optimal) in supplementary tables 14 and 15. These more recent applications use more readily available datasets. Regarding table 14, SynPred was ran against Matchmakers' [20] processing of DrugComb [29]. Upon doing this, both CSS and Loewe predictors from SynPred stood very close to the performance of Matchmaker [20], which is remarkable, since this was the dataset used by the authors [20] to train the dataset. When inspecting supplementary table 15, in which the predictors were deployed upon NCI-ALMANAC [85], the dataset in which SynPred focuses, SynPred clearly stands out in all the synergy reference models with Pearson and Spearman correlations performance increasements between 30.51% and 42.37%, and between 36.36% and 56.36%, respectively. MSE also saw significant improvements.

3- I did not understand whether authors used the complete ALMANAC dataset or just the samples that satisfy their "full-agreement" property. If they used only the fully-agreed samples, the results may be overoptimistic since these samples are probably easy examples to predict. All other models in the literature stated above use the complete dataset of interest and they do not carefully select the test examples. Authors are free to cherry pick examples for training, but for a fair comparison with others, they have to sample from the whole ALMANAC dataset for testing.
We have now used two approaches for synergy prediction:
i) classification, for which we used a full-agreement methodology, based on the use of more meaningful consensus data, and minimizing the inherent data noise. It is particularly relevant such conservative approach if we have into account that only 13% drug combination metrics are in full agreement at the portion of the state-of-the-art NCI-ALMANAC [3] dataset used to train SynPred (195.996 combinations that, upon full agreement processing, yielded 20.291 synergistic and 6.419 non-synergistic samples).
ii) regression, for which all 195.996 samples were for to train the respective models for regression tasks. As mentioned in question 2, we have also used the dataset available by other authors, particularly relevant to the various regression-based SynPred models, as input for our methodology. All the different benchmarking experiences were detailed in the revised manuscript as well in our previous answer.
To better clarify this point we further modified lines 252-272 of the revised manuscript. We hope that now the choice and use of dataset as well as the attained performance and its benchmarking is better clarified and that no doubts remain regarding data cherry-picking.

Minor issues:
1- For the different split schemes, please clarify if the training and validation sets are identical for all models which participate in the ensemble.
The training and validation splits were performed beforehand, using only the identifiers, as such, they are the same for all the models referred throughout the manuscript (see lines 252-272). This sentence "The described data splitting was performed prior to any model training, thus ensuring all the prediction models' performance evaluation is deployed on the same data" (lines 270-272) was added, and we hope any doubt regarding the subject was now clarified.

2 - Please name the "test" dataset in the Table 2. It is not clear what is the difference between this test set and the analysis performed on DECREASE dataset mentioned in lines 439 - 447.

This test set is a portion of the dataset randomly retrieved from the full table. Considering the new splits (leave cells out, leave drugs out and leave drug combinations out), this test set was only retrieved after the new splits had been performed, thus ensuring the validity of the model, this has been rephrased on lines 252-272.

3- Figure 2, please add y axis label.
Following reviewers ‘suggestion, we have now added, the y axis. Furthermore, we also added the new used metric, CSS, to the plot.

4 - Please rephrase the following sentence: “We considered outliers, the synergy prediction values above or below 10 times the average of the remaining prediction values.”
Rephrased and extended the explanation to “For some tasks, when deploying the individual predictors, a few of these had notably bad performance (mostly SGD and KNN), as such we considered outliers the synergy prediction values above or below 10 times the average of the remaining prediction values; this was necessary to allow the ensemble neural networks to converge.” (lines 312-315)

Reviewer #2: The paper uses multiple synergy metrics to compare DL and non-DL state-of-the-art approaches to predict drug synergy combinations and provides a web interface for researchers to explore synergistic drug combinations for the submitted drug combinations.
The authors addressed the main points previously raised by me and the other reviewer namely;
* adding regression analysis for synergy prediction
* using different train test splitting strategies
* comparison by running reference models
* validation with an independent dataset
I have no more concerns.

We thank the reviewer for his comments.

REFERENCES

1. Zagidullin B, Aldahdooh J, Zheng S, Wang W, Wang Y, Saad J, et al.. DrugComb: an integrative cancer drug combination data portal. Nucleic Acids Res. 2019; doi: 10.1093/nar/gkz337.
2. Malyutina A, Majumder MM, Wang W, Pessia A, Heckman CA, Tang J. Drug combination sensitivity scoring facilitates the discovery of synergistic and efficacious drug combinations in cancer. PLoS Comput Biol. 2019; doi: 10.1371/journal.pcbi.1006752.
3. Holbeck SL, Camalier R, Crowell JA, Govindharajulu JP, Hollingshead M, Anderson LW, et al.. The National Cancer Institute ALMANAC: A Comprehensive Screening Resource for the Detection of Anticancer Drug Pairs with Enhanced Therapeutic Activity. Cancer Res. 2017; doi: 10.1158/0008-5472.CAN-17-0489.
4. O’Neil J, Benita Y, Feldman I, Chenard M, Roberts B, Liu Y, et al.. An Unbiased Oncology Compound Screen to Identify Novel Combination Strategies. Mol Cancer Ther. 2016; doi: 10.1158/1535-7163.MCT-15-0843.
5. Forcina GC, Conlon M, Wells A, Cao JY, Dixon SJ. Systematic Quantification of Population Cell Death Kinetics in Mammalian Cells. Cell Systems. 2017; doi: 10.1016/j.cels.2017.05.002.
6. Licciardello MP, Ringler A, Markt P, Klepsch F, Lardeau C-H, Sdelci S, et al.. A combinatorial screen of the CLOUD uncovers a synergy targeting the androgen receptor. Nat Chem Biol. United States; 2017; doi: 10.1038/nchembio.2382.
7. Kumar V, Dogra N. A Comprehensive Review on Deep Synergistic Drug Prediction Techniques for Cancer. Archives of Computational Methods in Engineering. 2022; doi: 10.1007/s11831-021-09617-3.
8. Celebi R, Bear Don’t Walk O, Movva R, Alpsoy S, Dumontier M. In-silico Prediction of Synergistic Anti-Cancer Drug Combinations Using Multi-omics Data. Sci Rep. 2019; doi: 10.1038/s41598-019-45236-6.
9. Zhang T, Zhang L, Payne PRO, Li F. Synergistic Drug Combination Prediction by Integrating Multiomics Data in Deep Learning Models. In: Markowitz J, editor. Translational Bioinformatics for Therapeutic Development. New York, NY: Springer US;
10. Wang J, Liu X, Shen S, Deng L, Liu H. DeepDDS: deep graph neural network with attention mechanism to predict synergistic drug combinations. Briefings in Bioinformatics. 2021; doi: 10.1093/bib/bbab390.
11. Preuer K, Lewis RPI, Hochreiter S, Bender A, Bulusu KC, Klambauer G. DeepSynergy: Predicting anti-cancer drug synergy with Deep Learning. Bioinformatics. 2018; doi: 10.1093/bioinformatics/btx806.
12. Kuru HI, Tastan O, Cicek AE. MatchMaker: A Deep Learning Framework for Drug Synergy Prediction. IEEE/ACM Trans Comput Biol Bioinform. 2021; doi: 10.1109/TCBB.2021.3086702.

13. Liu Q, Xie L. TranSynergy: Mechanism-driven interpretable deep neural network for the synergistic prediction and pathway deconvolution of drug combinations. Schlessinger A, editor. PLoS Comput Biol. 2021; doi: 10.1371/journal.pcbi.1008653.

Close
