# Supplementary material for: SYNPRED: prediction of drug combination effects in cancer using different synergy metrics and ensemble learning
Source: Gigascience. 2022 Sep 26;11:giac087. doi: 10.1093/gigascience/giac087 (PMC9511701; doi:10.1093/gigascience/giac087)
Supplement: giac087_Response_to_Reviewer_Comments_Revision_1 [file giac087_response_to_reviewer_comments_revision_1.pdf]

Author's Response To Reviewer Comments

Close

Coimbra, 2h August 2022
RE: Manuscript ID No.: GIGA-D-21-00121

Dear Prof. Hans Zauner,
Thank you for handling our manuscript throughout this process.
All changes to the manuscript are marked in red. The answers to the reviewer's specific comments are included below. We hope that our manuscript SYNPRED: Prediction of Drug Combination Effects in Cancer using Different Synergy Metrics and Ensemble Learning" fulfils now the necessary quality criteria to be published at GigaScience.
Yours sincerely,
Irina S. Moreira, Ph.D.

Reviewer #1: Authors indicate that benchmarking of synergy prediction protocols is a complicated process. While I agree with this. , I do not agree that "..DL architectures as these are not easily applied or not available in GitHub or similar platforms." As the implementations are available on GitHub, the authors can feed their data of choice to easily train these models. There might be some adaptation choices to make such as pruning the data due to unavailability of a certain data modality, or changing the loss function to turn a model into a regressor, but as long as they justify these it should be fine and east. This is what has been done in the literature in DeepSynergy and Matchmaker papers, for instance. They have done this to a degree but I am not convinced that a fair comparison with the literature I made to conclude that this method has clear advantages over others. Authors indicate that they trained 1972 models but I think these are mostly the "baseline" models they use within their ensemble method which does not help in comparing with the literature. Below, I list my detailed comments on the revisions made:
First, while there are many detailed descriptive statistics such as distribution of synergy scores are presented in the main text the comparisons are pushed to the supplement which makes it hard to follow the paper. Also, details on whether or how compared methods are run/trained is missing. For instance in comparison ii), how did you reimplement DeepSynergy, and in Comparison ii) and iv) what data did you use and what hyperparameters did you use to run DeepSynergy and Matchmaker? In the current revision, in Tables S7 - S12 authors compare their method with some baseline models used in their ensemble such as random forest, KNN and some custom deep learning architectures. I did not ask for this as clearly the problem is complex and a complex model as their method is needed. Nevertheless this is a nice validation which improves the paper. They compare their method with a reimplemented (please clarify in text) version of DeepSynergy in Table S13 (They say Table S14 but I think it is a typo).
We have corrected the typo and changed it to Table 13_Supplementary Material.
As thoroughly discussed, there is a fundamental disagreement between the authors' and the reviewer on this matter. A correct benchmark on this problem is not currently feasible, as we have attempted to make it clear to the reviewer. The benchmarking problem is evidenced by the publication of ChemicalX (Rozemberczki et al. 2022), in which DeepSynergy, MatchMaker, among others algorithm are re-implemented. This work was published in 2022 by several researchers. The fact that they had to re-implement the algorithms shows what we have stated for several versions of the answers to the reviewer: the present state of the art does not allow for proper benchmark. Unfortunately, ChemicalX only provides a binary class for drug synergy prediction.
However, we deployed a multi-step approach that compares individually either datasets, Machine Learning deployments or literature referenced results. We indeed benchmarked our pipeline as described in the methods section:
i) Comparison of the best performing individual DL and ML algorithms with the ensemble approaches for each prediction task - Tables 7 to 12_Supplementary Material.
ii) DeepSynergy architecture implementation and comparison using our independent test set and validation sets - Table 13_Supplementary Material.
iii) Comparison with published methods for synergy calculations as reviewed by Kumar et al. - Table 14_Supplementary Material.
iv) Comparison of our regression approaches to Matchmakers' algorithm, DeepSynergy and TreeCombo -

Tables 15 and 16_Supplementary Material.

First, I strongly suggest they put the result of the final method into this table (and other related ones) for the readability. I had to trace Table 4/5 to see their results and come back to compare with the values in this table. They report better results than DeepSynergy which is good. As they use the review of Kumar et al. later, there are other methods reported in this review which perform better than DeepSynergy in the regression task such as MultitaskDNN, AUDNNSynergy, and Matchmaker. However only DeepSynergy is compared. Later they perform a comparison with Matchmaker but it is ot clear to me why they had to perform a separate comparison instead of comparing them all. In supplementary table 14, they compare the performance of their method, with performance values of others reported in a review by Kumar et al. This is not meaningful at all as every method use different settings as authors also indicate. Once again, they have to retrain these (or similar) architectures with their (maybe filtered or postprocessed) data and compare. Just like done for DeepSynergy only in the regression task. They can as well retrain their method with the dataset of these methods as another point of view. For the results in Tables S15 - S16 (again the table numbers are wrong), author state "... Upon doing this, both CSS and Loewe predictors from SynPred stood very close to the performance of Matchmaker [20],". Matchmaker reports Loewe scores so if no retraining of the model is performed with CSS it is not meaningful to compare these results. I suggest authors to use the CSS scores as the label in training Matchmaker. In Loewe scores their performance actually does not stand vey close to Matchmaker (MSE vs 123, Pearson .79 vs .73). Only in Spearman correlation their result is 0.01 better. In Table S16, SynPred seems to be 50 times better than all others in terms of MSE. However, while SynPred reports various scores other than ComboScore, the results obtained for other methods are based on ComboScore. Thus, it is comparing apples and oranges and is not meaningful. Again, no training is performed in this comparison. Please do not put results with different metrics into the same table as it is very confusing for the reader.
The complexity of the benchmark problem is thoroughly addressed throughout the manuscript, and it cannot be reduced to a method against method comparison.
We have reimplemented DeepSynergy the same way the other authors that claim do benchmark DeepSynergy did, by reimplementing a neural network with the same dimensions and hyperparameters although on other data. We also compared the results of our predictor on Matchmakers' dataset and the results attained by these authors. The clarifications regarding implementation are now in page 16 of the revised manuscript.
Table S16 displays several predictors, many of which do not have ComboScore as target. We clearly state which SynPred predictors we are referring to by adding the name of target variable in front of SynPred.
We did expand our comments MSE, precisely because we are aware that error derived metrics are particularly hard to compare between datasets and methods. Clarified in page 26 of the revised manuscript.
We do not believe that adding table 4 and 5 in some of supplementary tables will increase readability, just the opposite and we did not follow this suggestion.
We have changed the numbering of tables as indeed these were incorrect.

The details on the dataset used, the train/validation/test splits and number of samples etc. are missing so it is hard to evaluate these scores.
This statement is purely wrong, all requested data is available on Table 3_Supplementary Material and detailed in text (subsection "Model evaluation and performance metrics", lines 252-276).

TYPOs: NCI ALMANAC, Matchmakers'
We have followed reviewer advice and changed the two types: NCI-ALMANC towards NCI ALMANAC and Matchmaker to Matchmakers' in the main document as well as in SI.

Close
