# Supplementary material for: SYNPRED: prediction of drug combination effects in cancer using different synergy metrics and ensemble learning
Source: Gigascience. 2022 Sep 26;11:giac087. doi: 10.1093/gigascience/giac087 (PMC9511701; doi:10.1093/gigascience/giac087)
Supplement: giac087_Reviewer_1_Report_Original_Submission — A. ErcÃ¼ment Ã‡iÃ§ek -- 1/16/2022 Reviewed [file giac087_reviewer_1_report_original_submission.pdf]

Reviewer Report

Title: SYNPRED: Prediction of Drug Combination Effects in Cancer using Different Synergy Metrics and Ensemble Learning

Version: Original Submission **Date:** 1/16/2022

Reviewer name: A. ErcÃ¼ment Ã‡iÃ§ek

Reviewer Comments to Author:

I thank the authors for improving the performance assessment of the method. They now include the regression results and different experimental setups for splitting the dataset and have taken measures to prevent information leakage. The manuscript is much stronger compared to initial version. However, I still have some concerns indicated below.

Major issues:

1 - Authors indicate that they did not use ComboScore because "… it was not clear from the literature how the synergy scores of different drug concentrations could be aggregated." However, NCI-60 study itself provides ComboScores and they give all the details about the procedure. As is, the statement in the paper claims no documentation exists but it does. Please clarify this.

2 - In the previous revision cycle I had asked the authors to compare their method with established methods from the literature such as DeepSynergy, AuDNNSynerygy and Matchmaker. However, there is no comparison presented.

While the authors claim that "Many of the works do not make available the predictors so that it is possible to redeploy them adequately". These have publicly available implementations. They indicate that the pipeline might not be fully available for preprocessing. In this case they need to contact the authors of the studies for clarification.

They need to either feed SynPred's preprocessed data to these models, or feed the data used by other methods to SynPred. Authors also indicate that "OMICs data is not available for all pairs." This is actually a shortcoming which prohibits increasing the complexity of their architecture. I would like to see a comparison with methods that use smaller number of features but more complex architectures so that a user can decide which method to use.

The authors can even easily modify these architectures to work with their synergy scores of interest and have them to work in classification or regression settings. The authors claim good performance, but is it better than the state of the art methods? If not, why should we use this method over others? What are other novel contributions?

3- I did not understand whether authors used the complete ALMANAC dataset or just the samples that satisfy their "full-agreement" property. If they used only the fully-agreed samples, the results may be overoptimistic since these samples are probably easy examples to predict. All other models in the literature stated above use the complete dataset of interest and they do not carefully select the test examples. Authors are free to cherry pick examples for training, but for a fair comparison with others, they have to sample from the whole ALMANAC dataset for testing.

Minor issues:

1- For the different split schemes, please clarify if the training and validation sets are identical for all models which participate in the ensemble.
2 - Please name the "test" dataset in the Table 2. It is not clear what is the difference between this test set and the analysis performed on DECREASE dataset mentioned in lines 439 - 447.
3 - Figure 2, please add y axis label.
4 - Please rephrase the following sentence: "We considered outliers, the synergy prediction values above or below 10 times the average of the remaining prediction values."

Level of Interest

Please indicate how interesting you found the manuscript: Choose an item.

Quality of Written English

Please indicate the quality of language in the manuscript: Choose an item.

Declaration of Competing Interests

Please complete a declaration of competing interests, considering the following questions:

- Have you in the past five years received reimbursements, fees, funding, or salary from an organisation that may in any way gain or lose financially from the publication of this manuscript, either now or in the future?
- Do you hold any stocks or shares in an organisation that may in any way gain or lose financially from the publication of this manuscript, either now or in the future?
- Do you hold or are you currently applying for any patents relating to the content of the manuscript?
- Have you received reimbursements, fees, funding, or salary from an organization that holds or has applied for patents relating to the content of the manuscript?
- Do you have any other financial competing interests?
- Do you have any non-financial competing interests in relation to this paper?

If you can answer no to all of the above, write 'I declare that I have no competing interests' below. If your reply is yes to any, please give details below.

I declare that I have no competing interests

I agree to the open peer review policy of the journal. I understand that my name will be included on my report to the authors and, if the manuscript is accepted for publication, my named report including any attachments I upload will be posted on the website along with the authors' responses. I agree for my report to be made available under an Open Access Creative Commons CC-BY license

(http://creativecommons.org/licenses/by/4.0/). I understand that any comments which I do not wish to be included in my named report can be included as confidential comments to the editors, which will not be published.

Choose an item.

To further support our reviewers, we have joined with Publons, where you can gain additional credit to further highlight your hard work (see: https://publons.com/journal/530/gigascience). On publication of this paper, your review will be automatically added to Publons, you can then choose whether or not to claim your Publons credit. I understand this statement.

Yes Choose an item.
