# Supplementary material for: SYNPRED: prediction of drug combination effects in cancer using different synergy metrics and ensemble learning
Source: Gigascience. 2022 Sep 26;11:giac087. doi: 10.1093/gigascience/giac087 (PMC9511701; doi:10.1093/gigascience/giac087)
Supplement: giac087_Reviewer_1_Report_Revision_1 — A. ErcÃ¼ment Ã‡iÃ§ek -- 7/7/2022 Reviewed [file giac087_reviewer_1_report_revision_1.pdf]

Reviewer Report

Title: SYNPRED: Prediction of Drug Combination Effects in Cancer using Different Synergy Metrics and Ensemble Learning

Version: Revision 1 **Date:** 7/7/2022

Reviewer name: A. ErcÃ¼ment Ã‡iÃ§ek

Reviewer Comments to Author:

Authors indicate that benchmarking of synergy prediction protocols is a complicated process. While I agree with this. , I do not agree that "..DL architectures as these are not easily applied or not available in GitHub or similar platforms." As the implementations are available on GitHub, the authors can feed their data of choice to easily train these models. There might be some adaptation choices to make such as pruning the data due to unavailability of a certain data modality, or changing the loss function to turn a model into a regressor, but as long as they justify these it should be fine and east. This is what has been done in the literature in DeepSynergy and Matchmaker papers, for instance. They have done this to a degree but I am not convinced that a fair comparison with the literature I made to conclude that this method has clear advantages over others. Authors indicate that they trained 1972 models but I think these are mostly the "baseline" models they use within their ensemble method which does not help in comparing with the literature. Below, I list my detailed comments on the revisions made:
First, while there are many detailed descriptive statistics such as distribution of synergy scores are presented in the main text the comparisons are pushed to the supplement which makes it hard to follow the paper. Also, details on whether or how compared methods are run/trained is missing. For instance in comparison ii), how did you reimplement DeepSynergy, and in Comparison ii) and iv) what data did you use and what hyperparameters did you use to run DeepSynergy and Matchmaker?
In the current revision, in Tables S7 - S12 authors compare their method with some baseline models used in their ensemble such as random forest, KNN and some custom deep learning architectures. I did not ask for this as clearly the problem is complex and a complex model as their method is needed. Nevertheless this is a nice validation which improves the paper.
They compare their method with a reimplemented (please clarify in text) version of DeepSynergy in Table S13 (They say Table S14 but I think it is a typo). First, I strongly suggest they put the result of the final method into this table (and other related ones) for the readability. I had to trace Table 4/5 to see their results and come back to compare with the values in this table. They report better results than DeepSynergy which is good. As they use the review of Kumar et al. later, there are other methods reported in this review which perform better than DeepSynergy in the regression task such as MultitaskDNN, AUDNNSynergy, and Matchmaker. However only DeepSynergy is compared. Later they perform a comparison with Matchmaker but it is ot clear to me why they had to perform a separate comparison instead of comparing them all.
In supplementary table 14, they compare the performance of their method, with performance values of others reported in a review by Kumar et al. This is not meaningful at all as every method use different settings as authors also indicate. Once again, they have to retrain these (or similar) architectures with

their (maybe filtered or postprocessed) data and compare. Just like done for DeepSynergy only in the regression task. They can as well retrain their method with the dataset of these methods as another point of view.

For the results in Tables S15 - S16 (again the table numbers are wrong), author state "... Upon doing this, both CSS and Loewe predictors from SynPred stood very close to the performance of Matchmaker [20],". Matchmaker reports Loewe scores so if no retraining of the model is performed with CSS it is not meaningful to compare these results. I suggest authors to use the CSS scores as the label in training Matchmaker.

In Loewe scores their performance actually does not stand vey close to Matchmaker (MSE vs 123, Pearson .79 vs .73). Only in Spearman correlation their result is 0.01 better.

The details on the dataset used, the train/validation/test splits and number of samples etc. are missing so it is hard to evaluate these scores.

In Table S16, SynPred seems to be 50 times better than all others in terms of MSE. However, while SynPred reports various scores other than ComboScore, the results obtained for other methods are based on ComboScore. Thus, it is comparing apples and oranges and is not meaningful. Again, no training is performed in this comparison. Please do not put results with different metrics into the same table as it is very confusing for the reader.

TYPOs: NCI ALMANC, Matchmakers'

Level of Interest

Please indicate how interesting you found the manuscript: Choose an item.

Quality of Written English

Please indicate the quality of language in the manuscript: Choose an item.

Declaration of Competing Interests

Please complete a declaration of competing interests, considering the following questions:

- Have you in the past five years received reimbursements, fees, funding, or salary from an organisation that may in any way gain or lose financially from the publication of this manuscript, either now or in the future?
- Do you hold any stocks or shares in an organisation that may in any way gain or lose financially from the publication of this manuscript, either now or in the future?
- Do you hold or are you currently applying for any patents relating to the content of the manuscript?
- Have you received reimbursements, fees, funding, or salary from an organization that holds or has applied for patents relating to the content of the manuscript?
- Do you have any other financial competing interests?
- Do you have any non-financial competing interests in relation to this paper?

If you can answer no to all of the above, write 'I declare that I have no competing interests' below. If your reply is yes to any, please give details below.

I declare that I have no competing interests.

I agree to the open peer review policy of the journal. I understand that my name will be included on my report to the authors and, if the manuscript is accepted for publication, my named report including any attachments I upload will be posted on the website along with the authors' responses. I agree for my report to be made available under an Open Access Creative Commons CC-BY license (http://creativecommons.org/licenses/by/4.0/). I understand that any comments which I do not wish to be included in my named report can be included as confidential comments to the editors, which will not be published.

Choose an item.

To further support our reviewers, we have joined with Publons, where you can gain additional credit to further highlight your hard work (see: https://publons.com/journal/530/gigascience). On publication of this paper, your review will be automatically added to Publons, you can then choose whether or not to claim your Publons credit. I understand this statement.

Yes Choose an item.
